# Structural Behavior and Failure Modes of Strengthened Deep Beams Using Fiber-Reinforced Polymers: A Review

**DOI:** 10.3390/polym18212642

**Published:** 2026-10-25

**Authors:** Muhammad Aslam, Yong Jin, Qing Wang, Azlan Shah Ali

**Affiliations:** 1 Department of Civil Engineering, School of Architecture and Energy Engineering, Wenzhou University of Technology, Wenzhou 325000, Zhejiang, China; maslam@wzut.edu.cn; 2 School of Civil Engineering and Transportation, Guangzhou University, Guangzhou 510006, China; wangq@gzhu.edu.cn; 3 Faculty of Built Environment, Universiti Malaya, Kuala Lumpur 50603, Malaysia

**Keywords:** strengthened deep beams, flexural behavior, FRPs, openings, strengthened openings

## Abstract

Deep beams (DBs) play a crucial role in structural systems due to the higher demand for flexural load-carrying capacity, but they also face challenges due to design complexities and stress patterns. This paper reviews the flexural performance of strengthened deep beams in near-surface mounted (NSM) and externally bonded reinforcement (EBR) techniques using steel, fiber-reinforced polymers (FRPs), and hybrid approaches. The analysis of compiled data from the literature focuses on strengthening materials and techniques, flexural behavior, the effect of reinforcement ratios, interfacial bonding, shear-span-to-depth ratios, stiffness, ductility, failure modes, cracking behavior, and analytical models. It was concluded that the hybrid strengthening technique and high-strength jacket techniques in the EBR method have proven to be highly effective for strengthening DBs for flexural behavior. Hybrid strengthening considering different grades of concrete and struts at a 45° angle showed 53–93% higher ultimate loads and 33–70% higher deflection compared to control solid deep beams. Carbon FRP (CFRP) strips and glass FRP (GFRP) fabrics in the hybrid technique NSM-EBR, combined for strengthening of DBs with openings, showed much better results; it increased the ultimate strength and deflections by about 52% compared to solid DBs without openings. Premature debonding and concrete-cover separation may prevent externally bonded reinforcement from achieving its maximum capacity. But an increase in the number of layers or the bonded length does not necessarily mean providing higher strength; the resistance of the surrounding concrete and adequate anchorage are equally crucial.

## 1. Introduction

Structural members such as reinforced concrete deep beams (RCDBs) are the members that carry the loads directly supported by the compression struts joining the loads and supports [1]. ACI 318-14 [2] states that the deep beams (DBs) should be loaded at the loading and supporting points in a way that compression thrusts can grow between these loading and supporting points. RCDBs are considered useful in tall buildings, offshore structures, long-span structures, and complex foundation systems. The moment capacities of DBs are normally adequate and are not of major concern, but their shear capacity is very important to understand because they usually fail in shear at the ultimate loads. DBs are characterized by relatively small values of span-to-depth ratios. Because of their proportions, they develop a mechanism of force transfer quite different from that in shallow beams [3]. In the current scenario of the global construction market, most of the existing RC buildings were designed according to old design standards, which often overestimate RCDBs’ strength and may cause collapse risk [4,5]. In addition, poor reinforcement detailing, such as transverse reinforcements like anchors, stirrups, or smooth bars, and lap splices in the plastic hinge region further increases their susceptibility by promoting brittle failure modes that affect the structural integrity and cause huge life and property losses [6,7]. Furthermore, many reinforced concrete (RC) structures do not meet the required specifications, including increased applied loads, design errors, poor detailing, reinforcement bar corrosion, construction defects, design standard upgrades, natural disasters such as earthquakes [8,9], or harsh environmental conditions, such as deterioration and aging, or poor maintenance [10,11].

Therefore, the structures must be replaced or strengthened to address inadequate capacity and/or retrofitted when some structural members are damaged; furthermore, the maintenance of structural members takes less time and costs less as compared to rebuilding them [12]. In most cases, strengthening is the most environmentally and economically effective solution compared with demolition and reconstruction [13]. Structures made with low-strength concrete are unsafe [14,15,16,17,18], and these types of structures should be properly strengthened using the proper materials and techniques. Several traditional techniques were used in the 20th century for strengthening RC structures, such as concrete and steel jacketing, ferrocement, and steel plates [19,20]. Although there are many strengthening techniques employed for RC beams and structures. Recently, the usage of fiber-reinforced polymers (FRPs) has attained more attention on account of many attractive properties such as good non-corrosivity, high strength-to-weight ratio, high fatigue strength, and ease of constructing and strengthening RC structures, and FRPs require less labor as compared to the other conventional techniques [12]. Several researchers studied the use of FRP sheets, laminates, plates, strips, and bars with strong adhesives to strengthen the RC elements, including columns, beams, slabs, and walls because the FRPs are non-corrosive, non-magnetic, and resistant to various types of chemicals; they are increasingly used for both internal and external reinforcement of the existing concrete structures [3,12].

Similar to conventional RC beams, the deep beams were also strengthened with two methods, namely internal strengthening using near-surface mounted (NSM) or near-surface embedment (NSE) and external strengthening involving the externally bonded reinforcement (EBR) method [21,22,23]. Furthermore, the DBs also contain openings which are often necessary for utilities like pipes, ducts, and cables, but these modifications can significantly weaken the beam. When an opening is introduced, it disrupts the natural flow of forces through the beam, leading to stress concentrations and reducing the overall strength and stiffness of the structure [24,25]. This can make the beam more vulnerable to failure, especially under heavy loads. To address these issues, engineers increasingly rely on FRPs as a strengthening solution. FRPs are lightweight yet incredibly strong materials that can be applied around openings to reinforce the beam. By redistributing stresses, FRPs help restore or even enhance the beam’s structural capacity, making it safer and more reliable [26,27]. Therefore, the deep beams face flexural challenges due to their small span-to-depth ratio, which leads to high shear forces and stress concentrations. Openings for utilities can exacerbate these problems by creating additional stress points and weakening the beam’s structural integrity. Effective reinforcement strategies, such as FRPs in NSM and EBR, are crucial for mitigating these issues and enhancing the beam’s load-carrying capacity [6,28]. Recently, a parametric study was conducted by Yagmur [29] to investigate the performance of strengthened DBs with circular web openings in shear spans. It was reported that increasing the diameter of web openings significantly reduces the structural performance and energy absorption. Further, the application of CFRP can mitigate these effects, and increasing the thickness of CFRP laminates can partially restore the structural performance. An analytical multi-strut model for prediction of load-carrying capacity and failure mechanisms of RCDBs with openings and strengthened with FRP composites was proposed by Rousta et al. [30]. This model improves the potential to simulate the probable different load paths that are generated by strengthening materials and openings.

The flexural behavior of strengthened deep beams is the main emphasis of this study’s narrative review methodology. The literature studies were searched via different databases, including ScienceDirect, Google Scholar, and Scopus, and further searches were made via Google. The research articles were searched and downloaded between May and September 2024, with the last searches being performed in 2026. The main search keywords used were “strengthening of deep beams”, “strengthened deep beams”, “role of openings in deep beams”, “strengthened openings in deep beams” and “flexural behavior of strengthened deep beams”. Studies were selected based on the screening of their titles and abstracts and relevance to the review theme. The primary selection criteria were whether a study investigated strengthening performance for deep beams under flexural loads or offered directly relevant information on load transfer, cracking, bonding, or failure behavior. This framework was used to evaluate experimental, numerical, and analytical investigations; numerical or analytical modeling was not the primary focus of the study. The major scope did not include studies on other structural members or unrelated loading conditions; however, appropriate codes and supporting materials were used to explain definitions and prediction models. The literature comprised relevant codes and design guides, theses, books, conference papers, and journal articles. The selection process was not based on a systematic screening strategy, and exact combinations of Boolean search terms and publication year limitations were not recorded. The reliability of constructing a numerical study-selection flow diagram is compromised due to the lack of records pertaining to the counts of initially recognized records, duplicates, screened studies, and exclusions. These restrictions reduce the reproducibility of the selection process; the review thus offers a targeted summary of the chosen literature, without claiming to cover all aspects related to the topic.

This study investigates the flexural behavior of deep beams strengthened using steel, FRPs, and hybrid systems in the strengthening methods of NSM and EBR. It highlights the published findings to compare different strengthening materials, reinforcement configurations, numbers of reinforcement layers, and anchorage systems affecting the ultimate load, stiffness, and failure modes. In addition, it also examines the effect of openings in deep beams and explores the strengthening arrangements used to increase structural performance around these openings. The alternative strengthening strategies and existing design prediction models are explored to identify their limitations and applicability. The main contribution of this review is to connect strengthening schemes with reported structural performance through a comparative analysis in light of available literature. This paper contributes a synthesis specific to deep beams that relates strengthening configurations to internal load transfer, bond behavior, and reported failure mechanisms. It considers the NSM, EBR, and hybrid techniques in addition to the effect of openings and limitations of prediction models to identify the conditions affecting strengthening effectiveness and the remaining gaps in design guidelines for future research and structural applications.

## 2. Deep Beams in Eyes of Codes

Beams with large depths and spans are called deep beams (DBs), and they are structural members that are supported on a face and loaded on opposite faces such that compressive struts can develop between the supports and loads. They work on either a single force lying at a distance of 2*h* from the support, or the clear span should not be more than four times the depth (*h*) of the member [2]. Further, the DB can also be described as a beam that has a span-to-depth ratio less than 3 [31]. According to Indian Standards [32], DBs are identified by their effective span-to-depth (L/D) ratio: for simply supported beams, *L*/*D* must be less than 2, while for continuous beams it should be less than 2.5. Where D is the overall depth, and L is the effective span, defined as the smaller of the center-to-center distance between supports or 1.15 times the clear span. Unlike standard beams, DBs primarily transfer loads to supports through compression forces rather than bending. Furthermore, ACI 318-99 [33] defines that simple or continuous DBs have a clear span-to-effective depth ratio (*l_n_*/*d*) of less than 5, where *d* is the effective depth and *l_n_* is the clear span length.

### 2.1. Behavior of Deep Beams in Flexure

This study focuses on the flexural aspect and the contribution of longitudinal tension reinforcement and strengthening in DBs under transverse loading, while considering their interaction with strut-and-tie action and the shear. Flexural resistance might be critical when the nodal zones, compression struts, anchorage, and transverse reinforcement remain adequate until either the yielding of longitudinal steel or the flexural compression failure occurs. The governing behavior depends on the combined consequences of loading arrangements, strengthening configuration, span-to-depth and shear-span-to-depth ratios, and the reinforcement ratios. Therefore, ultimate load is considered along with the reported failure mechanism, rather than treated automatically as a measure of the flexural capacity. In flexure, the deep beams behave quite differently from the shallow beams because of their smaller span-to-depth ratios. Due to their smaller span-to-depth ratio, the longitudinal strain distribution is linear through the depth, while the deep beams experience a non-linear distribution due to the effects of applied load and support reactions over a substantial part of the member. The usual assumption that plane sections remain plane after bending under the load is not applicable. In DBs, shear deformation contributes to the response, and the neutral axis shifts toward the tension side as loading and cracking occur [34,35,36]. Researchers [37,38,39] reported that the DBs develop an arch action through which loads are transmitted to the supports along the diagonal concrete compression paths. These diagonal compression paths work together with the longitudinal tension reinforcement to form a strut-and-tie mechanism. The flexural and shear behaviors are consequently closely connected, and the load-carrying capacity depends on the tension reinforcement, compression struts, and their anchorage. Ashour et al. [40] emphasized the importance of reinforcement in DBs. Longitudinal reinforcement carries tensile forces, while vertical and horizontal web reinforcement controls cracking and resists tensile stresses in diagonal compression paths. Further, insufficient reinforcement or inadequate anchorage results in premature failure through crushing of compression struts, anchorage failure, or diagonal cracking. Therefore, careful reinforcement detailing is necessary to increase the deformation capacity and to maintain the structural integrity.

### 2.2. Problems in Deep Beams

Deep beams encounter specific problems when subjected to flexural loading. Their nonlinear strain distribution due to significant depth makes predicting their behavior challenging. They are particularly vulnerable to shear failures, often leading to diagonal cracking if not reinforced adequately. It was emphasized that the deep beams experience high-stress concentrations near supports and load points, increasing the risk of localized failures [39,41]. Additionally, the arch action in deep beams reduces their flexural capacity, limiting their ability to handle bending moments compared to shallow beams [40,42]. To effectively manage these issues, deep beams require careful design and reinforcement to ensure their structural integrity. Unfortunately, there is not a reliable procedure or model for accurately predicting their strength. This is largely due to the complex and nonlinear nature of reinforced concrete behavior, which makes it challenging to foresee failures [43].

### 2.3. Deep Beams with Openings and Types of Openings

The introduction of openings in deep beams represents a major challenge that requires thorough evaluation. The term ‘opening’ in deep beams is defined as the holes or cut-outs formed within the beam web, usually to allow ventilation ducts, pipes, or electrical services to pass through. They can vary in size, shape, and position. By removing concrete in the web, the opening may interrupt the load-transfer paths between applied loads and supports; however, its influence depends on its location relative to the paths, dimensions, and the reinforcement placed around it. If an opening is created to allow for the passage of utility lines, ducts, or pipes, it produces an area of weakness in a beam by disturbing its load path and increasing stress concentrations [28]. Further, the presence of web openings in DBs leads to early diagonal cracking and also causes a significant reduction in the shear strength [22]. Small, round, or square holes for things like conduits create a point of stress but do not weaken the beam as drastically. However, large rectangular, square, and circular holes required for accommodating larger ducts can significantly reduce the shear capacity and stiffness of the beam [44]. This reduction results in non-uniform stress distributions and possible cracking, delamination, or failure if the openings are not sufficiently reinforced. Ashour et al. [40] conducted research on this and found that it may need additional reinforcement to avoid structural failure in the beam, as also observed by Smith & Vantsiotis [45]. It gets a lot harder when there are several holes. Stress interactions between these openings can result in complex failure mechanisms requiring refined strengthening methods. Openings near supports are especially problematic as they interrupt the high-stress regions needed to transfer loads to these, increasing, hence, the risk of shear failure [39]. Web openings either at edges or at mid-span can reduce the stiffness and the load-carrying capacity of the RCDBs, and they increase the deflection and cracking [46].

## 3. Properties of Fiber-Reinforced Polymers (FRPs) and Adhesives

The various types of strengthening materials such as CFRP sheet/laminates, strips, plates, bars/ropes, Sika CFRP wraps hex-230C and 530C, GFRP fabrics, E-glass wraps, GFRP plates, and steel plates were used for the retrofitting and strengthening of deep beams. The CFRP sheets or laminates with a thickness of 0.12 mm have an elastic modulus in the range of 230–240 GPa, a maximum tensile strength found in the range of 3475–4950 MPa, and a rupture strain of about 1.5% [47,48,49]. The CFRP plates with a thickness of 0.12 cm had a maximum tensile strength of 3050 MPa, an elastic modulus of 165 GPa, and an elongation of 1.7% and were preferred for strengthening of DBs [50]. Few researchers used the Sika wrap hex-230C with a thickness of 0.12 cm, with a tensile strength and elastic modulus in the range of 3400–4100 MPa and 230 GPa, respectively [19,50,51]. Sika wraps 530-C CFRP was also used, which has a tensile strength of 3800 MPa and an E-value of 231 GPa [52]. Sika wraps 301-C, also known as CFRP strips, having a thickness of 0.17 mm, tensile strength of 4900 MPa, E-value of 230 GPa, and elongation of 0.021, were also used [53]. Hybrid FRP strips of 3.18 mm thickness made of two carbon and one glass fiber sandwiched together, having a tensile strength of about 825 MPa and an E-value of 62 GPa, were used as strengthening elements in DBs [54,55]. CFRP Sika-wrap FX-50C rope, having a tensile strength of 4000 MPa and an elastic modulus of 240 GPa, was used for strengthening of DBs [56]. The GFRP fabrics prepared with 172 fibers, having a thickness of 0.275 mm, a modulus of elasticity of 16.07 GPa, and an ultimate stress of 290 MPa, were used for the strengthening of DBs [31]. GFRP was also categorized as E-glass woven wraps with a thickness of 0.17 cm, tensile strength of 290 MPa, elastic modulus of 132.5 GPa, and elongation of 1% [50]. The glass fibers, in terms of E-glass and S-glass, were used for strengthening DBs; these materials have a density in the range of 2.5–2.6 g/cm^3^, an elastic modulus that varies between 72 and 87 GPa, and a tensile strength that is found in the range of 1720 to 2530 MPa [28]. The steel plates were also used for the strengthening of DBs, which were 6 mm thick and had a yield stress of 360–380 MPa, whereas the stud connectors used were 8 mm in diameter and had a yield stress of 350 MPa [1,50]. The adhesive or epoxy used for the strengthening process has a tensile strength of 20 MPa and an elastic modulus of about 12.8 GPa [49]. Sikadur-300 and Sikadur-330 were used as strong adhesives, and they have a tensile strength of 40–45 MPa and a tensile modulus of 3500 MPa [50,51,52,53,56]. In general, it was found that high-strength carbon and CFRP materials stand out for their exceptional strength, making them ideal and able to bear a heavy load without much stretching, though they can be brittle. On the other hand, materials like aramid (AFRP) and basalt fibers (BFRP) strike a good balance; they are strong but also flexible enough to stretch before breaking, which is useful in applications. Glass fibers, especially S-Glass, offer decent strength with more flexibility compared to carbon fibers, while mild steel behaves predictably, bending before breaking, which is why it is commonly used in construction.

FRP sheets are still largely used as external reinforcement; however, FRP-embedded reinforcement, grids, and hybrid systems have increased the flexural and shear strengthening options. Yang et al. [57] studied FRP grids embedded in an engineered cementitious composite (ECC) matrix and found that they improved the shear resistance of RC beams. Although the failure was still governed by concrete cover spalling and interfacial debonding, this study highlights that the benefits of the grid system rely on successful load transfer from the strengthening material to the concrete. These methods may thus be suitable for deep beams, but their performance must be evaluated further under the concentrated pressures associated with strut-and-tie action. Other approaches include CFRP ropes installed using NSM and embedded through-section techniques, which have been tested in DBs [58], and deep-embedded CFRP bars, recently investigated in high-strength concrete DBs with web openings [59]. These approaches can reinforce diagonal cracks, but their effectiveness can depend on orientation, anchorage, and contact with the existing steel reinforcement. Care must also be taken not to drill or groove the original reinforcement during installation. Another approach is hybrid systems that combine several types of fibers or reinforcement configurations. Ibrahim et al. [60] observed an enhancement in shear resistance and a reduction in debonding of DBs strengthened with NSM carbon-glass FRP strips. More recently, Thansirichaisree et al. [61] explored the glass-basalt FRP strengthening of DBs with and without openings. Their results indicated that anchors could reduce debonding, but the strengthening did not reliably restore the capacity to that of the control beam. Thus, hybrid systems do not necessarily lead to increased ductility or capacity. The suitability of these systems in DBs should be determined by their ability to control diagonal cracking, to preserve bond and anchorage, and to operate in cooperation with the internal reinforcement, taking into account the controlling failure mechanism. Therefore, further investigations into varied shear-span-to-depth ratios, opening configurations, and reinforcement schemes are needed to provide valid design recommendations.

## 4. Flexural Behavior of Strengthened Deep Beams

Concrete is extensively used in modern construction for a variety of projects, including dams, high-rise buildings, flyovers, and underpasses. It is categorized based on density into types such as reinforced cement concrete (RCC), normal weight concrete (NWC), lightweight concrete (LWC), self-compacting concrete (SCC), and fiber-reinforced concrete (FRC). In the field of deep beams, a total of 446 deep beams were cast using NWC, 20 beams were made with LWC, nine beams with SCC and steel fiber concrete (SFC), and four beams were made with fiber-reinforced self-compacting concrete (FRSCC). The comprehensive analysis of deep beams necessitates an examination of both flexure and shear, as both factors are integral to assessing their structural efficiency. This study focuses on the flexural behavior of DBs; for that, a thorough investigation was performed, and a total of 120 articles were reviewed, and around 480 strengthened and unstrengthened deep beams of varying dimensions were studied. Notably, 129 deep beams were unstrengthened and demonstrated substantial resistance to flexure. The strengthened DBs were about 350, among which 129 were strengthened using the near-surface mounted (NSM) technique, 210 contained externally bonded reinforcement (EBR) technique, and 11 were strengthened using composite techniques such as NSM and EBR combined.

### 4.1. Strengthening Techniques and Tested Deep Beams

**Figure 1:**
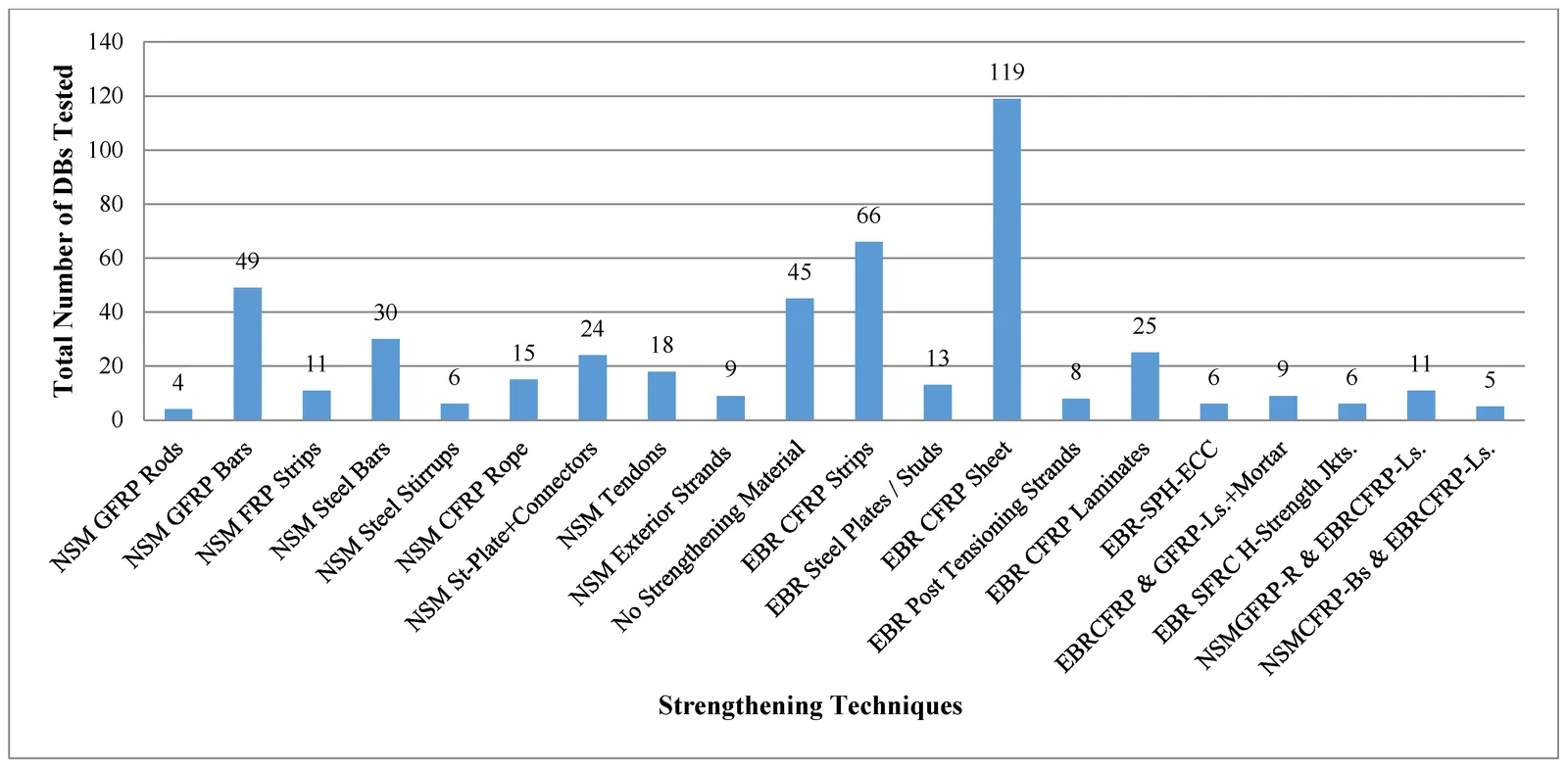
Strengthening techniques and number of DBs tested.

Near-surface mounted (NSM) technique involves embedding reinforcement bars or sheets, made of steel or FRPs with consistent cross-sections, into the structure to enhance its strength in the flexural zone. Similarly, the externally bonded reinforcement (EBR) technique involves bonding steel or FRP laminates or sheets externally to the tension surface of the member using epoxy as an adhesive. Various researchers have employed different materials, such as rods, laminates, plates, post-tensioning strands, steel plates, and connectors and tendons, to improve the flexural behavior of concrete beams using NSM, EBR, and their combined applications. Among all studies, 45 DBs were considered control specimens without any strengthening material. The deep beams strengthened using steel components, including traditional steel bars, plates/studs, and stirrups, were 30, 13, and 6, respectively. The beams strengthened with GFRP rods and bars were 53 specimens, and CFRP rope-strengthened beams were about 15 specimens. Approximately 200 deep beams were strengthened using CFRP composites in the form of laminates (25 specimens), sheets (119 specimens), and strips (66 specimens) as an external reinforcement. Approximately, 200 deep beams were strengthened using CFRP composites in the form of laminates (about 25 specimens), sheets (119 specimens) and strips (66 specimens) as an external reinforcement. Moreover, 12 beams were strengthened with special materials; six specimens were strengthened with Steel and Polyvinyl-alcohol Hybrid Fiber-Reinforced Engineered Cementitious Composite (SPHECC), and six were strengthened with high-strength jackets. Figure 1 shows the number of deep beams strengthened in specific strengthening configurations and techniques as described in the reviewed studies.

### 4.2. Research Progress of DBs in EBR Technique

The externally bonded reinforcement (EBR) technique is widely adopted for its simplicity, rapid installation, and effectiveness in strengthening RCC structures. This method commonly employs FRP composites to enhance the flexural and shear performance of structural members, with epoxy adhesives facilitating the bond between the FRP sheets and the concrete surface. Despite its advantages, a significant challenge associated with EBR arises when reinforced structural beams experience sudden failure, often due to premature debonding of the FRP sheet, which prevents the material from reaching its full load-bearing potential.

#### 4.2.1. CFRP Sheets/FRP Composites in EBR

**Figure 2:**
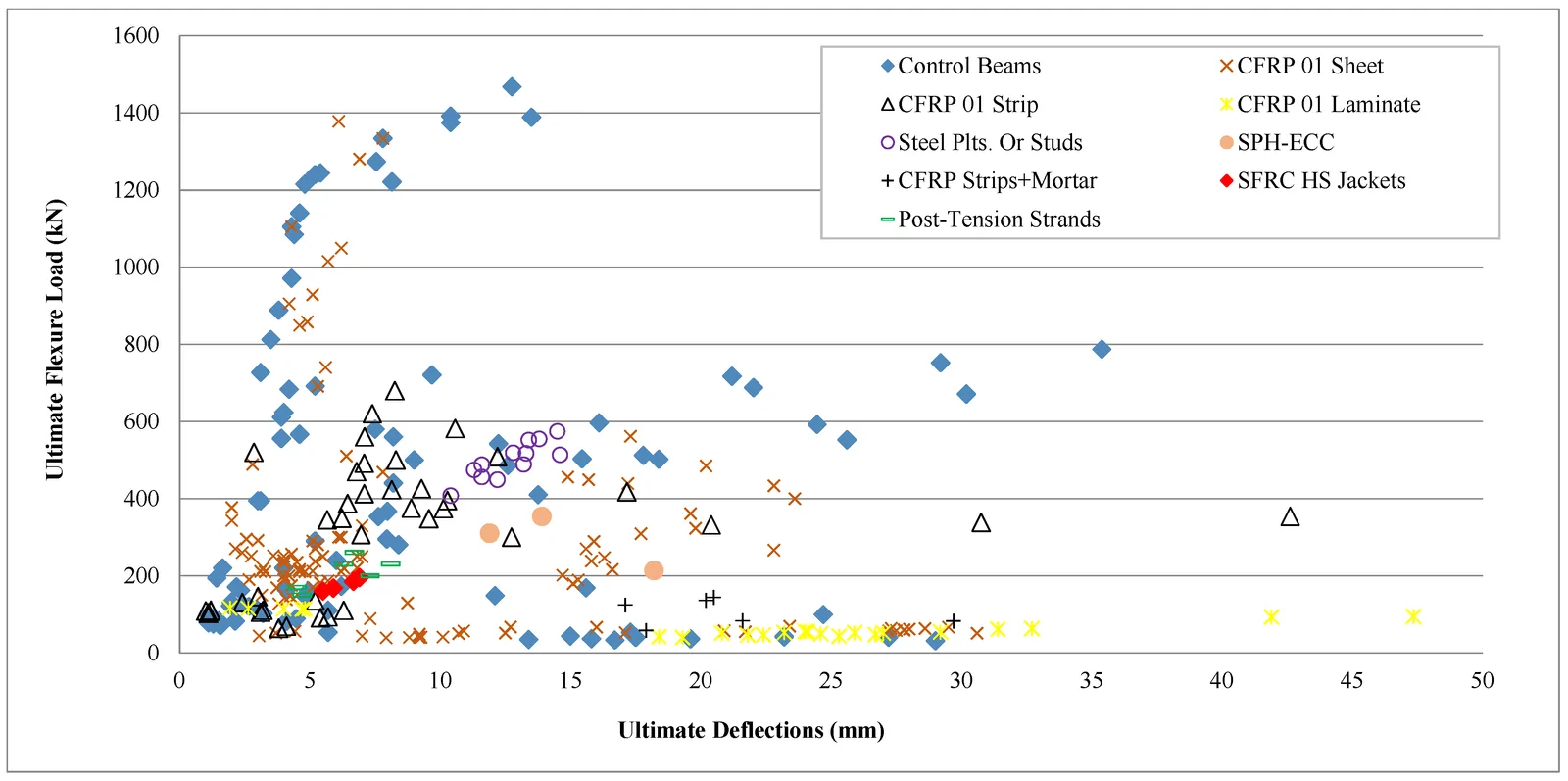
Relationship between ultimate load and ultimate deflection in EBR [1,47,49,51,62,63,64,65,66,67,68,69,70,71,72,73,74,76,77,78,79,80,81,82] (Note: Each strengthened specimen is compared to its corresponding control within the same study. This is because specimens and test conditions vary between studies, making it impossible to directly compare them).

For the flexural strengthening of DBs, the CFRP sheets were employed using the EBR technique. The detailed data collected for different parameters in the EBR technique are presented in Appendix A, and the relationship between the ultimate loads and deflections of strengthened DBs is shown in Figure 2. Sabzi et al. [49] conducted a comparative study on 14 DBs reinforced with CFRP sheets and fiber-reinforced cementitious matrix (FRCM) composites. The deep beams, measuring 1500 × 200 × 352 mm, had CFRP sheets applied directly beneath them in 2 to 3 layers, with each sheet having a thickness of 0.167 mm, along with a mortar and epoxy coating. The results showed a significant enhancement in the beam performance, with a 41% increase in flexural strength and a 29% increase in ultimate displacement (Figure 2). Similarly, Wang et al. [62] performed flexural strengthening of 25 narrow DBs using hybrid anchored CFRP sheets. These beams, with a cross-section of 100 × 180 mm and a length of 1.8 m, had CFRP sheets of 0.167 mm thickness (applied in 1 to 2 layers) affixed to their bottom surfaces, also coated with epoxy. This method improved the flexural load capacity by 34% with a single layer and by 45% with two layers. Ultimate displacement exhibited a decreasing trend, reducing by 5% with a single CFRP sheet layer and by 14% with two layers. Furthermore, Jasim et al. [47] analyzed the behavior of 18 DBs with web openings and strengthened with CFRP sheets. Two DBs were control beams with no openings (NO) and unstrengthened, whereas 16 beams contained two rectangular openings (Ops), and among them, eight were reinforced with CFRP sheets of 0.167 mm thickness on each side of the openings (Appendix A). The dimensions of DBs were 150 × 500 mm, with a length of 1.5 m. The results showed an 8% lower ultimate flexural load in unstrengthened DBs with openings, and a reduction of about 34% in DBs with strengthened openings, compared to the control solid beams. Further, Ghoniem [63] conducted a study on flexural capacity prediction of nine fibrous recycled aggregate concrete beams strengthened by side CFRP sheets in 1–2 layers having 0.17 mm thickness. Results illustrated from testing of the beams show a 27% and 41% increase in ultimate strength with 1 and 2 EBR sheets when compared with control beams.

Similarly, Nedjhioui et al. [64] investigated the flexural behavior of hollow deep T-beams strengthened with CFRP sheets. DBs were measured as 1100 × 100 × 160 mm and included a single rectangular opening and were reinforced with a single layer of CFRP sheets (1.2 mm thick) applied directly beneath the beam and coated with epoxy. The results demonstrated a significant improvement in the performance of DBs with openings, showing a 26% increase in flexural strength and a 50% reduction in ultimate displacement compared to the control DBs. Likewise, De-Freitas Arcine et al. [65] performed a numerical analysis to explore the interaction between stirrups and CFRPs for the strengthening of DBs. Six beams with dimensions of 2500 × 120 × 250 mm, each containing 11 rectangular openings of 20 × 20 mm size were strengthened with CFRP of 0.167 mm thickness with epoxy around the openings. It was found that the improvement in both ultimate load and ultimate displacement was about 47% and 225%, respectively, compared to the control beams (Figure 2). Furthermore, in another study, the reverse mechanism was found; Hassan et al. [66] investigated the rehabilitation of reinforced concrete deep beams using CFRP and strengthened them with CFRP sheets of 0.17 mm thickness, with beam dimensions of 200 × 500 mm and a length of 1.8 m. The findings revealed a 7% reduction in ultimate flexural load, but the ultimate deflection was increased by about 11% in comparison to control beams. Furthermore, Alshamrani et al. [67] studied the seismic flexural behavior of CFRP-strengthened DBs secured with fiber anchors. In this study, four beams were strengthened with CFRP sheets and splay anchors with a cumulative thickness of 6 mm (Appendix A). The results indicated a 48% increase in ultimate flexural load and a 49% reduction in ultimate displacement for the strengthened beams compared to control beams.

#### 4.2.2. EBR on Grooves

Codina et al. [68] investigated the flexural behavior of 20 DBs strengthened with EBR CFRP sheets over the grooves. The beam dimensions were 140 × 180 mm and a length of 2.4 m; four DBs were unstrengthened and had no openings, five were unstrengthened but had grooves, and 11 had grooves and were strengthened at the bottom with CFRP laminate 1.4 mm thick (Appendix A). The results indicated a 40% increase in ultimate flexural load for unstrengthened beams and a 46% increase for strengthened beams. Additionally, there was a 38% and 57% increase in ultimate displacement for unstrengthened and strengthened beams, respectively, compared to the control beams (Figure 2). Similarly, Arabzadeh and Karimizadeh [69] conducted an experimental study on DBs with openings using FRP composites as EBR and EBROG methods. This study tested 10 beams, two of which had no openings, while eight had 1–2 circular openings in the center. These beams were strengthened with CFRP strips, 0.9 mm thick, applied vertically, horizontally, and at a 45° angle near the openings. The CFRP laminate, 1.4 mm thick, was also applied to the bottom of the beams. The results showed a 20% decrease in ultimate flexural load and a 15% decrease in ultimate displacement for the strengthened beams compared to the control beams.

#### 4.2.3. CFRP Strips/Sheets at Angles

Al-Ahmed and Al-Jaburi [51] investigated the behavior of DBs strengthened with CFRP sheets and strips; a total of seven beams with a cross-section of 150 × 240 mm and a length of 1.1 m were tested. Six DBs were strengthened with CFRP strips of 0.15 mm thickness and applied in varying quantities from 4 to 10 strips in a U-wrap configuration and inclined at a 45° angle; details are mentioned in Appendix A. It was found that the DBs strengthened with U-wrap CFRP sheets and those with CFRP strips inclined at 45° exhibited an increase in ultimate load-carrying capacity by 12% and 7%, respectively. Additionally, the U-wrap CFRP sheets showed an 11% increase in ultimate displacement, whereas the CFRP strips inclined at a 45° angle resulted in a 5% decrease in ultimate displacement compared to the control beam. Further, Hanoon et al. [70] investigated the effectiveness factor of the strut-and-tie model for RCDBs strengthened with CFRP sheets. The beams were strengthened using 0.17 mm thick CFRP sheets applied in the vertical direction, along with two bolted struts arranged at a 45° angle. The results revealed a 44% increase in ultimate flexural load and a 13% decrease in ultimate displacement compared to control beams. Additionally, Abed and Al-Sulayfani [71] investigated the effect of openings on the behavior of 13 DBs using CFRP laminates. Twelve beams had two circular openings, six of which were reinforced with CFRP sheets of 0.17 mm thickness applied in diagonal, perpendicular, and horizontal directions. The beams with a cross-section of 120 × 750 mm and a length of 1.8 m showed a decrease in ultimate flexural load by 14% in unstrengthened beams with openings and a 29% increase in strength in strengthened beams with openings, compared to the control solid beams. Similarly, ultimate displacement increased by 106% and 62%, respectively, in unstrengthened beams with openings and strengthened beams with openings, compared to control solid beams. Further, Hawileh et al. [72] performed nonlinear FEM on two solid DBs with two rectangular openings, which were strengthened using EBR CFRP sheets with a thickness of 0.167 mm and were applied horizontally and vertically near the openings. The results from the beam testing revealed a 3% increase in ultimate strength, accompanied by a 15% decrease in ultimate displacement compared to control beams (Figure 2).

#### 4.2.4. CFRP Strips and GFRP Fabrics in EBR-NSM

Hadi et al. [73] conducted a full-scale evaluation of the flexural strength and ductility of RCDBs strengthened using various FRP mechanisms. A total of 13 beams were tested, each containing two square openings of varying sizes at the top, center, and bottom of the beam near supporting edges. Two DBs were considered as controls, six were unstrengthened, and five were strengthened with CFRP sheets of 0.167 mm thickness (Appendix A). The results indicated a 61% reduction in the ultimate flexural load for unstrengthened beams with openings, whereas the strengthened beams with openings showed a 22% increase in ultimate flexural load. Additionally, the ultimate displacement decreased by 11% for unstrengthened beams and by 2% for strengthened beams with openings when compared to control beams (Figure 2). Similarly, El-Sherif and Wakjira [74] examined the DBs in flexure using a hybrid NSM-EB cementitious matrix. A total of 10 beams were tested, eight of which had a single rectangular opening and were strengthened with CFRP strips of varying thicknesses ranging from 0.0115 to 0.066 mm. The beams had a cross-sectional dimension of 150 × 260 mm and a length of 2.5 m. The results indicated a 51% increase in ultimate flexural load for the strengthened beams, although there was a 19% decrease in ultimate displacement compared to the control beams. Further, Wakjira and Ebead [75] investigated the impact of internal transverse reinforcement configurations using EB/NSE-FRCM techniques. Their study included two control beams without openings and 10 beams with a single circular opening, 12 of which were reinforced with three GFRP bars of 16 mm diameter at the bottom, two GFRP bars of 8 mm diameter, and stirrups spaced at 100 mm intervals. Additionally, CFRP strips with thicknesses ranging from 0.0115 to 0.066 mm were applied on each side of the beam with 95 mm center-to-center spacing. These beams, measuring 150 × 330 mm in cross-section and 2.1 m in length, exhibited a 34% increase in ultimate flexural load in the beams with strengthened openings compared to the control solid beams. A similar trend was observed in ultimate displacement, with a 52% increase in the strengthened beams with openings relative to the control solid beams (Figure 2). Khir Allah et al. [76] investigated the use of geopolymer and cement-based fabric-reinforced matrix composites for flexural strengthening, and in a central groove, the NSM-GFRP bars were used in a central groove in combination with EBR sheets/strips were used. The findings revealed a 16% increase in ultimate flexural load for the strengthened beams with groove openings. Additionally, there was an 8% reduction in ultimate displacement when compared to the control beams.

#### 4.2.5. Steel Plates and Connectors/Studs in EBR

The innovative technique using steel plates and studs for strengthening DBs was introduced by Abdul-Razzaq et al. [1]. They prepared 13 beams: one without openings, three had two square openings at the center, three beams had two circular openings at the center, and six had two rectangular openings at the center. A total of nine beams were strengthened with 6 mm steel plates and 8 mm thick steel studs, as mentioned in Appendix A. Steel plates were applied in a U-shape at the starting and ending compression zones, whereas the studs were placed near the openings to improve the flexural capacity of DBs. It was found that the ultimate capacity was reduced by about 21% and 23% for unstrengthened and strengthened DBs with openings, respectively, whereas the reduction in displacement was about 18% for both cases as compared to control DBs (Figure 2). Further, Farouk et al. [77] also introduced a new technique for DBs considering circular and rectangular openings. A total of 24 DBs were prepared; among those, 23 were prepared with openings, and out of them, 13 were strengthened with steel plates and connectors. It was found that the ultimate capacity was reduced by 19% for unstrengthened beams with openings and 41% for strengthened DBs with openings compared to solid control DBs without openings. However, this combination of steel and connectors significantly improved the ultimate displacement of DBs, which was about 26% and 104% for unstrengthened and strengthened beams with openings. Teng et al. [78] utilized high-strength steel tendons in EBR for pre-damaged DBs in flexure, and a total of 18 beams were cast, five of which had circular openings. This strategy only increased the ultimate capacity by 4% for strengthened beams without openings, and ultimate displacement was increased by about 173% compared to control DBs (Figure 2). This shows that the application of steel plates with studs and connectors, and high-strength steel tendons in EBR, reduces the ultimate deflection, but the deflection was significantly increased by about 100–175% compared to control beams, which increases the energy absorption and deflection of DBs.

#### 4.2.6. High-Strength Jackets and Hybrid Strengthening in EBR

The hybrid strengthening method using high-strength jackets and a combination of FRP materials was introduced by a few researchers. Ibrahim et al. [79] explored the behavior of strengthened DBs using FRP jackets and prepared six beams of length 1.9 m and cross-section of 120 × 300 mm, and five beams were strengthened with 30 mm thick hybrid-strengthened jackets (Appendix A). It was found that this technique significantly increased the ultimate capacity of strengthened DBs by about 93%, and deflection increased by about 33% as compared to control DBs (Figure 2). Similarly, another study by Shakir and Hanoon [80] used a curved hybrid strengthening system for precast beams under static loads. A total of 11 beams were prepared, having a length of 2 m and a cross-section of 180 × 450 mm. The interesting thing is that combining conventional and low-grade concrete in the DBs is an innovation that opens a new direction for the researchers. In hybrid models, they also used struts at 45° angles, and it was found that the ultimate capacity was increased by about 54% and deflection increased by 70% compared to control unstrengthened DBs. Generally, it was found that the hybrid strengthening technique and high-strength jacket techniques in the EBR method have proven to be highly effective methods for strengthening DBs in both flexural and shear behavior. Hybrid strengthening approaches, such as those incorporating lower-strength concrete at the bottom, high-strength concrete at the top, and two struts inclined at a 45° angle, have demonstrated significant improvements in the ultimate load and deflection of the DBs. Additionally, the application of steel fiber-reinforced concrete (SFRC) jackets in the tension zone of DBs not only enhanced the loading capacity but also improved displacement and stiffness.

#### 4.2.7. Post-Tensioned Strands in EBR

Al-Ahmed and Khalaf [81] examined the behavior of 09 RCDBs with large openings and strengthened with external post-tensioning strands. The dimensions of the beams were 1.6 m in length, and the cross-section was 250 × 400 mm; the opening size was 320 mm from each edge of the beam. Six DBs were strengthened using post-tensioning strands oriented in both horizontal and vertical directions, and the dimensions of the anchorage plate were 250 × 80 × 30 mm, whereas the strands had a diameter, cross-sectional area, and ultimate tensile strength of 12.7 mm, 98 mm^2^ and 1630 MPa, as shown in Appendix A. It was concluded that the ultimate flexural strength was reduced by 21%, whereas the deflection was increased by only 6% as compared to control specimens with openings (Figure 2).

In the EBR strengthening technique, it was found that its successful implementation depends on both the reinforcement material and its ability to transmit forces to the deep beam. The CFRP sheets generally increase the ultimate load, while the addition of a second layer does not result in a proportional increase in the ultimate capacity. This shows that additional reinforcement may provide limited benefits when bonding or anchorage governs failure. Similarly, the influence of inclined strips, U-wraps, and reinforcement around openings relies on their position relative to the interrupted load paths and critical cracks. Further, the additional anchorage could increase force transfer, but the available studies do not establish a reliably greater arrangement. The steel tendons, plates, and connectors showed mixed results in terms of loading capacity, although higher displacement was achieved by these materials. These increments should be carefully considered because the ultimate displacement alone is insufficient to establish energy absorption or higher ductility in the members. In addition, the hybrid and jacketed systems showed higher ultimate capacities, but these increments also showed changes in the load-transfer mechanism and the beam section. The selection of the control beam is also important because the strengthened beam with openings may remain weaker than a solid beam while still performing better than the unstrengthened beam with openings. Further, due to variations among studies in terms of differences in opening position, beam’s geometry, prior damage, and internal reinforcement, these techniques cannot be directly compared. Therefore, based on the reviewed literature, no single strengthening system can be considered the best in all cases.

### 4.3. Research Progress of DBs in NSM Technique

The near-surface mounted (NSM) technique was initially introduced in the 1940s for strengthening structural elements. It significantly enhances the load-carrying capacity, and deformability. Effective application of the NSM technique requires careful consideration of various factors, including the type of reinforcement, groove dimensions, and fillers.

#### 4.3.1. GFRP Bars in NSM

**Figure 3:**
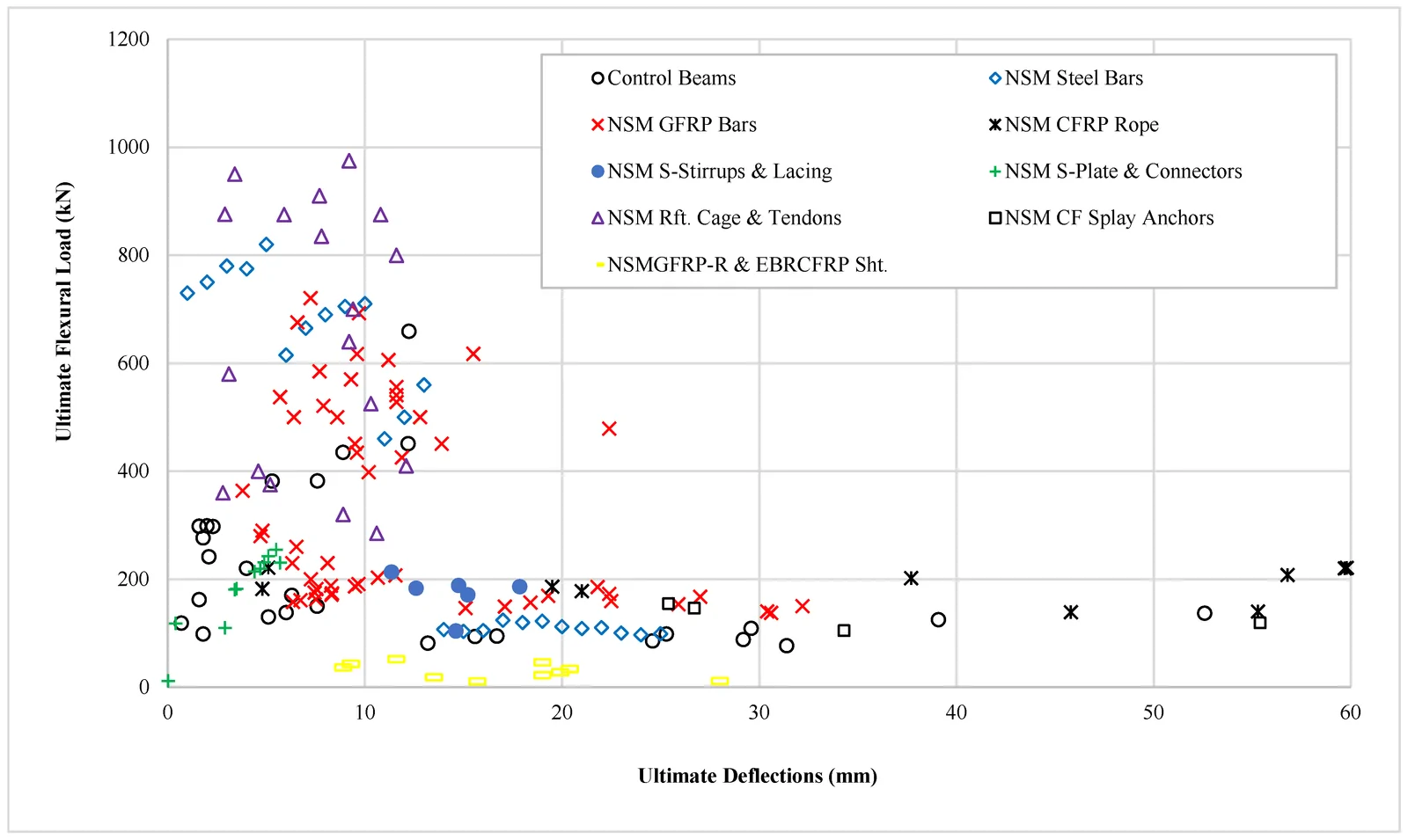
Relationship of ultimate load and ultimate deflection in NSM [49,58,74,83,84,85,86,87,88] (Note: Each strengthened specimen is compared to its corresponding control within the same study. This is because specimens and test conditions vary between studies, making it impossible to directly compare them).

To validate the effectiveness of the NSM technique for strengthening, Sabzi et al. [49] conducted a comparative analysis of DBs strengthened with FRP sheets, FRCM composites, and GFRP bars in NSM. In their study, 14 deep beams, each with cross-sectional dimensions of 250 × 300 mm and a length of 2.2 m, were tested, as shown in Appendix A. The results indicated that the use of near-surface GFRP bars shifted the failure mode from brittle tension failure to compression failure, with a 41% increase in flexural strength and a 29% increase in ultimate displacement, as highlighted in Figure 3. Similarly, Nassif et al. [83] investigated the flexural behavior of high-strength concrete DBs reinforced with GFRP bars, testing a total of eight beams of 1.8 m in length and a cross-section of 150 × 500 mm. The findings showed a 13% decrease in flexural strength and a 31% reduction in ultimate displacement with the use of near-surface GFRP bars. Further, the flexural strengthening of fire-damaged DBs with GFRP bars was explored by Jafarzadeh and Nematzadeh [84]. A total of 10 beams were prepared with dimensions of 150 × 150 mm and a length of 1.1 m. Two GFRP bars of 10 mm diameter were embedded in the tension zone, resulting in a 9% increase in ultimate flexural load and a 19% decrease in ultimate displacement. The hybrid approach was proposed by El-Sherif et al. [74] by combining NSM and EBR fabric-reinforced cementitious matrix. In their experiments, two 8 mm GFRP bars were placed at the top, while 10 to 16 mm Ø bars were positioned in the tension zone with a clear cover of 50 mm. The beams measured 2.5 m in length, 150 mm in width, and 260 mm in depth, with one rectangular opening at the bottom of the beam (Appendix A). The results demonstrated a significant improvement, with a 160% increase in ultimate flexural load and a 7% decrease in ultimate displacement (Figure 3). It was found that the GFRP bars in NSM significantly improved the flexural performance of DBs, and using bars of 10–16 mm in both tension and compression zones showed significant improvements in ultimate load capacity while simultaneously reducing displacement, variability, and stiffness. The findings suggest that GFRP bars offer superior performance as reinforcement in the flexural zone compared to other strengthening materials, reinforcing their effectiveness as a method for flexural strengthening of concrete beams in the NSM technique.

#### 4.3.2. Steel Bars in NSM

Kammona and Al-Issawi [85] investigated the flexural and shear capacity of RCC deep beams strengthened using the NSM technique with steel bars. Their experimentation involved testing 14 beams, each with a cross-sectional dimension of 200 × 352 mm and a length of 1.5 m. These beams were reinforced with 16 mm steel bars arranged in parallel, perpendicular, and at 30° and 45° angles, extending from the tension zone to the compression zone (Appendix A). The results showed a 4% decrease in average ultimate load compared to the control section. Similarly, Qasim et al. [86] examined the DBs under flexural loading using hybrid fiber-reinforced engineered cementitious composites on beams with a cross-section of 200 × 325 mm and a length of 3.5 m. An additional 03 beams were reinforced with five 16 mm Ø bars in the tension zone, while the compression zone bars were similar to those in the control beams, supplemented with steel and PVA gauges (Appendix A). The testing revealed a consistent 74% increase in ultimate load and a 6% reduction in ultimate displacement compared to the control beams (Figure 3). It was found that the steel bars in NSM showed promising results in terms of increased ultimate load capacity and reduced variability and stiffness by incorporating steel bars and steel-PVA gauges with thicknesses of 16 mm and 12 mm in the tension and compression zones of concrete deep beams. It is hypothesized that these steel reinforcements and gauges in the flexural zone deliver superior performance compared to other strengthening materials, thereby affirming their effectiveness as a method for flexural reinforcement of concrete beams.

#### 4.3.3. Stirrups and Steel Lacings in NSM

Al-Gasham et al. [87] investigated the steel stirrups and lacings for the flexural behavior of DBs with a length of 1.53 m and a cross-section of 150 × 240 mm. Three tension reinforcement ratios were utilized: 0.33, 1.13, and 2.01%, and lacing bars were provided on both sides of the beams, placed opposite to each other to bind the longitudinal bars, forming a sequence of contiguous diamond shapes. The spacing of these diamonds varied with the lacing angle; it was 360 mm at 30°, 210 mm at 45°, and 120 mm at 60°, center to center (Appendix A). The results revealed a 3% increase in flexural strength and a 6.5% decrease in ultimate displacement with the use of near-surface stirrups and lacing (Figure 3). It can be said that the incorporation of lacing and stirrups has shown a promising increase in ultimate load capacity, particularly when using 1.13% tension reinforcement in conjunction with lacing spaced at 210 mm and angled at 45°.

#### 4.3.4. CFRP Ropes in NSM

Murad and Abu-Alhaj [88] studied the flexural strengthening and repair of heat-damaged RC beams using continuous NSM CFRP ropes. Their research involved testing 10 beams, each measuring 1.2 m in length with cross-sectional dimensions of 150 × 250 mm. The beams were reinforced with two 10 mm Ø steel bars in both the compression and tension zones, complemented by 10 mm Ø stirrups spaced at 50 mm intervals. Grooves with a 2.5 cm Ø were cut into the beams, into which 12.5 mm Ø CFRP ropes were inserted and secured with high-strength epoxy. The length of the CFRP ropes ranged from 450 mm to 800 mm in the tension zone, extending into the compression zone at a 45° angle (Appendix A). The test results indicated a 43% increase in ultimate flexural load and a 12% reduction in ultimate displacement compared to the control beams, as shown in Figure 3. Additionally, Chalioris et al. [58] explored a novel strengthening technique for DBs utilizing CFRP ropes as transverse reinforcement; a total of five beams were tested, each with dimensions of 150 × 300 mm and a length of 1.6 m. The beams were reinforced with 2 × 14 mm Ø steel bars in the compression and tension zones, along with 8 mm Ø stirrups spaced at 50 mm intervals. Inclined and vertical holes, each 15 mm Ø, were drilled through the depth to the center of the beam. Circular square grooves were also created at the top and bottom of the web for the effective anchorage of the CFRP ropes. These grooves were partially filled with epoxy resin, and 10 mm Ø CFRP ropes were carefully inserted, forming a spider web-like pattern. The results revealed a 31% increase in flexural strength and a 34% decrease in ultimate displacement compared to the control section (Figure 3). It was found that a novel approach in NSM reinforcement, utilizing CFRP ropes, has been introduced and tested by researchers, demonstrating a notable improvement in flexural behavior and ultimate load capacity of concrete deep beams. Beams reinforced with 10 mm diameter CFRP ropes achieved ultimate load levels comparable to those of control beams, while effectively controlling ultimate displacement. The use of CFRP ropes in NSM was found to be particularly advantageous due to their high modulus of elasticity, optimizing stress distribution more efficiently than other reinforcement methods.

In the NSM technique, the GFRP bars showed mixed results, with some reporting an increase in ultimate capacity, while others showed a reduction in both load and displacement. Based on these findings, the GFRP was found to be inconsistent in increasing the load capacity in comparison to other reinforcement types. Similarly, the hybrid approach NSM-EBR showed a substantial increase in the ultimate load; therefore, its performance depends on the combined technique instead of NSM GFRP bars alone. Furthermore, the performance of steel bars relies on their arrangement and their interfacial bonding with the concrete and adhesive. The steel lacings and stirrups provide a small increase in strength; therefore, their importance should only be considered for transverse restraint and cracking control instead of an increase in ultimate load. The CFRP bars increase the load-carrying capacity, but their performance depends on their bonded length, anchorage, and orientation in the member. In all these configurations, the groove dimensions, concrete cover, and adhesive properties affect the force transfer and may cause concrete cover or bond failure in the beam. It should be carefully analyzed for displacement: a lower displacement at failure does not necessarily indicate higher stiffness, whereas a higher displacement alone does not demonstrate superior ductility. Therefore, to achieve a meaningful comparison, complete details are required: load-deflection curves; specimens must be tested under the same conditions, and failure observations should be similar. Therefore, the NSM reinforcement should accordingly consider the beam’s governing weakness and detailed configurations, rather than rely exclusively on the indicated percentage increase in ultimate strength.

## 5. Effect of Reinforcement Ratios and FRP Lengths on Strengthened DBs

### 5.1. Interfacial Bonding and FRP’s Length, Direction and Location

In strengthening structural members, the interfacial bonding between strengthening materials and adhesives plays a crucial role; in case of insufficient interfacial bond strength, the strengthened beams face premature debonding failure [89,90]. The hybrid fiber-reinforced engineered cementitious composites were used for strengthening DBs, and it was found that some specimens failed due to premature debonding, but with the proper application of hybrid fibers, this failure mode was transformed into concrete crushing and pure bending failure. The bonding lengths of CFRP materials on the strengthened members in flexure play an important role in bending. Four bonding lengths of 700, 900, 1100, and 1400 mm were provided, and it was found that increasing the CFRP bond length in DBs changed the failure of the beams from debonding to immediate debonding. This mainly occurred due to the redistribution of stresses along the interface, causing failure at mid-span instead of at the FRP sheet ends. It was also found that as the FRP length increased, the strengthening efficiency reduced, which was about 4.3% for 700 mm bonding length, 15.4% for 900 mm, 20.5% for 1100 mm, and 23.4% for 1400 mm bonding length [62].

In another study, it was found that the DBs strengthened with CFRP sheets showed significantly higher performance as compared to control beams. At the fiber direction of zero degrees, the quarter-length FRP sheets increased the load-carrying capacity of members by about 15%, half-length FRP sheets showed 31% better results, and full-length FRP increased the capacity of strengthened DB by about 66%. Furthermore, it was observed that the fiber direction also increases the ultimate loads of the strengthened DBs in all the CFRP strengthening lengths; two horizontal CFRP laminate plies provide higher shear resistance of the inclined compression strut by the arch action [48]. The combination of both techniques, NSM and EBR, with CFRP laminates over grooves was also investigated for DBs in the flexural capacity, and it was observed that the ultimate loading capacity and bonding ability were increased in comparison to control and only EBR-strengthened members. However, no significant improvement in flexural capacity was observed if the number of grooves was increased from 2 to 3 grooves [68].

The location, spacing, and diameter of NSM steel bars significantly improve the ultimate load-carrying capacity of the member, especially when it is provided against the diagonal crack path. Therefore, it was found that the DBs strengthened with inclined bars showed two times higher ultimate loads in comparison to vertical bars, and the failure mode of DBs was also transformed from brittle to ductile flexural failure [91]. The effect of steel bars in the NSM strengthening technique also improved the maximum load-carrying capacity of the deep beam. It was found that using eight vertical bars at 150 mm *c*/*c* distance in the shear span region improved the performance of DBs by about 7.5% and 8.2% as compared to control beams. However, as these vertical stirrups were changed to 45 degrees, the load-carrying capacity in the same shear span regions improved to 14% and 14.7%. Further, if this spacing is reduced to 100 mm, the load capacity was increased by about 5.8%. The main achievement was the transformation of the failure mode from brittle shear to ductile flexural when vertical bars were provided at 45 degrees in the member [91]. Flexural strengthening of DBs was also performed with CFRP ropes; it was found that using two continuous CFRP ropes or bars in NSM along the length of the member increases its flexural capacity by about 60%, and stiffness was increased by about 78%; however, the ultimate deflections were significantly reduced compared to control specimens [88]. In deep beams, the conventional steel reinforcement was also replaced with GFRP bars as main reinforcement of 8 mm, 10 mm and 12 mm diameters having tensile strengths of 650, 740, and 1075 MPa, respectively. It was found that the GFRP bars decreased the deflection by about 20%; further, if the compressive strength of concrete was increased, the deflection of DBs was further reduced by about 39% as compared to control beams; however, the ultimate capacity was slightly increased by only 4% [83]. The thickness of FRP materials also plays an important role, as it was found that by increasing CFRP thickness, the ultimate strength of DBs was improved, but it reduced the energy dissipation and ductility in the tested specimens [67].

It can be said that reliable strengthening requires proper reinforcement arrangement and reliable force transfer to restrain the critical cracks in DBs. A longer bonded length can increase the anchorage strength, but there are still chances of debonding, specifically when cracking creates stress concentrations along the interfacial surface. Therefore, the strengthening pattern and observed failure mode should be evaluated together with the benefits reported with longer CFRP sheets. Similarly, increasing the number of grooves from two to three has little benefit, indicating that once another failure mechanism becomes dominant, strengthening the bond alone may not yield significant benefits. When inclined NSM bars cross the main diagonal cracks and limit their opening, they can be more beneficial than vertical bars. While restricting deformation capacity, increasing the thickness or quantity of reinforcement may also increase ultimate load. These findings emphasize the importance of evaluating bond quality, reinforcement configuration, and failure behavior together rather than relying only on the increase in load capacity to determine the success of strengthening.

### 5.2. Effect of Compressive Strength, Steel Ratio and Lacing Angles

The DBs were prepared with three different grades of concrete 30, 40, and 50 MPa and were strengthened with hybrid strengthening FRP sheets of 900 mm length, and it was found that as concrete strength increased, the ultimate loading capacity of the member also increased. The strengthening efficiencies for the strengthened DBs for grades 30, 40, and 50 MPa were 40%, 52%, and 56%, respectively. The higher strengthening efficiencies were due to the higher height of the compression zone, which decreased as the strength increased; therefore, the ability of the beam to bend suddenly changed and resulted in FRP failure modes instead of concrete crushing [62]. In DBs, as the strength of concrete increases, the initial stiffness of the member also increases [92]. Further, if the beams strengthened with CFRP over the stirrups showed less ductile behavior compared to the other strengthened members, it was also observed that the overall load-carrying capacity of the beams does not necessarily increase with the increasing compressive strength of concrete [92,93,94]. In another study, it was found that by increasing the compressive strength of concrete, the initial stiffness was increased, but the total resistance of the beam and the post-peak stress absorption had no significant effect, and this mechanism also leads beams towards brittle failure. In addition, using CFRP between the stirrups gave higher strengthening efficiency than the over stirrups, even at lower compressive strengths; FRP failure or debonding failure appears instead of crushing failure [65]. A new type of reactive powder concrete having a 28-day compressive strength of about 83 MPa was also used to prepare DBs and was strengthened with externally bonded CFRP sheets. It was found that this technique successfully improved the overall performance of DBs with web openings, and their ultimate strength was increased by about 11 to 94% compared to the non-strengthened DBs with openings [53].

The DBs for flexural performance were prepared with different steel reinforcement ratios of 1.05, 1.53, and 2.09% and were strengthened with hybrid anchored FRP sheets. It was concluded that the higher steel ratio with the hybrid strengthening technique significantly increased the load-carrying capacity of the strengthened DBs, which was mainly due to the higher tensile strength of the steel bars. Further, the increase in steel ratio reduces the strengthening efficiency of the hybrid-strengthened beams, as a 1.05% steel ratio gave a strength efficiency of about 64%, a 1.53% ratio gave an efficiency of about 52%, and 2.09% steel gave a 29.3% strengthening efficiency. This reduction was mainly due to the lower load-carrying capacity of the control beam containing a higher steel ratio, so a further increase in steel ratio will be insignificant for the DBs [62]. Similarly, the DBs were also prepared with different lacing angles of 30, 45 and 60 degrees and steel ratios of 0.33, 1.13 and 2.01%. It was found that all the strengthened DBs showed only a 10% increase in the ultimate capacity, but the lacing angles significantly increased the stiffness by about 44% compared to the control beams. Furthermore, the beams with 45° lacings showed about 1.72 times the highest deflections at the ultimate loads compared to the reference beam [87].

### 5.3. Influence of Anchorages

Generally, in the strengthening of DBs, mostly EBRs like FRPs and steel plates were preferred to improve the flexural resistance of the members. However, the effectiveness of these techniques and materials can only be attained by the use of anchorages, because the anchorages prevent premature delamination failures of the beams. The DBs strengthened with CFRP sheets on both sides, and U-wrapping used as anchorages, showed an increase in ultimate load capacity by about 50%, initial stiffness by about 20%, and energy absorption by about 150% as compared to the control specimens. It was also found that the CFRP sheets used as U-shaped wrapping have shown significantly better performance in terms of ultimate capacity, ductility, and initial stiffness due to the proper anchoring effect [95,96]. The DBs were strengthened with CFRP strips, and in U-wraps, the mechanical anchor bolts (m6 × 50) and washers were used at the end to support the CFRP system. It was found that the CFRP systems with anchor bolts improved the ultimate strength by 9 to 15% compared to the same strengthened DBs without anchor bolts [53].

### 5.4. Influence of Shear-Span-to-Depth (a/d) Ratio

The shear-span-to-effective depth ratio (*a*/*d*) significantly impacts the behavior of strengthened DBs, particularly in how they resist loads and fail. In beams with a low *a*/*d* ratio (under 2.5), the load transfer is dominated by shear, leading to an arch-like action that enhances shear strength. In the majority of the cases, it was observed that as the *a*/*d* ratio increased, the ultimate load-carrying capacity of the DBs reduced due to higher tensile stresses in the mid-span [65]. Generally, it was found that the FRP-strengthened beams with lower *a*/*d* ratios exhibited higher shear capacity. Further, if the *a*/*d* ratio decreases, the strengthening efficiency increases, and no significant improvement in initial flexural cracking load was observed. In addition, it was found that at the *a*/*d* ratio of 0.85, the increase in ultimate load capacity was about 7 to 21%, whereas, as the ratio increased to 1.136, the increase in flexural capacity was found to be between 8 and 15.5%; however, both ratios have similar effects on the initial cracking loads [91]. Dirar et al. [97] investigated the shear-span-to-effective depth ratio of RC-strengthened T-beams and reported that the beams with an *a*/*d* ratio lower than 2.5 mostly failed in shear compression, while the beams with a higher value of *a*/*d* than 2.5 failed in diagonal tension.

### 5.5. Comparison of EBR Techniques Based on 1st Crack and Ultimate Loads

**Figure 4:**
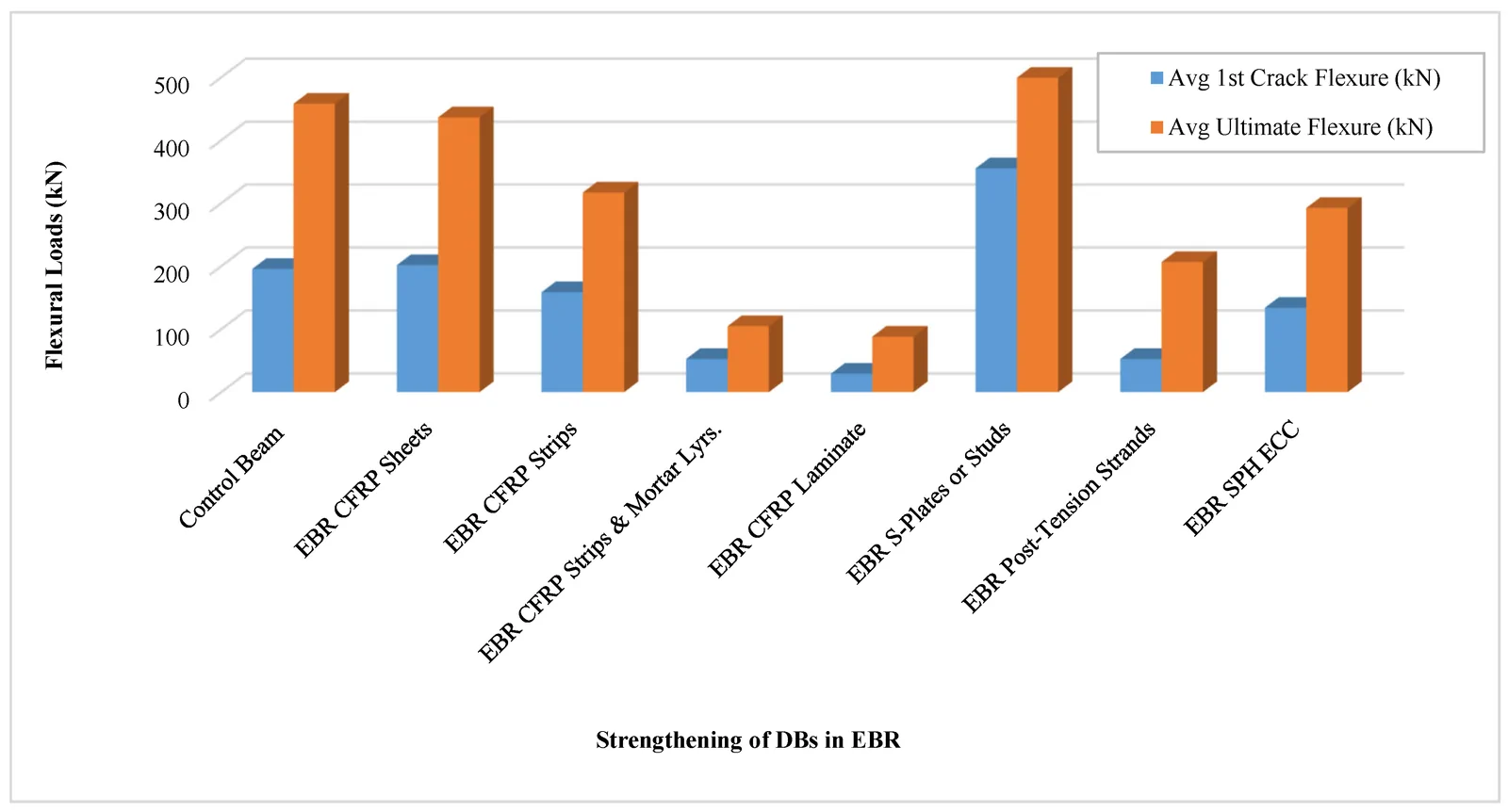
Relationship between 1st crack and ultimate flexural loads for control (solid beams without openings) and strengthened beams under different EBR techniques.

The strengthened DBs using EBR in different techniques were compared in terms of average ultimate flexural loads and 1st crack loads, as shown in Figure 4. This section discusses the techniques and their effects on ultimate flexural capacities, 1st crack loads, cost, ease of use, and potential downsides in the decision-making process for DBs.

#### 5.5.1. CFRPs in Different Techniques

The CFRP Sheets in EBR performed well among all the types of CFRP composites in terms of ultimate flexural strength; they showed an average ultimate flexural capacity of about 480 kN, which was around 140% higher, and the 1st crack load was about 115% higher as compared to the control deep beam. Although these sheets are highly effective, they can be costly and require precise application, and any possible errors in the process, including improper bonding at the interface, can significantly reduce their effectiveness [47,49,63,66,70,71,72]. Furthermore, the CFRP strips in EBR also improve the structural performance by increasing the flexural capacity by about 125% and the 1st cracking load by 100% in comparison to control unstrengthened DBs. The CFRP strips are slightly more versatile than CFRP sheets, especially in tricky areas, but their smaller surface area means careful placement is crucial for maximum effect. The CFRP strips were also used with a combination of mortar layers in EBR, and this combination improved the flexural performance by 75% and the 1st crack load by 70% in comparison to control DBs [51,69,73]. Although this combination gave decent results, it showed lower performance than CFRP sheets and strips alone. Among the CFRP composites, the least performance was observed in CFRP laminates in EBR techniques; they showed an average ultimate flexural load of 260 kN, which was about 30% higher, and the average 1st crack load was about 25% higher compared to control DBs. Although the CFRP laminates provide some benefits, they can only be preferred in situations where moderate strengthening is required [65,67,68].

#### 5.5.2. Other Materials in Different Techniques

The post-tensioned strands were also utilized in EBR as an alternative strengthening approach; they increased the average ultimate flexural capacity by about 110%, and the 1st crack load was around 90% higher compared to the control specimens. This technique is comparable to the CFRP sheets in strengthening effectiveness; however, it is more complex and requires careful attention during the execution of pre-tensioning and anchorage systems to avoid premature failures [81]. The SPH-ECC composites were also introduced for the strengthening of DBs in EBR; they showed on average 50 to 60% better performance in terms of flexural and 1st cracking load capacities. Although their structural performance was not better compared to other materials, SPH-ECC is known for better cracking resistance and durability, which help make the structures durable. The steel plates and studs in EBR strengthening showed only 10–15% better performance in terms of loading capacities, which makes this technique and material supplementary instead of a primary solution [62,86].

#### 5.5.3. Practical Insights of Different Materials in EBR

In terms of cost and performance, the CFRP sheets provide the most substantial performance boost with a 140% increase in ultimate flexure but are also among the most expensive options. In contrast, methods like S-plates or studs, which only offer a 10% increase, are more budget-friendly but deliver limited improvements. Finding the right balance between cost and the desired level of reinforcement is key. The ease of application of these techniques, like CFRP laminates or strips, which show a 30% to 125% improvement, is greater than that of more complex methods like post-tension strands (110% increase) or SPH ECC (50% increase), which require specialized skills. Ease of use is crucial, particularly in projects where time and resources are limited [1,2].

In terms of long-term durability, the effectiveness of these methods varies; for example, SPH ECC, with its 50% improvement, might not provide the highest strength increase but excels in crack resistance and durability. On the other hand, while CFRP methods offer up to a 140% increase, their longevity depends heavily on the quality of the adhesive used, which can be vulnerable to environmental factors. Overall structural behavior in flexural capacity is important; it is also essential to consider other aspects of the beam’s behavior, such as shear strength, deflection, and crack control. Some techniques, like CFRP sheets or post-tension strands, which offer significant strength gains, could alter the beam’s behavior in ways that need to be carefully managed to avoid issues like shear failure.

### 5.6. Comparison of NSM Techniques Based on 1st Crack and Ultimate Loads

**Figure 5:**
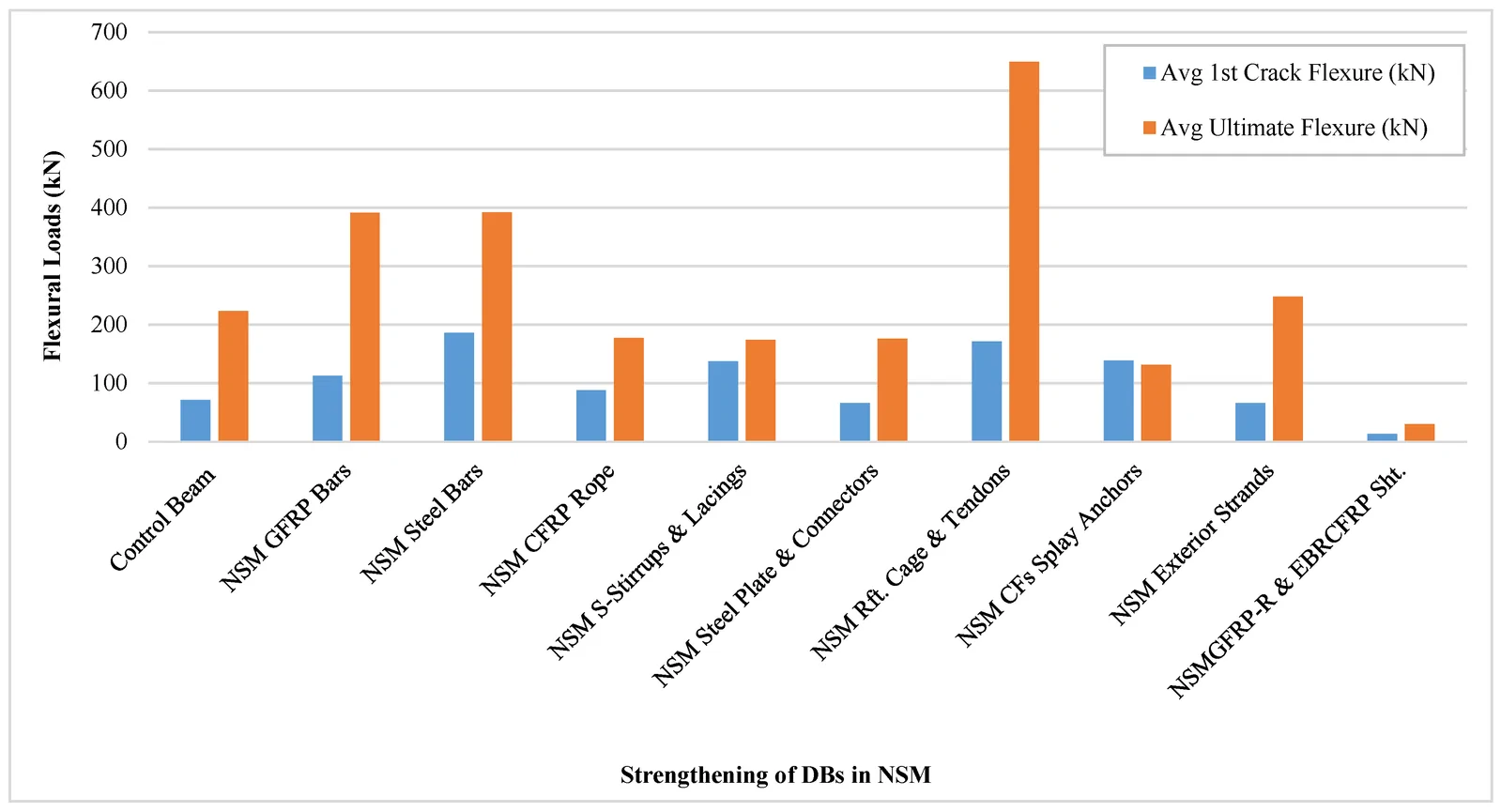
Relationship between 1st crack and ultimate flexural loads for control (solid beams without openings) and strengthened beams under different NSM techniques.

The strengthened DBs using NSM reinforcement in different techniques were compared in terms of two critical parameters, namely the average ultimate flexural loads and 1st crack loads, as shown in Figure 5. This section discusses the techniques and their effects on ultimate flexural capacities, 1st crack loads, cost, ease of use, and potential downsides in the decision-making process for DBs.

#### 5.6.1. Steel and GFRP Bars, and CFRP Ropes

The GFRP bars in NSM showed an average ultimate flexural capacity of about 300 kN, which was about 50% higher, and the 1st crack load was in the range of 80–150 kN, which was about 88% higher in comparison to control beams. This shows that GFRP bars are effective at both delaying the cracking loads and increasing the overall structural performance of the members [75,83,84]. Further, the steel bars in NSM showed even better performance than GFRP bars; the average ultimate load found was about 320 kN, which was about 60% higher than control beams, and the 1st crack flexural load was about 90% higher; therefore, it can be said that the use of high strength in NSM gives better performance and makes this technique most effective for strengthening DBs [87]. In addition, some studies [58,88] explored the use of CFRP bars or ropes in the NSM strengthening technique for deep beams. It was found that the CFRP rope used in NSM only showed 10% better results in ultimate capacity and 15% higher results in 1st crack loads in comparison to control beams. This performance was significantly lower than steel and GFRP bars, which was possibly due to different loading distributions or poor bonding with concrete.

#### 5.6.2. Other Materials in NSM Technique

Steel plate and connectors in the openings, when combined, showed a balanced performance; the ultimate flexural capacity was increased by about 25% and the 1st flexural crack load was increased by 35%. This method improves the structural efficiency in flexure without complications in applicability and can be considered a reliable method for moderate reinforcement in strengthened DBs [77]. Furthermore, the reinforcement cage and tendons performed extremely well in the NSM technique; they showed about 200% better flexural capacity, and the 1st crack load was about 150% higher compared to control beams. This technique is ideal for projects that require a substantial increase in strength, but it comes with higher costs and greater complexity [78]. The exterior strands provided a limited increase in flexural performance of about 10%, and they can be preferred only if minor strengthening reinforcement is required [98]. The carbon fibers with splay anchors were used in NSM for the flexural performance of DBs; it was found that the flexural performance and 1st crack load were increased by about 30% and 20%, respectively, compared to control DBs [67]. The NSM and EBR techniques were combined and used, considering GFRP rods in NSM and CFRP sheets in EBR. This composite technique showed the lowest performance in terms of flexural loading capacity and 1st crack loading; both results were only 5% higher than control beams. Therefore, it can be said that this combination of techniques may not be very effective for strengthening DBs, possibly due to limitations in the materials’ bonding or mechanical properties [84].

#### 5.6.3. Practical Consideration of Different Materials in NSM

In terms of cost and performance, the NSM reinforced with cage and tendons shows higher costs and greater complexity in comparison to techniques like NSM with CFRP rope and NSM with exterior strands, which, although they provide modest gains in ultimate structural loads, are likely easier and cost-effective for practical applications. In terms of ease of application, the NSM with CFRP bars and steel bars provide better performance and are easier to install in the strengthening system. The long-term durability performance can also be improved by using steel-based techniques like NSM Steel Bars and NSM Reinforced Cage and Tendons. In addition, CFRP-based materials offer higher strength gains, are safer against environmental challenges, and improve installation quality. Overall, the structural behavior of DBs strengthened in NSM using the cage and tendons technique showed significantly higher flexural capacity, shear strength, and ultimate deflections; therefore, careful design is essential to ensure that the enhanced strength does not introduce new structural challenges.

## 6. Stiffness/Energy Absorption of Strengthened DBs

**Figure 6:**
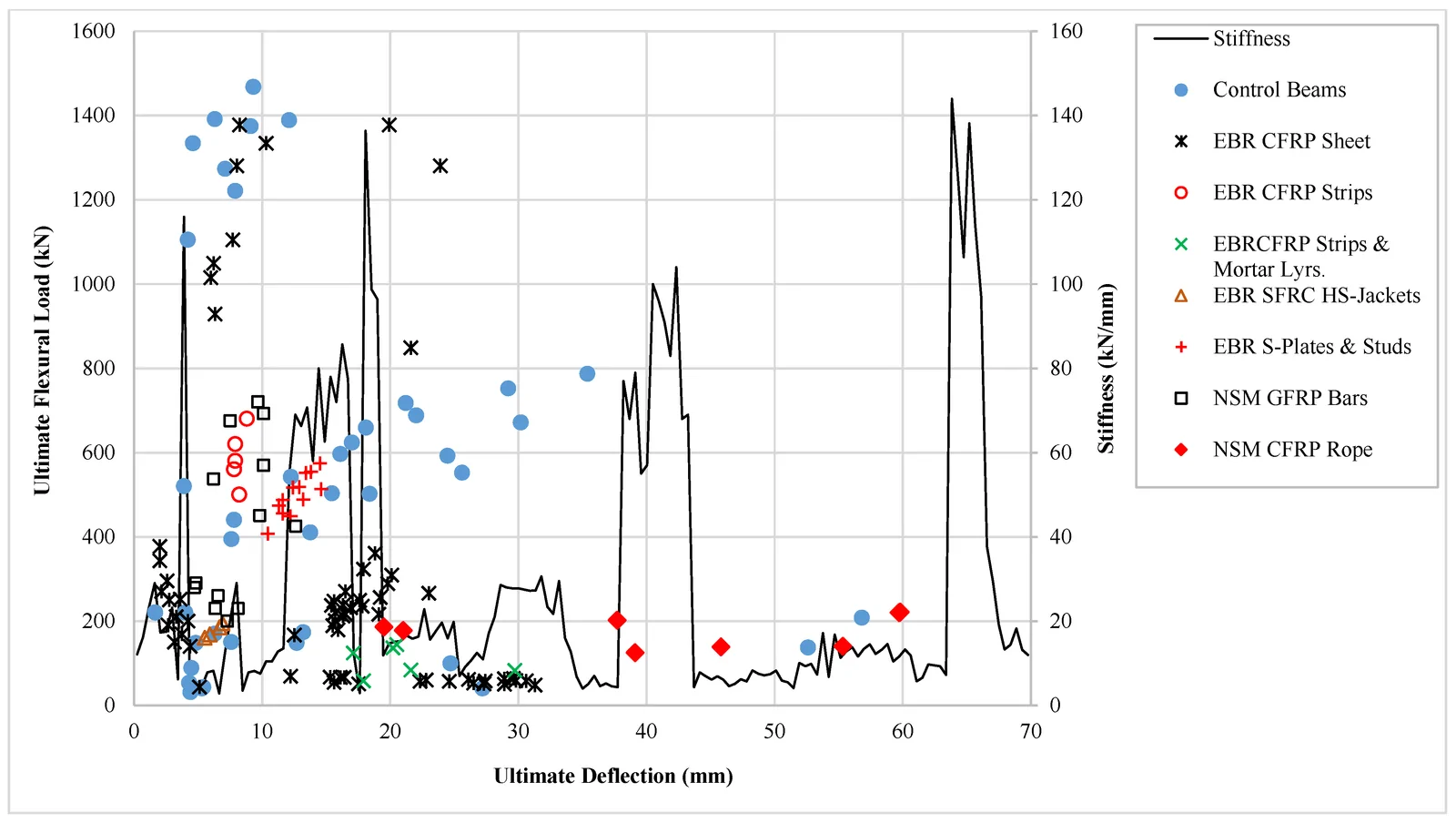
Relationship between ultimate load, ultimate deflection and stiffness of strengthened DBs in NSM and EBR techniques [1,25,44,49,62,63,71,74,79,80,83,88,99,100,101].

The relationship between ultimate loads, ultimate deflections, and stiffness of the unstrengthened and strengthened deep beams with different techniques is presented in Figure 6. Each point in the graph shows the ultimate flexural load and respective deflection of the beam, while the yellow line shows the stiffness at various deflection points. The control DBs showed the ultimate flexural loads in the range of 300 to 1300 kN, and the maximum deflections were found between 25 and 30 mm. Among these, most of the control DBs showed a maximum load of up to 550 kN, and only a few DBs of greater depth showed ultimate loads up to 1300 kN; therefore, for comparison with other techniques, only conventional control beam values were selected and maximum values were ignored. Most of the DBs showed an average stiffness of about 10 kN/mm, and in some beams, it reached 20 kN/mm, which shows that these beams carried significant loads and deflections, but their resistance to deformation was quite challenging. The DBs strengthened with CFRP sheets in EBR showed maximum load-carrying capacities in the range of 100 to 1380 kN (Figure 6), which were significantly higher in comparison to control DBs with an average ultimate capacity of 400 kN. Although the ultimate capacities of DBs using CFRP sheets were increased by about 50%, no significant changes were found in the deflections, which varied in the range of 4.5 to 31.3 mm. The interesting result of using this technique was that it significantly increased the stiffness of the DBs, which varied between 4 and 105 kN/mm, which was about 200% higher than control beams; therefore, it can be said that the rigidity of the beams was significantly increased [1,49,62,99].

The CFRP strips strengthened in EBR showed ultimate flexural capacities between 500 and 680 kN, which were on average higher than control beams with an average value of 400 kN. The deflections were about 40% lower, and the stiffness was almost similar to the control DBs. Further, the CFRP strips were also applied with mortar layers, and SFRC high-strength jackets were also used in the EBR technique. The ultimate flexural capacity of these strengthened beams was found to be in the range of 58 to 200 kN, which was on average 50% lower than that of control DBs. The deflection values were found in a wider range of 5–30 mm for both of these techniques, and such values were almost similar to control beams. Stiffness in these beams tended to increase moderately, with a range of around 9 to 22 kN/mm, showing a slight increase in stiffness, indicating that the combination of techniques provides a balanced enhancement in comparison to control DBs. However, it can be said that the mortar layers and SFRC jackets with CFRP strips likely contribute to the stiffness and overall rigidity of the beams, thereby reducing the risk of sudden failure. The steel plates and studs in EBR also improve the stiffness by about 25 kN/mm, but deflections were lower in comparison to control beams. NSM techniques using GFRP bars and CFRP ropes showed ultimate flexural capacities in the range of 120 to 700 kN, which were higher than control beams; however, the deflection achieved with these techniques was significantly improved, reaching up to 60 mm. The stiffness was low to moderate, with a wider range of 5 to 140 kN/mm and an average value of about 20 kN/mm for both techniques, representing a 100% increase in stiffness compared to control beams [25,63,71,100,101].

When comparing the stiffness of the various reinforcement techniques to the control beams, distinct differences emerge. The control beams (solid without openings) have the lowest stiffness, averaging around 10 kN/mm, which serves as the baseline. EBR CFRP sheets and EBR CFRP strips significantly enhance stiffness, with averages of around 30 kN/mm and 25 kN/mm, reflecting approximately 1.5 to 2 times the stiffness of the control beams. The combination of EBR CFRP Strips with Mortar Layers and EBR SFRC HS-Jackets achieves the highest stiffness, averaging about 40 kN/mm, making it the most effective in enhancing beam rigidity. EBR S-Plates and Studs and NSM GFRP Bars offer moderate stiffness improvements, averaging around 25 kN/mm and 20 kN/mm, translating to 150% and 100% increases over the control beams. NSM CFRP Rope shows the smallest increase in stiffness among the reinforcement techniques, averaging around 20 kN/mm, which still represents a 100% improvement. Overall, the combination techniques, particularly those involving EBR CFRP Strips with additional layers, provide the most substantial stiffness enhancements, while the NSM methods yield more moderate gains [44,74,79,80,83,88]. This increase in stiffness indicates that the additional reinforcement limits deflection at a given load by preventing fracture opening and preserving force transfer upon cracking. Further, comparisons between studies should consider the beam’s geometry, loading conditions, and stiffness-measuring technique, specifically whether stiffness represents the initial or post-cracking response.

## 7. Comparison of Strengthened DBs with and Without Openings

**Figure 7:**
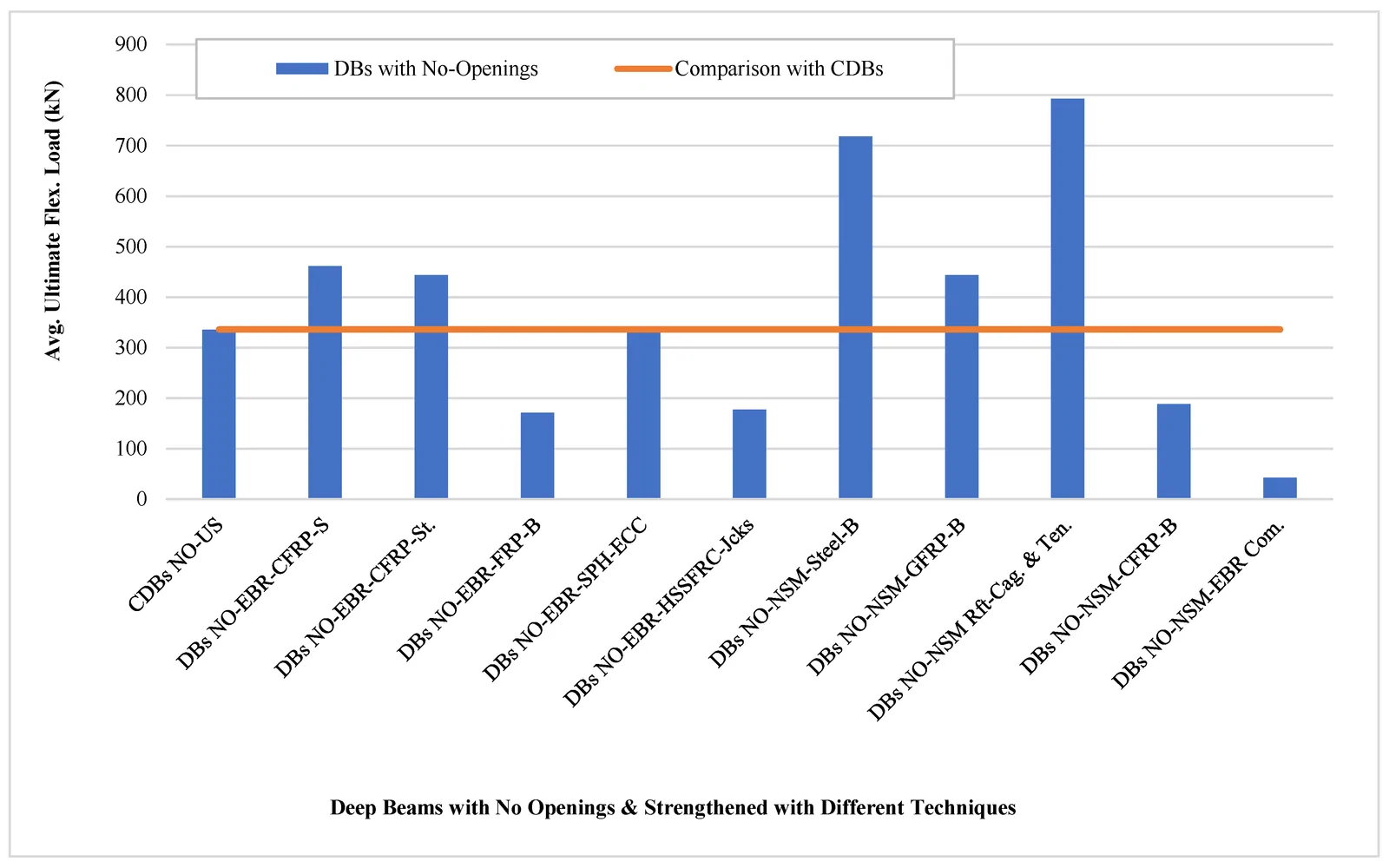
Average ultimate flexural loads of deep beams with no opening strengthened with different techniques [25,49,51,62,63,68,83,84,85,86,87,88,100,101].

Figure 7 presents the relationship between average ultimate flexural loads of solid deep beams (DBs) without openings, which were strengthened using different techniques. The control deep beams with no openings and unstrengthened (CDBs NO-US) showed an average ultimate capacity of 335 kN, which was considered a benchmark, and it was compared with other solid beams strengthened with different techniques. The beams strengthened with CFRP sheets and strips showed approximately 23% higher ultimate capacity of 400 kN in comparison to control beams. Although these techniques outperformed the control beams, the FRP bars in EBR showed about 48% lower results than CDBs, with an 171 kN ultimate capacity. Further, the EBR- SPH-ECC showed almost similar performance to the control beams, and FRC-jackets significantly underestimated the results by about 47%. On the other hand, the NSM techniques performed well considering steel bars and reinforcement cage and tendons; these techniques showed better ultimate capacities of about 53% and 58%, respectively, as compared to the control DBs. However, not all advanced techniques proved to be effective; for example, the combination of NSM and EBR methods, unexpectedly, led to a significant underperformance, with a flexural load capacity of about 85% lower than the control beams. This stark contrast points to potential issues with the compatibility or application of these combined methods, which may have resulted in premature failure or inadequate reinforcement.

**Figure 8:**
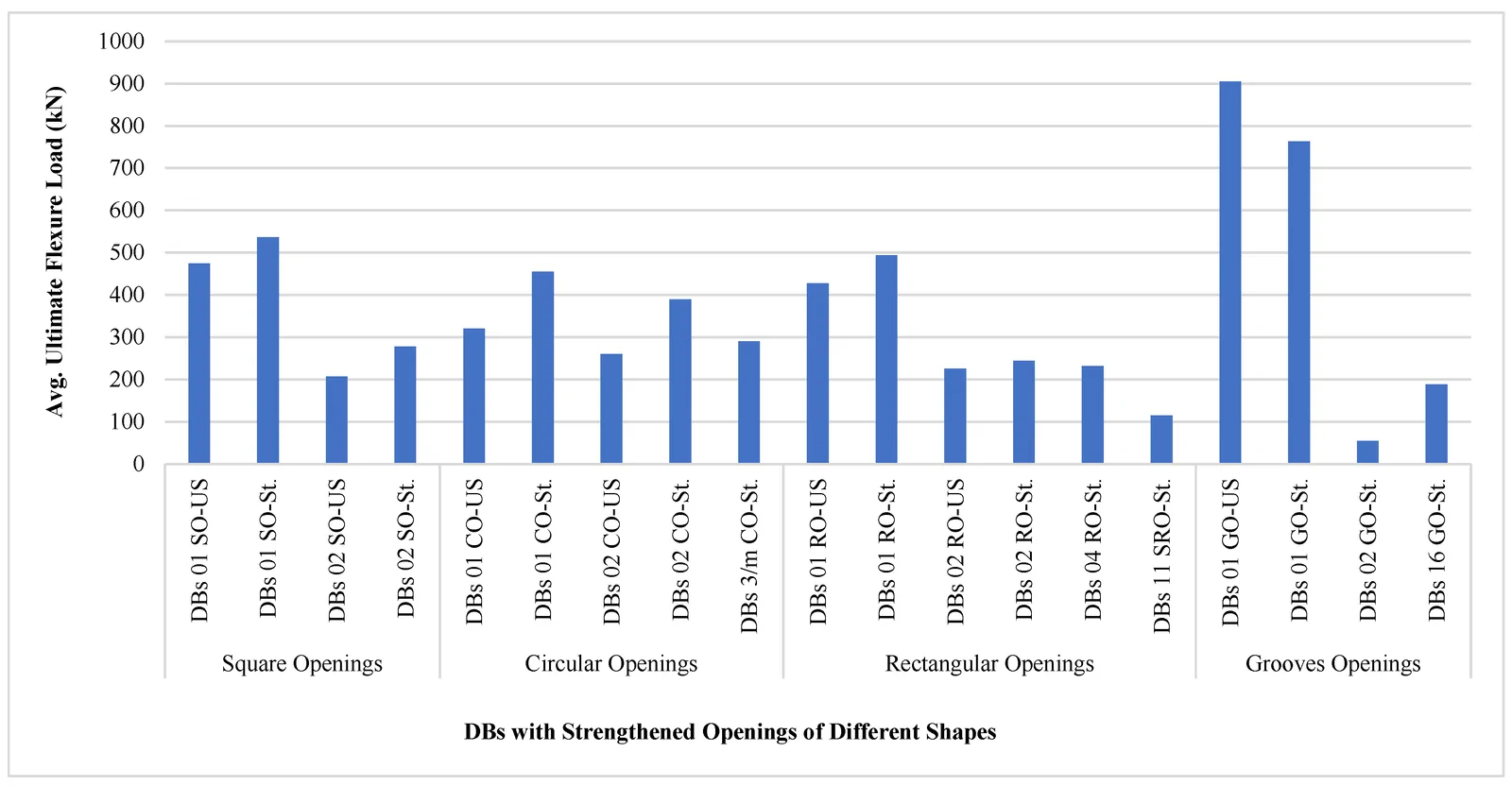
Different openings in deep beams strengthened with different techniques and materials [1,47,64,68,69,70,71,73,74,81].

Figure 8 describes the deep beams with openings; it was found that in some studies the openings were unstrengthened, and sometimes the openings in the beams were strengthened using different methods and techniques, so this section provides a detailed comparative analysis of DBs with strengthened and unstrengthened openings. The DBs mentioned in Figure 8 are (DBs 01 SO-US), which means the beam with one square opening, and that opening is unstrengthened. It was found that the unstrengthened DBs with one square opening showed an average ultimate flexural capacity of 470 kN; similar beams with one square opening were strengthened, and this improved the ultimate capacity by about 12%. Similarly, the DBs with two square openings and without strengthening significantly reduced the ultimate capacity in comparison to one square unstrengthened opening, with a reduction of about 56%, which shows that as the number of openings in DBs increases, the overall ultimate flexural capacity of the beams is significantly reduced. Furthermore, as the two square openings were strengthened, it increased the performance of beams from 207 kN to 278 kN [1,47,64,68,69,70,71,73,74,81].

Similarly, the DBs with one circular unstrengthened opening show an average ultimate capacity of about 320 kN, which significantly increased to 455 kN when the same opening was strengthened with a different technique. Similarly, when two circular openings were made, the unstrengthened average capacity dropped to 260 kN, which was about 19% and 26% lower than DBs with one circular opening and unstrengthened and strengthened members, respectively. The interesting fact is that when three or more circular openings in DBs were strengthened, they showed performance closer to the control DBs with one circular opening; the average difference found was about 10%. Furthermore, the DBs with one rectangular un-strengthened openings show an average ultimate capacity of about 428 kN, which was increased to 494 kN as the same opening was strengthened with a different technique. Similarly, when two rectangular openings were made, its un-strengthened average capacity dropped to 226 kN, which was about 47% and 54% lower than DBs with one rectangular opening and un-strengthened and strengthened members, respectively. The interesting fact is that when four rectangular openings in DBs were strengthened, they showed performance closer to the DBs with two rectangular openings, but as the openings increased up to 11, the average ultimate strength was drastically reduced by about 73% compared to the control unstrengthened DB with one rectangular opening. Groove openings in DBs presented very high results, highlighting how the effectiveness of strengthening techniques can differ dramatically depending on the opening shape. The highest flexural load recorded in the entire graph is associated with a groove opening that has been effectively reinforced, which suggests that, with the right approach, even complex shapes can be strengthened to perform exceptionally well [65,72,75,76,77,78].

The comparison of solid, with openings, and without-openings deep beams in terms of ultimate flexural capacity and the percentage increase or decrease relationship in respective techniques is shown in Table 1. The solid unstrengthened DBs were considered as reference beams; on average, the EBR-CFRP sheets showed about 37% higher flexural loads, and EBR-CFRP strips showed about 32% better results as compared to reference beams. Furthermore, the EBR-FRP bars underestimated the results by about 49%; HSSFRC jackets in EBR also showed 47% lower results, whereas SPH-ECC showed almost similar results to the reference beams. The NSM techniques using steel bars, GFRP bars, and reinforcement cage and tendons outperformed the EBR and significantly improved the performance of solid DBs by about 114%, 32%, and 136%, respectively, whereas the CFRP bars in NSM showed lower results by about 44% in comparison to reference beams. In addition, comparing strengthened beams with and without openings, based on percentage differences, shows that beams with openings usually experience reduced structural integrity. However, this impact varies considerably depending on the shape of the opening and the reinforcement method applied. Some reinforcement techniques can significantly enhance the load capacity of beams with openings, occasionally even outperforming beams without openings. This suggests that, for certain shapes like circular and square openings, the reinforcement can be highly effective. However, in other cases, particularly with more complex shapes like grooves and rectangles, the openings significantly improved the beam’s strength. However, if the number of openings increased, the ultimate strength diminished. These reductions emphasize that while certain techniques achieve some level of improvement, others fall short of fully restoring or exceeding the original load-bearing capacity. This highlights the necessity for carefully tailored reinforcement strategies that consider the specific geometry of openings [65,72,75,76,77,78].

## 8. Failure Modes and Cracking Behavior of Strengthened DBs

A total of 480 deep beams were tested under flexural loading; four beams were made of FRSCC, 20 were made of lightweight aggregate concrete, four were made of self-compacting concrete, five beams were made by SFC, and 447 beams were made of conventional concrete. All the deep beams showed ten different types of failure modes, including flexure, shear, flexure-shear, compression, shear compression, diagonal splitting failure, debonding and pull-out, concrete crushing, rupture, and sheet separation. The number of cracks presented in the subsequent section describes the observed cracking patterns and is not taken as a quantitative measure of ductility. More cracking may represent more widespread damage, but ductility requires a well-defined displacement- or curvature-based evaluation, while the energy absorption evaluation should be considered separately. If such measurements are unavailable, crack number alone is insufficient to determine whether ductility increased or decreased.

**Figure 9:**
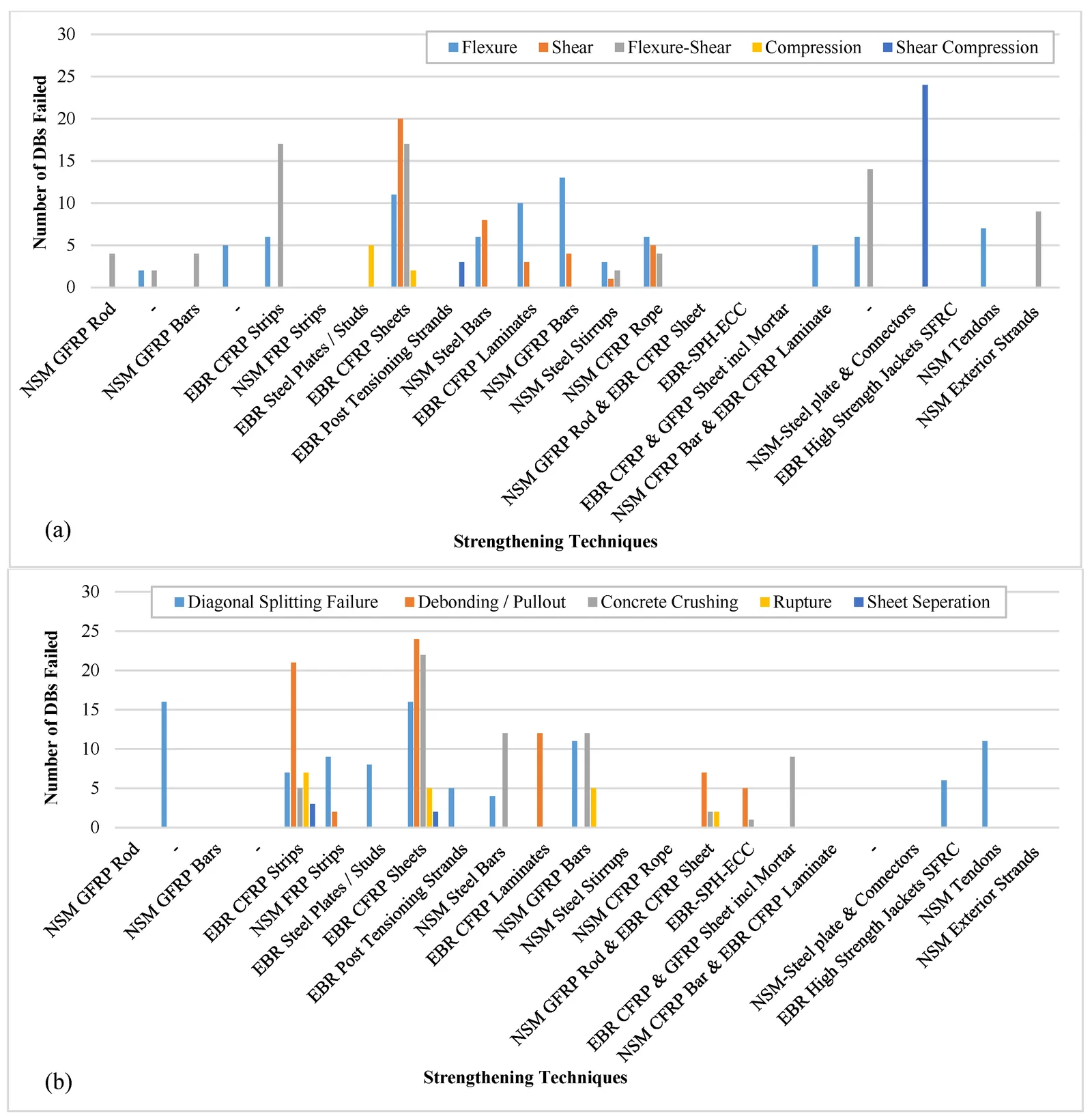
Flexural, shear, flexure-shear, compression, shear-compression, diagonal splitting failure, debonding/pullout, concrete crushing, rupture and sheet separation failure modes in deep beams [1,25,51,53,58,64,65,66,68,69,71,72,73,74,75,76,77,78,79,81,83,85,86,87,88,99,100,101,102,103].

Figure 9a,b shows ten different types of failure modes; the DBs strengthened with CFRP sheets in EBR techniques mostly showed load-carrying capacity in the range of 150 to 320 kN, and most beams failed in concrete cover separation; a few beams failed due to FRP sheet separation, and two beams failed due to rupture, which shows that in most cases the beam and FRP materials were not able to fully utilize their strength. Further, when two layers of CFRP sheets were used, the number of cracks observed at the time of failure ranged between 14 and 20; as the CFRP layers were increased to 3, a slight increase in ultimate capacity was observed; however, the total number of cracks increased to 28, which shows a change in the observed crack distribution in the beams [49]. In another study, the CFRP sheets were also used in one and two layers; it was found that the average ultimate capacity for control beams was up to 40 kN, which increased to 70 kN, and the major failure modes achieved were concrete crushing and intermediate debonding of the FRPs; 65% of beams failed due to debonding, and it can be considered as premature failure. In addition, the number of cracks in strengthened DBs was found to be in the range of 18 to 36, although it shows more cracks without improved ductility [62]. Similarly, the CFRP sheets used vertically increased the ultimate capacity by about 26%, and the beams showed flexural-shear and diagonal splitting failure modes, and the number of cracks increased from 14 to 20 [63]. CFRP sheets do not always perform well in EBR; 18 beams were prepared, among which 16 were strengthened with CFRP sheets with one and four layers. All the beams underestimated the ultimate strengths by a huge difference of about 55% compared to control DBs, and most of the strengthened beams showed shear, debonding, and rupture failure modes; further, the number of cracks increased from 15 to 21 in some beams [47,70]. The CFRP sheets and laminates increased the average ultimate capacity in the range of 10–60% and showed other types of failure modes, including flexural, diagonal splitting failure, flexural-shear, shear and concrete crushing, and immediate debonding of concrete [64,65,66,68,71,72,100,101].

The CFRP strips in EBR performed poorly in the DBs; the control DBs showed an average strength of about 400 kN, which was reduced to 270 kN using this strengthening technique. However, all the beams failed in shear and debonding failure modes, and the number of cracks was also lower in comparison to control beams, reduced from 28 to 22 [53,69]. In another study, the CFRP strips were used as U-shaped wrapping at 45°, with horizontal and vertical strips, and it performed well in comparison to previous studies; it improved the average ultimate capacity by about 12%, and most of the beams showed flexure, immediate debonding, and rupture failure modes. Further, in most of the beams, the number of cracks was similar to that of the control DBs, but in some specimens, a higher number of cracks was reported [25,51,73,102]. Furthermore, the DBs strengthened with steel plates and studs in EBR showed ultimate capacity in the range of 450 to 600 kN, and all the beams showed two types of failure modes, including diagonal splitting and compression. Furthermore, in control DBs, the failure cracks were about 7–9, which increased to 15 due to the application of steel plates and studs [1]. The new technique was introduced using NSM steel plates and connectors, but it drastically reduced the ultimate capacity of the beams by about 32%; decreased their ductility, and all the beams failed in shear-compression failure mode [77]. Instead of using steel plates and connectors, the use of high-strength steel jackets in EBR significantly increased the average capacity by about 50%, and all the beams showed diagonal tension failure [79]. In addition, the GFRP U-shaped wrapping performed amazingly and significantly increased the ultimate loads in comparison to control beams, and most beams showed the failure modes of shear cracks, debonding, and rupture; however, the cracks at the time of failure were very low, generally varying between 2 and 9 [99].

The conventional steel bars were replaced with GFRP bars as the main reinforcement in NSM; a total of three bars of diameters 8, 10, and 12 mm were used. Introducing GFRP bars reduced the overall strength of the members by about 12% compared to DBs with steel bars; further, the number of cracks was reduced from 24 to 15, and all the beams showed flexural failure mode [75,83,103]. The steel bars were used in grooves in the NSM technique at 30, 45, and 90 degrees; they increased the average ultimate load-carrying capacity of strengthened beams by about 10%. The major failure modes obtained were flexural, shear, shear and debonding, and flexural and cover separation. Further, the increase in the number of cracks was also higher compared to control DBs [85,87,103]. The CFRP ropes in NSM increased flexural capacity by about 24%, and failure modes were flexure and flexural-shear combined; however, no significant difference was found in terms of the number of cracks [58,75,88]. In addition, the composite techniques, including EBR-SPH-ECC, NSM-EBR combined with GFRP bars and CFRP sheets, and EBR CFRP mortar layers, increased the average ultimate capacity in the range of 10 to 43%, and all these techniques had crack counts similar to the control DBs, and the major failure modes achieved were rupture and debonding, flexural failure, and concrete crushing [74,76,84,86]. Furthermore, the latest technique of post-tensioned strands was also used in EBR; they reduced the ultimate loading capacity of the beams by about 22% and mainly failed in shear compression and diagonal splitting failure modes; further, the number of cracks reduced from 20 to 17 [78,81].

Recent studies indicate that the flexural capacity of an FRP-strengthened DB can be different from the ultimate load it can carry. The multi-strut model in [30] considers local, shear and flexural failure mechanisms and determines the ultimate capacity based on the most critical one. Yagmur [29] also demonstrated that thicker CFRP laminates partially recovered the performance of DBs with post-installed web openings that interrupted the reinforcement. Collectively, these investigations suggest that flexural resistance improvements should be evaluated alongside other potential failure mechanisms and the continuity of internal load-transfer paths. However, both studies are for beams with web openings, and the results should be used with care for solid DBs strengthened primarily in flexure.

The critical discussion on the flexural behavior of deep beams strengthened using different techniques showed complex performances in terms of higher load capacities, cracking patterns, and failure modes. In EBR, the commonly chosen materials are CFRP sheets, which generally increase the ultimate strengths but frequently lead to premature failures such as concrete cover separation or debonding, suggesting that the full potential of the materials was not realized. Furthermore, as the CFRP layers increased, the beams showed more cracks at failure, and this also changed the failure mode to rupture; however, a higher number of cracks does not establish higher ductility. Further, the steel studs and plates increased the ultimate loading capacity and faced more cracks at the time of failure, but the increase in ductility should not be considered based on the number of cracks alone. However, using similar methods in NSM made the beams weaker, highlighting how important the application methods are. GFRP bars, although reducing overall strength and ductility compared to steel, and composite methods, which provided moderate strength gains, had limited impact on ductility and often resulted in failure modes like debonding and flexural-shear failure. These findings indicate that while these techniques can enhance structural performance, their effectiveness is mixed, and they may introduce new challenges, particularly regarding crack formation and premature failures.

Based on the literature review, Table 2 summarizes the failure-mode map for strengthened deep beams, including key indicators, major controlling factors, and potential shifts in failure modes after strengthening. It can be concluded that the increased tensile resistance may increase the ultimate load-carrying capacity of the beams; however, the anchorage, compression struts, interfacial bonding, and diagonal crack resistance must sustain the resulting forces. Among these, the weakest component may fail first, leading to failure. Further, the failure-mode map shows the qualitative trends, and it indicates that the governing failure mainly relies on openings, shear-span-to-depth ratio, beam geometry, strengthening configuration, internal reinforcement, anchorage, and the bond quality.

## 9. Analytical Prediction Models of Flexural Strengths of SDBs

The analytical equations and models help engineers and designers to understand and forecast the behavior of strengthened DBs under various loading conditions and help to estimate ultimate loading capacities, deflections, failure mechanisms, stress–strain relationships, etc. Further, these mathematical empirical equations, formulations, data, and prediction models help engineers to solve complexities in the structures and offer insights that guide design, safety, and optimization assessments. Table 3 presents the prediction model for the ultimate bending moment capacity of reinforced beams strengthened with FRP composites as proposed by ACI-440-2R-17 [104], and CNR-DT-200 R1/2013 [105] is shown in Equation (1). Further, the depth of the neutral axis was estimated using different equations and trial-and-error methods, and factors *α* and *β* represents different set of strains in materials, further for the calculation of moment arms between FRP composites and steel bars, Equation (2) can be used. It was found that this model predicted the ultimate capacity of the beam with satisfactory accuracy in comparison to the experimental results. The ultimate moment of strengthened RC beams using FRCM composites can be predicted using the model proposed by ACI-549.4R-13 [106] in Equation (3) and Bencardino et al. [107] in Equation (4). It was found that the ACI equations (Equation (3)) gave better prediction results for debonding strains and flexural moments in comparison to another model (Equation (4)). The flexural capacity of the beam until the debonding of the FRP composites can also be predicted using the model shown in Equation (5). The end-debonding generally takes place due to higher strains in FRPs; therefore, after debonding, the loading capacity of beams significantly drops, and they behave like conventional control beams; therefore, this model can be used to predict the debonding flexural capacity of the RC beams [62]. The nominal flexural strength of conventional DBs can be predicted using the ACI 318M-14 [108] model, as shown in Equation (6), and this model estimated about 50–94% results for all the beams with a standard deviation of 79%, and this model can be considered acceptable and safe for laced-strengthened DBs. ACI-440-2R-08 [104] proposed a prediction equation for the flexural strength of DBs with a modification factor of 1.5 for strains, as presented in Equation (7), and it was found that the ACI-440 model for NSM bars can be used for NSM ropes with a modification factor. Alshamrani et al. [67] performed the seismic flexural behavior of CFRP-strengthened beams with fiber anchors and proposed trilinear moment–curvature analysis and prediction models as shown in Equation (8) for the yielding stage and Equation (9) for the rupture failure. The proposed prediction models for yielding strength and the rupture strengths showed that the difference between experimental and predicted results was in the range of 0–15% and 10–26%, respectively.

## 10. Special Innovative Techniques for DBs

The distinctive feature of these techniques is the way they adapt material placement and reinforcement detailing to the internal force paths of deep beams. The hybrid strengthening techniques use curved and arched sections containing reactive powdered concrete (RPC) in selected portions to support the compression paths and conventional or lightweight concrete in the rest of the specimen. Shakir et al. [80,109] investigated the innovative strengthening technique for precast hybrid DBs to propose an alternate design approach as compared to conventional DBs. A total of nine beams with dimensions 1.7 m span length, 180 mm width, and 450 mm depth were cast and tested under one-point and two-point static loadings. They followed three strategies during casting DBs; the first was the horizontal-hybrid mechanism in which half the section was made by conventional concrete (NWC/LWC) and the remaining half was made with reactive powder concrete (RPC). Secondly, the DBs were curved-hybrid, in which the inner curved portion was made of conventional concrete, and the outer portion of the beam was made with RPC. In the third strategy, the arched-hybrid was provided as a concrete ring in an arched shape at the inner portion, made with RPC, and the remaining beam was made by NWC/LWC. It was concluded that the curved model estimated good results for RPC and NWC under both loadings by about 5–12%, whereas this increase varied between 0 and 10% for RPC and LWC combinations under both loadings. However, the increase in loading capacity for arched beams ranged between 13 and 20% under one-and two-point loadings. Furthermore, it was also found that it is possible to use low-strength concrete or lightweight concrete to reduce the dead load of the deep beams. In terms of failure modes under two-point loading, the diagonal shear failure was transformed into flexural failure in arched-model hybrid beams.

Al-Mahbashi et al. [110] explored the two-span continuous deep beams using high-strength concrete and dimensions of 150 mm width and 400 mm depth, and each span length was 1.120 m with circular and rectangular openings of the same cross-sectional area. They reported that instead of higher-strength concrete, the openings in the DBs reduced the failure load capacity of the beams by about 38%. Yang et al. [111] also investigated the effect of high-strength concrete on the behavior of deep beams. It was concluded that at the initial loading stages, the dimensions, including width and depth, have no significant effect on the mid-span deflection, but it significantly increases deflection after the emergence of diagonal cracks. Further, the rigidity of the beams was not significantly affected by increasing the strength of concrete due to the web openings, but the maximum crack width was reduced, which might be due to the higher strength of concrete, opening size, and shear-span-to-depth ratio. Farouk et al. [77] proposed an innovative technique for deep beams to evaluate the structural performance and prepared 20 beams, of which 11 contain various openings, and some beams were strengthened using different techniques. The special type of steel plates and connectors was designed and placed around the openings to further improve the structural performance, cracking behavior, and failure modes. The strengthening techniques were designed considering the optimal strut-and-tie model, which was not only limited to local strengthening around openings but also reinforced the load paths and trajectories around the openings. Whereas, in the second technique, the additional stirrups were provided as struts to counteract the lateral tension in the members. It was concluded that the DBs with openings placed around the loading path carried higher loads and also increased the stiffness in comparison to members with interrupted openings and load paths, despite shear-span lengths. It was also found that the DBs with rectangular openings and strengthened with external fasteners showed the best results, and strengthening efficiency was significantly higher in comparison to other DBs.

## 11. Discussion

The reviewed research suggests that the performance of strengthened deep beams depends on the compression paths. For EBR CFRP sheets and strips, the load capacity was generally enhanced, but the reinforcement could not always achieve its full tensile resistance due to debonding and separation of the concrete cover. Thus, it was not always the case that adding layers increased the strength. Anchorage can aid in preserving force transfer, but its value is dependent on the resistance of the surrounding concrete and whether another failure mechanism takes over. NSM reinforcement, integrated NSM-EBR systems, and jackets strengthen beams in different ways. The efficiency depends on the reinforcement orientation, the groove dimensions, the anchorage, and the interaction with the existing steel. The crossing of the main diagonal cracks by bars or strips can be more effective in limiting crack opening. The improvements achieved for jackets and hybrid systems are also attributed to the modification in the beam section and the internal force transfer. So, these advantages should be seen as a consequence of the overall strengthening system, not just of the increased material. Openings complicate this behavior by disrupting the paths of concrete compression. Their influence varies with their size, shape, number, and position relative to the loads and supports. While strengthening may recover some capacity lost by an opening, it will not restore the performance of an intact beam. Therefore, it is essential to distinguish the comparisons with solid control DBs from the comparisons with unstrengthened beams having the same openings. Apart from the load capacity, the stiffness and the deformation capacity have to be investigated independently. A beam with a higher ultimate load does not necessarily mean it is more ductile or that it may absorb more energy. Variations in beam configuration, concrete strength, reinforcement ratio, loading, and control specimens further limit comparability among investigations. The results thus support selection of a strengthening configuration that emphasizes the critical weakness of the beam, rather than considering one strengthening technique as the best choice for all structural applications.

## 12. Conclusions and Future Recommendations

This study provides a thorough review and investigation of the flexural performance of deep beams strengthened with internal and external reinforcements using steel, FRP, and hybrid techniques. It presents a comprehensive database that includes different materials, techniques, schemes, layers, and anchorage systems, and discusses their effects on ultimate flexural loads, stiffness, and failure modes. The following conclusions are drawn:

(1) The strengthening of deep beams using EBR and NSM can increase the load capacity, but the increase depends on the reinforcement detailing, anchorage, bond quality, and the condition of the original beam. No material or strengthening technique consistently outperformed the other materials or techniques in the reviewed literature.
(2) Premature debonding and concrete-cover separation may prevent externally bonded reinforcement from achieving its maximum capacity. But an increase in the number of layers or the bonded length does not necessarily mean providing higher strength; the resistance of the surrounding concrete and adequate anchorage are equally crucial.
(3) The reinforcement orientation and location significantly affect deep-beam behavior. The reinforcement configuration restrains major diagonal cracks and supports internal force transfer, which increases structural performance and, in some cases, transforms failure into flexural behavior.
(4) The openings in deep beams can decrease structural capacity by disrupting the compression paths. The effects depend on the location and geometry of the opening. Therefore, the strengthening should be evaluated against solid control and unstrengthened beams with openings for comparable results.
(5) The hybrid strengthening technique and high-strength jacket techniques in the EBR method have proven to be highly effective methods for strengthening DBs in flexural performance. Hybrid strengthening approaches, such as those incorporating lower-strength concrete at the bottom, high-strength concrete at the top, and two struts inclined at a 45° angle, have demonstrated significant improvements in the ultimate load and deflection.
(6) The insufficient interfacial bonding length in the strengthened beams causes premature debonding failure. The location, spacing, and diameter of bars in NSM significantly improve the ultimate strength of the member, especially when they are provided against the diagonal crack path.
(7) Anchorage such as U-wrap and mechanical anchors significantly improves the structural performance of beams; the CFRP sheets with U-wrap significantly increased the ultimate capacity, ductility, and initial stiffness due to the proper anchoring effect.

For future investigations, EBR, NSM, and hybrid strengthening should be compared under matched conditions, such as similar beam geometry, internal reinforcement, concrete strength, and the loading arrangements. For beams with openings, testing must include a control specimen with a solid web and an unstrengthened specimen with the same opening. This would help identify recovery of lost capacity from performance relative to an undamaged beam. Further research is needed to clarify the connection between the bonded length, stiffness of reinforcement, dimensions of grooves, and anchorage. Measurements of interface slip, reinforcement strain, and crack formation would help explain why extra reinforcement improves some beams but provides limited benefit in others. Measurements could help determine when failure changes from debonding to concrete-cover separation or compression crushing. The strengthening of openings needs to be investigated with particular attention to the tensile forces around them and the compression loading paths. Further research is required for DBs against environmental exposure, fatigue, sustained and cyclic loading, and the long-term performance of adhesives and the anchorage systems. The limitations of this study are the variability in the control specimens, testing conditions, and strengthening techniques; therefore, the percentage increase in the structural responses must be interpreted based on these variations.

## Figures and Tables

**Table 1:** Percentage increase or decrease in ultimate flexural loads in strengthening DBs and strengthening of openings in DBs [1,44,47,58,64,65,66,67,68,69,70,71,72,73,74,75,76,77,78,79,80,81,102,103].

Beams	Strengthened DBs VS. Strengthened Openings in DBs	Average Ultimate Flexural Load (kN)
CDBs NO-US	0%	335.8
DBs NO-EBR-CFRP-S	37%	461.6
DBs NO-EBR-CFRP-St.	32%	444.2
DBs NO-EBR-FRP-B	−49%	171.2
DBs NO-EBR-SPH-ECC	−1%	332.0
DBs NO-EBR-HSSFRC-Jackets	−47%	177.0
DBs NO-NSM-Steel-B	114%	718.5
DBs NO-NSM-GFRP-B	32%	443.5
DBs NO-NSM Reinforcement-Cag. & Ten.	136%	792.8
DBs NO-NSM-CFRP-B	−44%	188.7
DBs NO-NSM-EBR Com.	−87%	42.4
DBs 01 SO-US	41%	474.3
DBs 01 SO-St.	60%	536.5
DBs 02 SO-US	−38%	207.2
DBs 02 SO-St.	−17%	278.0
DBs 01 CO-US	−5%	320.3
DBs 01 CO-St.	36%	455.4
DBs 02 CO-US	−23%	259.9
DBs 02 CO-St.	16%	389.7
DBs 3/m CO-St.	−14%	290.0
DBs 01 RO-US	27%	428.1
DBs 01 RO-St.	47%	493.6
DBs 02 RO-US	−33%	225.5
DBs 02 RO-St.	−27%	244.4
DBs 04 RO-St.	−31%	232.3
DBs 11 SRO-St.	−66%	114.9
DBs 01 GO-US	170%	905.3
DBs 01 GO-St.	127%	763.0
DBs 02 GO-St.	−84%	54.4
DBs 16 GO-St.	−44%	188.8

**Table 2:** Failure-mode map considering main indicators and factors for strengthened deep beams.

Failure Modes	Main Indicators	Major Controlling Factors	Possible Failure Shift After Strengthening
Flexural Failure	Flexural compression crushing; steel yielding	Compression-zone resistance; longitudinal steel	Bond, shear, or anchorage failure
Strut Crushing	Diagonal concrete crushing	Opening location; Strut strength and width	Limited strength gain from tensile reinforcement
Diagonal Splitting or Tension	Strut splitting; Diagonal crack propagation	Openings; web reinforcement; crack crossing reinforcement	Strut crushing or flexural failure
FRP Debonding	Premature FRP-end debonding	Bonded length; anchorage; FRP-end stresses	Cover separation; debonding
Concrete Cover	Cover detachment	Internal steel arrangement; concrete cover; cracking	Premature failure
NSM Bond Failure	Groove cracking; cover separation; reinforcement slippage	Filler; groove dimensions; development length	Compression failure; rupture; yielding
Anchorage Failure	Concrete crushing; anchor rupture; pull-out	Bond with surrounding concrete; anchor capacity	Strut, bond or reinforcement failure
FRP Rupture	Fiber fracture	Anchorage and bond strength; FRP strain	Brittle tensile failure

Note: This table summarizes potential failure mechanisms rather than providing numerical capacity predictions; the ultimate load depends on the governing failure mechanism, and premature bond, anchorage, or shear failure may prevent the flexural capacity from being reached.

**Table 3:** Prediction models for ultimate moments for strengthened DBs with FRPs, FRCM composites.

Source	Ultimate Bending Moment or Load Equations	Guidelines	Equations No.
ACI-440-2R-17 [104]	$M_{n}=A_{s}f_{s}\left(d-\frac{\beta_{1}c}{2}\right)+\psi_{f}A_{f}f_{\mathrm{fe}}\left(d_{f}-\frac{\beta_{1}c}{2}\right)+A_{s}^{\prime }f_{s}^{\prime }\left(\frac{\beta_{1}c}{2}-d^{\prime }\right)$	RC beams strengthened with FRP composites	(1)
ACI-440-2R-17 [104]	*M_n_* = 0.9*A_s_f_s_d* + *A_f_E_f_ε_fd_*(0.9*d* + c^*^)	For moment arms of steel bars and FRPs, ‘c’ is the distance b/w FRP sheets and tensile bars.	(2)
ACI-549-4R-13 [106]	$M_{n}=A_{s}f_{s}\left(d-\frac{\beta_{1}c}{2}\right)+A_{f}f_{\mathrm{fe}}\left(d_{f}-\frac{\beta_{1}c}{2}\right)+A_{s}^{\prime }f_{s}^{\prime }\left(\frac{\beta_{1}c}{2}-d^{\prime }\right)$	Ultimate flexural bending moment for FRCM-strengthened reinforced concrete beams.	(3)
Bencardino et al. [107]	*M_n_* = 0.9*dA_s_f_s_* + 0.9*A_f_E_f_ε_deb_*		(4)
Wang et al. [62]	*M_n_* = *A_s_*{*f_y_* + [*E_s_*_,2_(*ε_s_* − *ε_s_*_,*y*_)]}(*d_s_* − *x*_0_ − *y_c_*_,*t*_) + *A_f_E_f_ε_f_*_,*d*_(*d_f_* − *x*_0_ − *y_c_*_,*t*_)	The flexural capacity of beams until debonding of the FRP composites.	(5)
ACI-318M-14 [108]	$M_{n}=\rho_{f}f_{y}bd^{2}\left(1-0.59\rho_{f}\frac{f_{y}}{f_{c}^{\prime }}\right)$	Flexural strength of control DBs with stirrup angles.	(6)
ACI-440-2R-09 [104]	*M_n_* = *M_ns_* + *M_nf_P_flex_*_._ $M_{\mathrm{ns}}=A_{s}f_{s}\left(d-\frac{\beta_{1}C}{2}\right)$ $M_{\mathrm{nf}}=A_{f}f_{\mathrm{fe}}\left(d_{f}-\frac{\beta_{1}C}{2}\right)$	Contribution of nominal steel and FRP reinforcement to the flexural strength of beams.	(7)
Alshamrani et al. [67]	$M_{y}=A_{s}f_{y}\left(d-\left(0.5\hat{\beta_{1}}c_{y}\right)\right)+A_{f}E_{f}\varepsilon_{\mathrm{fe}}\left(d_{f}-\left(0.5\hat{\beta_{1}}c_{y}\right)\right)+A_{s}^{\prime }E_{s}^{\prime }\varepsilon_{s}^{\prime }\left(0.5\hat{\beta_{1}}c_{y}-d^{\prime }\right)$	For the prediction of the yielding load in the beam.	(8)
	$M_{\mathrm{nr}}=A_{s}f_{y}\left(d-\left(0.5\hat{\beta_{1}}c_{\mathrm{nr}}\right)\right)+A_{f}E_{f}\varepsilon_{\mathrm{fu}}\left(d_{f}-\left(0.5\hat{\beta_{1}}c_{\mathrm{nr}}\right)\right)+A_{s}^{\prime }E_{s}^{\prime }\varepsilon_{s}^{\prime }\left(0.5\hat{\beta_{1}}c_{\mathrm{nr}}-d^{\prime }\right)$	For the prediction of rupture failure/ultimate moment capacity of the beam.	(9)

Note: *M_n_* is the nominal flexural moment, *A_s_* represents the cross-sectional area of tension steel, $A_{s}^{\prime }$ is the cross-sectional area of compression steel reinforcement, *A_f_* represents the cross-sectional area of FRP reinforcement, *f_s_* shows the stress in tension steel, $f_{s}^{\prime }$ is the stress in compression steel, *f_y_* represents the yield strength of steel reinforcement, $f_{c}^{\prime }$ shows the concrete compressive strength, *f_fe_* is the effective tensile stress in strengthening reinforcement, *E_f_* represents the tensile elastic modulus of FRP, *d* is effective depth (distance from extreme compression fiber to centroid of tension steel, *d*′ is the effective depth for compression steel (distance from the extreme compression fiber to the centroid of compression steel), *d_f_* is the distance from the extreme compression fiber to the centroid of the strengthening reinforcement, *b* is the width of the rectangular beam, *c* is the neutral-axis depth measured from the extreme compression fiber.

## Data Availability

No new data were created or analyzed in this study. Data sharing is not applicable to this article.

## Nomenclature

The following abbreviations are used in this manuscript:

DBs	Deep Beams
NSM	Near-Surface Mounted
NSE	Near-Surface Embedment
EBR	Externally Bonded Reinforcement
EB	Externally Bonded
RC	Reinforced Concrete
RCDBs	Reinforced Concrete Deep Beams
FRP	Fiber-Reinforced Polymers
CFRP	Carbon Fiber-Reinforced Polymer
GFRP	Glass Fiber-Reinforced Polymer
AFRP	Aramid Fiber-Reinforced Polymer
BFRP	Basalt Fiber-Reinforced Polymer
RCC	Reinforced Cement Concrete
NWC	Normal Weight Concrete
LWC	Lightweight Concrete
SCC	Self-Compacting Concrete
FRC	Fiber-Reinforced Concrete
SFC	Steel Fiber Concrete
SFRC	Steel Fiber-Reinforced Concrete
FRSCC	Fiber-Reinforced Self-Compacting Concrete
RPC	Reactive Powder Concrete
SPHECC	Steel and Polyvinyl-alcohol Hybrid Fiber-Reinforced Engineered Cementitious Composite
FRCM	Fiber-Reinforced Cementitious Matrix
CDBs	Control Deep Beams
NO	No Openings
US	Unstrengthened
CDBs-NO-US	Control Deep Beams—No openings and Unstrengthened

## Appendix A. Research Progress in Flexural Behavior of Reinforced Concrete Deep Beams

**Beam Specifications**			**Concrete Prop.**	**Reinforcement Details**			**Strengthening Techniques**				**Openings**			**Loading Conditions**					**Failure Modes**	**Refs.**
**Beam’s Names/Type of Concrete**	**L** **(m)**	**X-Section (mm)**	$f_{c}^{’}/f_{t}$ **(MPa)**	**No Steel Bars** **(x × y)**	**Diameter of Steel** **(mm)**	**Location of Reinforcement**	**Technique/Material**	**Layers (Nos.)**	**Thickness of Bars/Layer (mm)**	**Location of Material Placed**	**Shape**	**Dimensions** **(mm)**	**Strengthening Material**	**1st Crack Flexure (kN)**	**Ult. Flexure Load** **(kN)**	**Ult. Deflections (mm)**	**Stiffness** **(kN/mm)**	**Total No. of Cracks**		
B1/NC B2/NC B3/NC B4/NC B5/NC B6/NC B7/NC B8/NC B9/NC B10/NC B11/NC B12/NC B13/NC B14/NC	2.2 2.2 2.2 2.2 2.2 2.2 2.2 2.2 2.2 2.2 2.2 2.2 2.2 2.2	250 × 300 250 × 300 250 × 300 250 × 300 250 × 300 250 × 300 250 × 300 250 × 300 250 × 300 250 × 300 250 × 300 250 × 300 250 × 300	25.0/3.7 25.0/3.7 25.0/3.7 25.0/3.7 25.0/3.7 25.0/3.7 25.0/3.7 25.0/3.7 25.0/3.7 25.0/3.7 25.0/3.7 25.0/3.7 25.0/3.7 25.0/3.7	2 × 2 2 × 2 2 × 2 2 × 5 2 × 5 2 × 5 2 × 2 2 × 2 2 × 5 2 × 5 2 × 2 2 × 2 2 × 5	D12C + D16T D12C + D16T D12C + D16T D12C + D10T D12C + D10T D12C + D10T D12C + D22T D12C + D22T D12C + D14T D12C + D14T D12C + D28T D12C + D28T D12C + D18T D12C + D18T	Y-R8@120c/c Y-R8@120c/c Y-R8@120c/c Y-R8@120c/c Y-R8@120c/c Y-R8@120c/c Y-R8@100c/c Y-R8@100c/c Y-R8@100c/c Y-R8@100c/c Y-R10@80c/c Y-R10@80c/c Y-R10@80c/c Y-R10@80c/c	EBR-CFRP Sheet EBR-CFRP Sheet EBR-CFRP Sheet EBR-CFRP Sheet EBR-CFRP Sheet EBR-CFRP Sheet EBR-CFRP Sheet	2 2 2 2 2 2 3 3 3 3 3 3 3 3	20 20 20 20 20 20 30 30 30 30 30 30 30 30	Bottom	No Opening provided.			41 63 62 41 65 72 50 70 46 70 57 52 98 96	148 167 201 179 189 216 238 247 266 270 309 361 289 323	12.1 11.9 14.7 15.1 15.3 16.6 15.8 16.3 22.8 15.6 17.7 19.6 15.9 19.8	12.1 11.9 14.7 15.1 15.3 16.6 15.8 16.3 22.8 15.6 17.7 19.6 15.9 19.8	14 16 19 17 19 20 23 25 22 27 28 27 22 24	Concrete Separation Concrete Separation Concrete Separation FRP Sheet Separation Concrete Separation Concrete Separation FRP Sheet Separation Concrete Separation Concrete Separation Concrete Separation FRP Sheet Separation FRCM Mesh Rupture Concrete Separation FRCM Mesh Rupture	[49]
REF/NC SQ/NC SQ.P/NC SQ.P.N/NC CR/NC CR.P/NC CR.P.N/NC HR/NC HR.P/NC HR.P.N/NC VR/NC VR.P/NC VR.P.N/NC	1.0 1.0 1.0 1.0 1.0 1.0 1.0 1.0 1.0 1.0 1.0 1.0 1.0	100 × 400 100 × 400 100 × 400 100 × 400 100 × 400 100 × 400 100 × 400 100 × 400 100 × 400 100 × 400 100 × 400 100 × 400 100 × 400	40.0/4.1 40.0/4.1 40.0/4.1 40.0/4.1 40.0/4.1 40.0/4.1 40.0/4.1 40.0/4.1 40.0/4.1 40.0/4.1 40.0/4.1 40.0/4.1 40.0/4.1	6 × 2 6 × 2 6 × 2 6 × 2 6 × 2 6 × 2 6 × 2 6 × 2 6 × 2 6 × 2 6 × 2 6 × 2	D4C + D16T D4C + D16T D4C + D16T D4C + D16T D4C + D16T D4C + D16T D4C + D16T D4C + D16T D4C + D16T D4C + D16T D4C + D16T D4C + D16T D4C + D16T	Y-R4@88c/c Y-R4@88c/c Y-R4@88c/c Y-R4@88c/c Y-R4@88c/c Y-R4@88c/c Y-R4@88c/c Y-R4@88c/c Y-R4@88c/c Y-R4@88c/c Y-R4@88c/c Y-R4@88c/c Y-R4@88c/c	- - EBR-Steel Plates/Studs - EBR-Steel Plates/Studs - EBR-Steel Plates/Studs - EBR-Steel Plates/Studs	- - 2 2, 2 - 2 2, 2 - 2 2, 2 - 2 2, 2	Steel Plates = 6 mm Steel Studs = 8 mm	- Center Center-Bottom - Center Center-Bottom - Center Center-Bottom	- Square Square Square Circle Circle Circle Rectangle Rectangle Rectangle Rectangle Rectangle Rectangle	- 45 × 45 45 × 45 45 × 45 Ø—50.8 Ø—50.8 Ø—50.8 58 × 35 58 × 35 58 × 35 35 × 58 35 × 58 35 × 58	- - 6 Steel Plates 6 Steel Plates and 8 Steel Studs	180 337 363 388 351 386 396 314 382 365 305 320 357	596 474 519 554 488 552 574 449 488 514 407 456 517	16.1 11.3 12.8 13.8 11.6 13.4 14.5 12.2 13.2 14.6 10.4 11.6 13.3	- - - - - - - - - - - - -	9 13 9 12 11 7 12 13 11 15 12 11 8	Diagonal Splitting Diagonal Splitting Diagonal Compression Diagonal Compression Diagonal Splitting Diagonal Splitting Diagonal Splitting Diagonal Compression Diagonal Splitting Diagonal Compression Diagonal Compression Diagonal Splitting Diagonal Splitting	[1]
NR/NC NR-R1/NC NR-R2/NC NR-30/NC NR-50/NC N1B7/NC N1B9/NC N1B11/NC N1B14/NC N2B9+14/NC N1H7/NC N1H8/NC N1H9/NC N1H10/NC N1H11/NC N1H12/NC N1H13/NC N1H14/NC N2H14+9/NC N1H9-R1/NC N1H9-R2/NC N1H9-30/NC N1H9-50/NC N1H9-3/NC N1H9-5/NC	1.8 1.8 1.8 1.8 1.8 1.8 1.8 1.8 1.8 1.8 1.8 1.8 1.8 1.8 1.8 1.8 1.8 1.8 1.8 1.8 1.8 1.8 1.8 1.8 1.8	100 × 180 100 × 180 100 × 180 100 × 180 100 × 180 100 × 180 100 × 180 100 × 180 100 × 180 100 × 180 100 × 180 100 × 180 100 × 180 100 × 180 100 × 180 100 × 180 100 × 180 100 × 180 100 × 180 100 × 180 100 × 180 100 × 180 100 × 180 100 × 180 100 × 180	39.6/4.0 39.6/4.0 39.6/4.0 30.0/3.9 50.0/4.3 39.6/4.0 39.6/4.0 39.6/4.0 39.6/4.0 45.4/4.1 39.6/4.0 39.6/4.0 39.6/4.0 39.6/4.0 39.6/4.0 39.6/4.0 39.6/4.0 39.6/4.0 45.4/3.9 39.6/4.0 39.6/4.0 30.0/3.9 50.0/4.1 39.6/4.0 39.6/4.0	2 × 2 2 × 2 2 × 2 2 × 2 2 × 2 2 × 2 2 × 2 2 × 2 2 × 2 2 × 2 2 × 2 2 × 2 2 × 2 2 × 2 2 × 2 2 × 2 2 × 2 2 × 2 2 × 2 2 × 2 2 × 2 2 × 2 2 × 2	D6C + D12T D6C + D12T D6C + D12T D6C + D12T D6C + D12T D6C + D12T D6C + D12T D6C + D12T D6C + D12T D6C + D12T D6C + D12T D6C + D12T D6C + D12T D6C + D12T D6C + D12T D6C + D12T D6C + D12T D6C + D12T D6C + D12T D6C + D12T D6C + D12T D6C + D12T D6C + D12T D6C + D12T D6C + D12T	Y-R6@50c/c Y-R6@50c/c Y-R6@50c/c Y-R6@50c/c Y-R6@50c/c Y-R6@50c/c Y-R6@50c/c Y-R6@50c/c Y-R6@50c/c Y-R6@50c/c Y-R6@50c/c Y-R6@50c/c Y-R6@50c/c Y-R6@50c/c Y-R6@50c/c Y-R6@50c/c Y-R6@50c/c Y-R6@50c/c Y-R6@50c/c Y-R6@50c/c Y-R6@50c/c Y-R6@50c/c Y-R6@50c/c Y-R6@50c/c Y-R6@50c/c	- - - - - EBR-CFRP Sheet EBR-CFRP Sheet EBR-CFRP Sheet EBR-CFRP Sheet EBR-CFRP Sheet EBR-CFRP Sheet EBR-CFRP Sheet EBR-CFRP Sheet EBR-CFRP Sheet EBR-CFRP Sheet	- - - - - 1 1 1 1 2 1 1 1 1 1 1 1 1 2 1 1 1 1 1 1	- - - - - 0.167 0.167 0.167 0.167 0.167 0.167 0.167 0.167 0.167 0.167 0.167 0.167 0.167 0.167 0.167 0.167 0.167 0.167 0.167 0.167	- - - - - CFRP sheet, 1.4 m in length, applied at the bottom	No Opening provided			34 25 44 33 35 36 39 41 42 47 43 47 52 50 49 48 47 46 52 42 56 45 55 54 55	42 31 53 40 43 43 48 50 51 57 52 57 63 61 60 59 58 56 64 51 69 54 67 66 67	23.2 29.0 17.3 17.5 23.2 7.0 9.2 10.7 12.5 10.9 17.1 20.9 28.6 28.0 27.8 27.8 27.5 27.2 27.3 30.6 23.4 21.7 29.5 16.0 12.7	23.2 29.0 17.3 17.5 23.2 7.0 9.2 10.7 12.5 10.9 17.1 20.9 28.6 28.0 27.8 27.8 27.5 27.2 27.3 30.6 23.4 21.7 29.5 16.0 12.7	21 24 29 26 23 22 18 20 24 15 27 32 36 28 27 30 19 18 14 33 22 26 24 29 30	Concrete Crushing Concrete Crushing Concrete Crushing Concrete Crushing Concrete Crushing End debonding End debonding Intermediate debonding Intermediate debonding Intermediate debonding Concrete Crushing Concrete Crushing Intermediate debonding Intermediate debonding Intermediate debonding Intermediate debonding Intermediate debonding Intermediate debonding Intermediate debonding Intermediate debonding Intermediate debonding Intermediate debonding Intermediate debonding Concrete Crushing Concrete Crushing	[62]
B1/NC B2/NC B3/NC B4/NC B5/NC B6/NC B7/NC B8/NC B9/NC B10/NC B11/NC B12/NC B13/NC B14/NC B15/NC B16/NC B17/NC B18/NC	1.5 1.5 1.5 1.5 1.5 1.5 1.5 1.5 1.5 1.5 1.5 1.5 1.5 1.5 1.5 1.5 1.5 1.5	150 × 500 150 × 500 150 × 500 150 × 500 150 × 500 150 × 500 150 × 500 150 × 500 150 × 500 150 × 500 150 × 500 150 × 500 150 × 500 150 × 500 150 × 500 150 × 500 150 × 500 150 × 500	27.0/3.1 27.0/3.1 27.0/3.1 27.0/3.1 27.0/3.1 27.0/3.1 27.0/3.1 27.0/3.1 27.0/3.1 27.0/3.1 27.0/3.1 27.0/3.1 27.0/3.1 27.0/3.1 27.0/3.1 27.0/3.1 27.0/3.1 27.0/3.1	2 × bars at the top 3 × bars at the bottom 8 × bars having Ø6 mm @ tension and compression zone with 86 mm c/c	D6C + D16T D6C + D16T D6C + D16T D6C + D16T D6C + D16T D6C + D16T D6C + D16T D6C + D16T D6C + D16T D6C + D16T D6C + D16T D6C + D16T D6C + D16T D6C + D16T D6C + D16T D6C + D16T D6C + D16T D6C + D16T	Y-R6@90c/c Y-R6@90c/c Y-R6@90c/c Y-R6@90c/c Y-R6@90c/c Y-R6@90c/c Y-R6@90c/c Y-R6@90c/c Y-R6@90c/c Y-R6@90c/c Y-R6@90c/c Y-R6@90c/c Y-R6@90c/c Y-R6@90c/c Y-R6@90c/c Y-R6@90c/c Y-R6@90c/c Y-R6@90c/c	- - EBR CFRP Sheet EBR CFRP Sheet EBR CFRP Sheet EBR CFRP Sheet EBR CFRP Sheet EBR CFRP Sheet EBR CFRP Sheet EBR CFRP Sheet	- - - 4 - 4 - 4 - 4 - 4 - 4 - 4 - 4	- - 0.167 0.167 0.167 0.167 0.167 0.167 0.167 0.167 0.167 0.167 0.167 0.167 0.167 0.167 0.167 0.167	- - Rectangle shape along the openings	- - 2 Square (C) 2 Square (C) 2 Square (C) 2 Square (C) 2 Square (E) 2 Square (E) 2 Square (E) 2 Square (E) 2 Square (C) 2 Square (C) 2 Square (C) 2 Square (C) 2 Square (E) 2 Square (E) 2 Square (E) 2 Square (E)	- - 200 × 200 200 × 200 230 × 230 230 × 230 200 × 200 200 × 200 230 × 230 230 × 230 200 × 200 200 × 200 230 × 230 230 × 230 200 × 200 200 × 200 230 × 230 230 × 230	- - No CFRP Reinforcement	395 442 150 221 138 165 165 237 134 197 189 229 158 197 197 261 166 237	500 560 190 280 175 210 210 300 170 250 240 290 200 250 250 330 210 300	9.0 8.2 4.0 5.3 4.3 6.2 4.8 6.2 5.1 6.8 4.0 5.1 4.0 5.5 4.0 7.0 4.6 6.1	- - - - - - - - - - - - - - - - - -	16 14 17 18 13 14 16 18 13 19 12 14 15 19 21 18 16 18	Flexure Failure Flexure Failure Shear Failure Shear Failure & debonding Shear Failure Shear Failure & debonding Shear Failure Shear, debonding, Rupture Shear, Rupture Shear, debonding, Rupture Shear, Rupture Shear, debonding, Rupture Shear, Rupture Shear, debonding, Rupture Shear, Rupture Shear, debonding, Rupture Shear, Rupture Shear, debonding, Rupture	[47]
DB1/NC DB2/NC DB3/NC DB4/NC DB5/NC DB6/NC DB7/NC	1.1 1.1 1.1 1.1 1.1 1.1 1.1	150 × 240 150 × 240 150 × 240 150 × 240 150 × 240 150 × 240 150 × 240	17.3/- 17.3/- 17.3/- 17.3/- 17.3/- 17.3/- 17.3/-	2 × 3 2 × 3 2 × 3 2 × 3 2 × 3 2 × 3 2 × 3	D6C + D16T D6C + D16T D6C + D16T D6C + D16T D6C + D16T D6C + D16T D6C + D16T	Y-R6@100c/c Y-R6@100c/c Y-R6@100c/c Y-R6@100c/c Y-R6@100c/c Y-R6@100c/c Y-R6@100c/c	- EBR CFRP Strips EBR CFRP Strips EBR CFRP Strips	- 10 8 6 6 4 4	- 0.15 0.15 0.15 0.15 0.15 0.15	- U-Wrapped U-Wrapped U-Wrapped 45° Inclined 45° Inclined 45° Inclined	No Opening Provided			64 74 76 77 72 77 83	98 110 110 109 102 105 108	1.1 1.2 1.2 1.2 1.1 1.1 1.0	1.1 1.2 1.2 1.2 1.1 1.1 1.0	7 5 4 6 8 6 7	Flexure Failure Flexure Failure, Debonding Flexure Failure, Rupture Flex., Debonding, Rupture Flex., Debonding, Rupture Flex., Debonding, Rupture Flex., Debonding, Rupture	[51]
R-DB-160/NC R-DB-240/NC R-DB-320/NC HS-DB-160 HS-DB-240 HS-DB-320 VS-DB-160 VS-DB-240 VS-DB-320	1.6 1.6 1.6 1.6 1.6 1.6 1.6 1.6 1.6	250 × 400 250 × 400 250 × 400 250 × 400 250 × 400 250 × 400 250 × 400 250 × 400 250 × 400	30.0/3.3 30.0/3.3 30.0/3.3 30.0/3.3 30.0/3.3 30.0/3.3 30.0/3.3 30.0/3.3 30.0/3.3	2 bars at top, 3 bars at bottom, 4 bars of Ø6 mm were in center	D12C + D16T D12C + D16T D12C + D16T D12C + D16T D12C + D16T D12C + D16T D12C + D16T D12C + D16T D12C + D16T	Y-R6@80c/c Y-R6@80c/c Y-R6@80c/c Y-R6@80c/c Y-R6@80c/c Y-R6@80c/c Y-R6@80c/c Y-R6@80c/c Y-R6@80c/c	- - - EBR–Post-Tensioning Strands EBR–Post-Tensioning Strands	- - - 12 12 12 12 12 12	12.7 12.7 12.7 12.7 12.7 12.7 12.7 12.7 12.7	- - - Horizontal Horizontal Horizontal Vertical Vertical Vertical	2 Rectangle 2 Rectangle 2 Rectangle 2 Rectangle 2 Rectangle 2 Rectangle 2 Rectangle 2 Rectangle 2 Rectangle	160 × 160 160 × 240 160 × 320 160 × 160 160 × 240 160 × 320 160 × 160 160 × 240 160 × 320	- Horizontal Strengthening with Exterior Strands Vertical Strengthening with Exterior Strands	70 90 80 50 75 60 35 55 40	220 290 280 170 260 230 150 230 200	4.0 5.2 8.4 4.5 6.7 8.1 4.7 6.3 7.3	- - - - - - - - -	19 23 20 21 18 16 19 15 16	Diagonal Splitting Failure Shear Compression Failure Diagonal Splitting Failure Diagonal Splitting Failure Diagonal Splitting Failure Diagonal Splitting Failure Shear Compression Failure Shear Compression Failure Shear Compression Failure	[81]
CB/NC SB-100/NC DB-100/NC SB-71/NC DB-71/NC SB-40/NC DB-40/NC SB-23/NC DB-23/NC	1.6 1.6 1.6 1.6 1.6 1.6 1.6 1.6 1.6	160 × 200 160 × 200 160 × 200 160 × 200 160 × 200 160 × 200 160 × 200 160 × 200 160 × 200	64.5/2.8 68.0/4.8 68.0/4.8 75.6/6.1 75.6/6.1 64.0/5.3 64.0/5.3 70.5/6.6 70.5/6.6	2 × 3 2 × 3 2 × 3 2 × 3 2 × 3 2 × 3 2 × 3 2 × 3 2 × 3	D12C + D12T D12C + D12T D12C + D12T D12C + D12T D12C + D12T D12C + D12T D12C + D12T D12C + D12T D12C + D12T	3Y-R8@40c/c 3Y-R8@40c/c 3Y-R8@40c/c 3Y-R8@40c/c 3Y-R8@40c/c 3Y-R8@40c/c 3Y-R8@40c/c 3Y-R8@40c/c 3Y-R8@40c/c	- EBR-CFRP Sheets EBR-CFRP Sheets EBR-CFRP Sheets EBR-CFRP Sheets	- 1 2 1 2 1 2 1 2	- 0.17 0.17 0.17 0.17 0.17 0.17 0.17 0.17	- Vertical Vertical Vertical Vertical Vertical Vertical Vertical Vertical	No Opening Provided			68 92 107 102 122 110 123 124 136	173 220 232 212 249 216 237 235 256	6.2 6.8 4.0 5.1 7.0 4.6 5.2 4.5 4.3	6.2 6.8 4.0 5.1 7.0 4.6 5.2 4.5 4.3	14 16 13 19 15 18 16 20 17	Flexure-Shear Failure Diagonal Splitting Failure Diagonal Splitting Failure Nodal Failure Dia-Compression Failure Diagonal Splitting Failure Flexure Shear Failure Diagonal Splitting Failure Diagonal Splitting Failure	[63]
B1-NC B2-NC B3-NC B4-NC B5-NC B6-NC	1.84 1.84 1.84 1.84 1.84 1.84	140 × 350 140 × 350 140 × 350 140 × 350 140 × 350 140 × 350	37.0/3.5 37.0/3.5 37.0/3.5 37.0/3.5 37.0/3.5 37.0/3.5	2 top & bottom each, 6 bars of Ø8@ 300c/c	D12C + D12T D12C + D12T D12C + D12T D12C + D12T D12C + D12T D12C + D12T	Y-R8@80c/c Y-R8@80c/c Y-R8@80c/c Y-R8@80c/c Y-R8@80c/c Y-R8@80c/c	EBR-CFRP Sheet EBR-CFRP Sheet EBR-CFRP Sheet	8 8 8 8 8 8	0.17 0.17 0.17 0.17 0.17 0.17	Vertical Vertical Vertical Vertical Vertical Vertical	No Opening Provided	30 × 377 30 × 377 30 × 377 30 × 377 30 × 377 30 × 377	2 x Bolted Shape Struts placed diagonally at an angle of 45°	353 360 340 332 250 239	905 858 740 691 510 468	4.2 4.9 5.6 5.3 6.4 7.8	- - - - - -	18 14 17 22 20 19	Flexure-Shear Failure Diagonal Splitting Failure Diagonal Splitting Failure Diagonal Splitting Failure Diagonal Splitting Failure Flexure-Shear Failure	[70]
BS/NC BOCC-230/NC BOCC-300/NC BOCC-370/NC BOCB-230/NC BOCT-230/NC BOSC-300/NC BOC-300 S45° BOC-370 S0° BOC-370 S45° BOC-370 S90° BC-300 Re45° BC-370 Re45°	1.8 1.8 1.8 1.8 1.8 1.8 1.8 1.8 1.8 1.8 1.8 1.8 1.8	120 × 750 120 × 750 120 × 750 120 × 750 120 × 750 120 × 750 120 × 750 120 × 750 120 × 750 120 × 750 120 × 750 120 × 750 120 × 750	28.0/3.1 28.0/3.1 28.0/3.1 28.0/3.1 28.0/3.1 28.0/3.1 28.0/3.1 28.0/3.1 28.0/3.1 28.0/3.1 28.0/3.1 28.0/3.1 28.0/3.1	2 bars at top, 2 bars at bottom, 8 bars of Ø8 mm @ 150c/c	D8C + D12T D8C + D12T D8C + D12T D8C + D12T D8C + D12T D8C + D12T D8C + D12T D8C + D12T D8C + D12T D8C + D12T D8C + D12T D8C + D12T D8C + D12T	Y-R8@150c/c Y-R8@150c/c Y-R8@150c/c Y-R8@150c/c Y-R8@150c/c Y-R8@150c/c Y-R8@150c/c Y-R8@150c/c Y-R8@150c/c Y-R8@150c/c Y-R8@150c/c Y-R8@150c/c Y-R8@150c/c	- EBR-CFRP Sheet EBR-CFRP Sheet EBR-CFRP Sheet EBR-CFRP Sheet EBR-CFRP Sheet EBR-CFRP Sheet	- - - - - - - 16 8 16 16 16 16	- - - - - - - 0.17 0.17 0.17 0.17 0.17 0.17	- - - - - - - Diagonal 45° Horizontal Diagonal 45° Perpendicular Diagonal 45° Diagonal 45°	- 2 Circle (C) 2 Circle (C) 2 Circle (C) 2 Circle (BE) 2 Circle (SE) 2 Square (C) 2 Circle (C) 2 Circle (C) 2 Circle (C) 2 Circle (C) 2 Circle (C) 2 Circle (C)	- Ø225 Ø300 Ø375 Ø225 Ø300 300 × 300 Ø300 Ø375 Ø375 Ø375 Ø300 Ø375	- - - - - Diagonal 45° angle Horizontal Diagonal 45° angle Perpendicular Diagonal 45° angle	86 105 87 72 103 102 73 200 143 182 156 134 90	220 250 190 150 210 200 140 377 270 343 295 252 169	1.65 2.75 2.66 3.15 3.28 4.20 4.36 2.00 2.14 2.00 2.56 3.60 3.73	116 77 68 79 55 57 100 96 91 83 104 68 69	16 19 13 17 12 19 19 17 15 16 20 18 15	Flexure Failure Flexure Failure Diagonal Splitting Failure Diagonal Splitting Failure Diagonal Splitting Failure Diagonal Splitting Failure Flexure Failure Diagonal Splitting Failure Diagonal Splitting Failure Diagonal Splitting Failure Diagonal Splitting Failure Flexure & Shear Failure Flexure Failure	[71]
D1/NC D2/NC D3/NC D4/NC D4-1/NC D4-2/NC D4-3/NC D5/NC D5-1/NC D5-2/NC D6/NC D6-1/NC D7/NC D8/NC D8-1/NC D8-2/NC D10/NC D10-1/NC D10-2/NC D10-3/NC	2.4 2.4 2.4 2.4 2.4 2.4 2.4 2.4 2.4 2.4 2.4 2.4 2.4 2.4 2.4 2.4 2.4 2.4 2.4 2.4	140 × 180 140 × 180 140 × 180 140 × 180 140 × 180 140 × 180 140 × 180 140 × 180 140 × 180 140 × 180 140 × 180 140 × 180 140 × 180 140 × 180 140 × 180 140 × 180 140 × 180 140 × 180 140 × 180 140 × 180	44.8/3.6 44.8/3.6 44.8/3.6 44.8/3.6 44.8/3.6 44.8/3.6 44.8/3.6 44.8/3.6 44.8/3.6 44.8/3.6 44.8/3.6 44.8/3.6 44.8/3.6 44.8/3.6 44.8/3.6 44.8/3.6 44.8/3.6 44.8/3.6 44.8/3.6 44.8/3.6	2 × 2 2 × 2 2 × 2 2 × 2 2 × 2 2 × 2 2 × 2 2 × 2 2 × 2 2 × 2 2 × 2 2 × 2 2 × 2 2 × 2 2 × 2 2 × 2 2 × 2 2 × 2 2 × 2 2 × 2	D6C + D8T D6C + D8T D6C + D8T D6C + D8T D6C + D8T D6C + D8T D6C + D8T D6C + D8T D6C + D8T D6C + D8T D6C + D8T D6C + D8T D6C + D8T D6C + D8T D6C + D8T D6C + D8T D6C + D8T D6C + D8T D6C + D8T D6C + D8T	Y-R8@100c/c Y-R8@100c/c Y-R8@100c/c Y-R8@100c/c Y-R8@100c/c Y-R8@100c/c Y-R8@100c/c Y-R8@100c/c Y-R8@100c/c Y-R8@100c/c Y-R8@100c/c Y-R8@100c/c Y-R8@100c/c Y-R8@100c/c Y-R8@100c/c Y-R8@100c/c Y-R8@100c/c Y-R8@100c/c Y-R8@100c/c Y-R8@100c/c	- - - - EBR-CFRP Laminate EBR-CFRP Laminate EBR-CFRP Laminate EBR-CFRP Laminate EBR-CFRP Laminate EBR-CFRP Laminate EBR-CFRP Laminate EBR-CFRP Laminate	- - - - 1 2 3 - 1 2 - 1 - - 1 2 - 1 2 3	1.4 1.4 1.4 1.4 1.4 1.4 1.4 1.4 1.4 1.4 1.4 1.4 1.4 1.4 1.4 1.4 1.4 1.4 1.4 1.4	- - - - Beam Bottom	- - - - 1 Groove 2 Grooves 3 Grooves - 1 Groove 2 Grooves - 1 Groove - - 1 Groove 2 Grooves - 1 Groove 2 Grooves 3 Grooves	- - - - 10 × 13 5 × 13 3.5 × 13 - 10 × 13 5 × 13 - 10 × 13 - - 10 × 13 5 × 13 - 10 × 13 5 × 13 3.5 × 13	- - - - Top Center Longitudinal - Top Center Longitudinal - Top Center Longitudinal	14 15 17 16 20 24 24 23 25 29 28 26 28 22 27 29 27 29 33 33	36 35 36 34 40 47 46 43 47 55 52 49 52 42 51 54 51 55 63 61	19.6 13.4 15.8 16.7 19.3 22.4 21.8 25.3 26.7 24.1 23.2 24.6 25.9 18.4 27.0 29.2 20.8 24.0 32.7 31.4	- - - - - - - - - - - - - - - - - - - -	24 29 25 28 26 26 30 29 32 36 33 32 35 31 27 26 32 28 24 33	Flexure Failure Flexure Failure Flexure Failure Flexure Failure Intermediate debonding of Concrete & FRP Intermediate Debonding of Concrete & FRP Flexure & Shear Failure Intermediate Debonding of Concrete & FRP Flexure Failure Intermediate Debonding of Concrete & FRP Flexure Failure Intermediate Debonding of Concrete & FRP Flexure Failure Intermediate Debonding of Concrete & FRP	[68]
C Beam/NC S Beam/NC S-EBR-W/NC S-EBR-V/NC S-EBROG-V S-EBR-D/NC S-EBROG-D E Beam/NC E-EBR-D E-EBROG-D	1.0 1.0 1.0 1.0 1.0 1.0 1.0 1.0 1.0 1.0	100 × 500 100 × 500 100 × 500 100 × 500 100 × 500 100 × 500 100 × 500 100 × 500 100 × 500 100 × 500	53.6/4.3 53.6/4.3 53.6/4.3 53.6/4.3 53.6/4.3 53.6/4.3 53.6/4.3 53.6/4.3 53.6/4.3 53.6/4.3	2 bars at top, 2 bars at bottom, 8 bars of Ø 6 mm @ 120c/c	D12C + D18T D12C + D18T D12C + D18T D12C + D18T D12C + D18T D12C + D18T D12C + D18T D12C + D18T D12C + D18T D12C + D18T	Y-R6@120c/c Y-R6@120c/c Y-R6@120c/c Y-R6@120c/c Y-R6@120c/c Y-R6@120c/c Y-R6@120c/c Y-R6@120c/c Y-R6@120c/c Y-R6@120c/c	- - EBR-CFRP Strips EBR-CFRP Strips EBR-CFRP Strips EBR-CFRP Strips	- - 9 10 5 10 9 5 9 10	- - 0.9 0.9 0.9 0.9 0.9 0.9 0.9 0.9	- - Vertical Diagonal Diagonal Diagonal Vertical Diagonal Diagonal Diagonal	- - 2 Circles (C) 1 Circle (SC) 2 Circles (C) 2 Circles (C) 1 Circle (SC) 2 Circles (C) 2 Circles (C) 1 Circle (SC)	- - Ø200 Ø200 Ø200 Ø200 Ø200 Ø200 Ø200 Ø200	- - Center Left Side Center Center Left Side Center Center	281 124 172 147 171 216 215 183 250 309	720 295 374 307 350 423 413 345 470 583	9.68 7.95 8.89 6.96 6.22 8.14 7.08 5.66 6.79 10.57	- - - - - - - - - -	8 5 9 6 7 7 8 6 6 5	Flexure Failure Flexure Failure Intermediate Debonding of Concrete & FRP Intermediate Debonding of Concrete & FRP Intermediate Debonding of Concrete & FRP Intermediate Debonding of Concrete & FRP	[69]
SP1/NC SP2/NC SP3/NC SP4/NC SP5/NC SP6/NC SP7/NC SP8/NC	1.8 1.8 1.8 1.8 1.8 1.8 1.8 1.8	150 × 500 150 × 500 150 × 500 150 × 500 150 × 500 150 × 500 150 × 500 150 × 500	50.0/4.1 60.0/4.6 50.0/4.1 50.0/4.1 50.0/4.1 60.0/4.6 60.0/4.6 60.0/4.6	2 × 2 2 × 2 2 × 2 2 × 2 2 × 2 2 × 2 2 × 2 2 × 2	D10C + D12T D10C + D12T D10C + D12T D10C + D12T D10C + D12T D10C + D12T D10C + D12T D10C + D12T	Y-R8@175c/c Y-R8@175c/c Y-R8@175c/c Y-R8@175c/c Y-R8@175c/c Y-R8@175c/c Y-R8@175c/c Y-R8@175c/c	Steel NSM-GFRP Bars NSM-GFRP Bars NSM-GFRP Bars	13 13 13 13 13 13 13 13	8, 10, 12 8, 10, 12 8, 10, 12 8, 10, 12 8, 10, 12 8, 10, 12 8, 10, 12 8, 10, 12	- - - - - - - -	No Opening Provided			170 175 110 117 120 115 118 130	659 426 538 675 693 451 570 720	12.24 11.88 5.70 6.58 9.70 9.50 9.30 7.24	12.24 11.88 5.70 6.58 9.70 9.50 9.30 7.24	24 27 11 18 9 14 13 14	Flexure Failure Flexure Failure Flexure Failure Flexure Failure Flexure Failure Flexure Failure Flexure Failure Flexure Failure	[83]
V-3/12/NC L30-3/12/NC L45-3/12/NC L60-3/12/NC L45-2/8/NC L45-3/16/NC	1.53 1.53 1.53 1.53 1.53 1.53	150 × 240 150 × 240 150 × 240 150 × 240 150 × 240 150 × 240	28.4/2.8 30.4/3.0 29.4/2.8 27.1/2.6 28.2/2.7 28.7/3.2	2 × 3 2 × 3 2 × 3 2 × 3 2 × 2 2 × 3	D8C + D12T D8C + D12T D8C + D12T D8C + D12T D8C + D18T D8C + D16T	Y-R8@100c/c 4-Ø8mm 30° 4-Ø8mm 45° 4-Ø8mm 60° Y-R8@100c/c 4-Ø8mm 45°	Vertical Sti. Steel Lac—30° Steel Lac—45° Steel Lac—60° Vertical Sti. Steel Lac—45°	- - - - - -	- - - - - -	- - - - - -	No Opening Provided			142 162 141 148 55 178	171 183 186 188 104 213	15.23 12.61 17.86 14.76 14.62 11.36	15.23 12.61 17.86 14.76 14.62 11.36	19 29 20 24 23 26	Flexure & Shear Failure Flexure & Shear Failure Flexure Failure Flexure Failure Flexure Failure Shear Failure	[87]
CB/NC H105/NC H62,5/NC H60/NC B30/NC B50/NC B70/NC SB30/NC SB50/NC SB70/NC CUW/NC DUW/NC	1.1 1.1 1.1 1.1 1.1 1.1 1.1 1.1 1.1 1.1 1.1 1.1	100 × 160 100 × 160 100 × 160 100 × 160 100 × 160 100 × 160 100 × 160 100 × 160 100 × 160 100 × 160 100 × 160 100 × 160	21.5/3.0 21.5/3.0 21.5/3.0 21.5/3.0 21.5/3.0 21.5/3.0 21.5/3.0 21.5/3.0 21.5/3.0 21.5/3.0 21.5/3.0 21.5/3.0	2 × 2 2 × 2 2 × 2 2 × 2 2 × 2 2 × 2 2 × 2 2 × 2 2 × 2 2 × 2 2 × 2 2 × 2	D8C + D8T D8C + D8T D8C + D8T D8C + D8T D8C + D8T D8C + D8T D8C + D8T D8C + D8T D8C + D8T D8C + D8T D8C + D8T D8C + D8T	Y-R6@100c/c Y-R6@100c/c Y-R6@100c/c Y-R6@100c/c Y-R6@100c/c Y-R6@100c/c Y-R6@100c/c Y-R6@100c/c Y-R6@100c/c Y-R6@100c/c Y-R6@100c/c Y-R6@100c/c	- EBR-CFRP Sheet EBR-CFRP Sheet EBR-CFRP Sheet EBR-CFRP Sheet EBR-CFRP Sheet	- 1 1 1 1 1 1 1 1 1 1 1	- 1.2 1.2 1.2 1.2 1.2 1.2 1.2 1.2 1.2 1.2 1.2	- Beam Bottom	1 Rect. 1 Rect. 1 Rect. 1 Rect. 1 Rect. 1 Rect. 1 Rect. 1 Rect. 1 Rect. 1 Rect. 1 Rect. 1 Rect.	40 × 30 40 × 30 40 × 30 40 × 30 40 × 30 40 × 50 40 × 70 40 × 30 40 × 50 40 × 70 40 × 30 40 × 30	Center Center Top Center Bottom Center Center Top Center Bottom Center Center Top Center Bot. Center	15 9 10 9 10 9 8 4 4 3 9 7	44 40 41 40 41 40 39 56 52 44 129 88	14.99 8.81 10.1 9.19 10.1 9.27 7.92 4.43 3.7 3.05 8.74 7.31	- - - - - - - - - - - -	13 16 18 12 15 21 18 16 19 24 25 14	Flexure Failure Flexure & Concrete Crushing Flexure & Concrete Crushing Flexure & Concrete Crushing Flexure & Concrete Crushing Flexure & Concrete Crushing Flex. & Concrete Crushing	[64]
A1/NC A2-C/NC A3-C/NC A4-C/NC A5-C/NC B1/NC B2-C/NC B3-C/NC B4-C/NC B5-C/NC	1.2 1.2 1.2 1.2 1.2 1.2 1.2 1.2 1.2 1.2	150 × 250 150 × 250 150 × 250 150 × 250 150 × 250 150 × 250 150 × 250 150 × 250 150 × 250 150 × 250	46.6/3.9 46.6/3.9 46.6/3.9 46.6/3.9 46.6/3.9 19.0/2.6 19.0/2.6 19.0/2.6 19.0/2.6 19.0/2.6	2 × 2 2 × 2 2 × 2 2 × 2 2 × 2 2 × 2 2 × 2 2 × 2 2 × 2 2 × 2	D10C + D10T D10C + D10T D10C + D10T D10C + D10T D10C + D10T D10C + D10T D10C + D10T D10C + D10T D10C + D10T D10C + D10T	Y-R10@50c/c Y-R10@50c/c Y-R10@50c/c Y-R10@50c/c Y-R10@50c/c Y-R10@50c/c Y-R10@50c/c Y-R10@50c/c Y-R10@50c/c Y-R10@50c/c	- NSM-CFRP Rope NSM-CFRP Rope - NSM-CFRP Rope NSM-CFRP Rope	- 1 2 5 1 - 1 2 5 1	- >0.034 in^2^ >0.034 in^2^ >0.034 in^2^ >0.034 in^2^ - >0.034 in^2^ >0.034 in^2^ >0.034 in^2^ >0.034 in^2^	- Beam Center Beam Center Beam Center Beam Center - Beam Center Beam Center Beam Center Beam Center	No Opening Provided			53 87 101 106 69 64 93 107 99 74	137 208 220 221 140 125 178 202 186 139	52.6 56.8 59.7 59.8 55.3 39.1 21.0 37.7 19.5 45.8	52.6 56.8 59.7 59.8 55.3 39.1 21.0 37.7 19.5 45.8	14 12 15 9 12 6 5 10 11 6	Flexure Failure Flexure & Shear Failure Shear Failure Flexure & Shear Failure Flexure Failure Flexure Failure Flexure & Shear Failure Shear Failure Flexure & Shear Failure Flexure Failure	[88]
G6-T20-R1 GSF-T20-R1 GSF-T400-R1 GSFT400R1U GSFT400R2U GSFT600R1 GSFT600R1U GSFT600R2U G10SF-T20R1 G10SFT600R1	1.1 1.1 1.1 1.1 1.1 1.1 1.1 1.1 1.1 1.1	150 × 150 150 × 150 150 × 150 150 × 150 150 × 150 150 × 150 150 × 150 150 × 150 150 × 150 150 × 150	71.5/5.7 71.5/5.7 62.8/2.6 62.8/2.6 62.8/2.6 32.5/1.1 32.5/1.1 32.5/1.1 71.5/5.7 32.5/1.1	2 × 2 2 × 2 2 × 2 2 × 2 2 × 2 2 × 2 2 × 2 2 × 2 2 × 2 2 × 2	D8C + D10T D8C + D10T D8C + D10T D8C + D10T D8C + D10T D8C + D10T D8C + D10T D8C + D10T D8C + D10T D8C + D10T	2xY-R6@60c/c 2xY-R6@60c/c 2xY-R6@60c/c 2xY-R6@60c/c 2xY-R6@60c/c 2xY-R6@60c/c 2xY-R6@60c/c 2xY-R6@60c/c 2xY-R6@60c/c 2xY-R6@60c/c	Steel NSM-GFRP Rod & EBR CFRP Sheet NSM-GFRP Rod & EBR CFRP Sheet NSM-GFRP Rod & EBR CFRP Sheet	- 8 8 8 8 8 8 8 8 8	- 0.166 0.166 0.166 0.166 0.166 0.166 0.166 0.166 0.166	- Flexure Zone Flexure Zone Flexure Zone Flexure Zone Flexure Zone Flexure Zone Flexure Zone Flexure Zone Flexure Zone	No Opening Provided			13 10 5 5 15 8 20 17 24 21	28 22 11 11 34 18 43 37 52 46	19.5 18.6 27.6 15.3 20 13.1 8.9 8.5 11.2 18.6	19.5 18.6 27.6 15.3 20 13.1 8.9 8.5 11.2 18.6	23 21 24 24 17 24 15 18 16 18	Plate end Debonding Plate end Debonding Plate end Debonding Plate end Debonding Plate end Debonding Plate end Debonding GFRP Bar Rupture GFRP Bar Rupture Concrete Crushing Concrete Crushing	[84]
CB/NC SB-1/NC SB-2/NC SB-3/NC	3.5 3.5 3.5 3.5	200 × 325 200 × 325 200 × 325 200 × 325	31.9/3.0 31.9/3.0 31.9/3.0 31.9/3.0	2 × 3 2 × 4 2 × 3 2 × 5	D12C + D16T D12C + D16T D12C + D16T D12C + D16T	Y-R12@100c/c Y-R12@100c/c Y-R12@100c/c Y-R12@100c/c	- EBR-SPHECC EBR-SPHECC EBR-SPHECC	- 1 2 3	- 50 50 50	- Flexure Zone Flexure & Shear	No Opening Provided			66 90 143 170	169 214 310 354	15.6 18.2 11.9 13.9	15.6 18.2 11.9 13.9	11 7 5 2	Interface De-Bonding Interface De-Bonding Interface De-Bonding Interface De-Bonding	[86]
BL/NC BH/NC BL-C/NC BL-P/NC BL-G/NC BH-C/NC BH-P/NC BH-G/NC	2.5 2.5 2.5 2.5 2.5 2.5 2.5 2.5	150 × 260 150 × 260 150 × 260 150 × 260 150 × 260 150 × 260 150 × 260 150 × 260	39.5/3.6 39.5/3.6 39.5/3.6 39.5/3.6 39.5/3.6 39.5/3.6 39.5/3.6 39.5/3.6	2 × 2 2 × 2 2 × 2 2 × 2 2 × 2 2 × 2 2 × 2 2 × 2	D8C + D10T D8C + D10T D8C + D16T D8C + D16T D8C + D16T D8C + D16T D8C + D16T D8C + D16T	Y-R8@100c/c Y-R8@100c/c Y-R8@100c/c Y-R8@100c/c Y-R8@100c/c Y-R8@100c/c Y-R8@100c/c Y-R8@100c/c	- - EBR-Mortar, CFRP, Mortar PBO, Mortar, GFRP, Mortar, CFRP, Mortar	- - 4 4 4 4 4 4	- - 0.047 0.0115 0.066 0.047 0.0115 0.066	- - Flexure Zone (BB) Flexure Zone (BB) Flexure Zone (BB)	- - 1 Rectangle 1 Rectangle 1 Rectangle 1 Rectangle 1 Rectangle 1 Rectangle	- - 150 × 15 150 × 15 150 × 15 150 × 15 150 × 15 150 × 15	- - Beam Bottom	27 25 22 30 18 21 20 17	40 99 83 83 58 144 136 124	27.2 24.7 21.6 29.7 17.9 20.5 20.2 17.1	3.53 7.85 5.9 5.5 4.1 10.1 9.3 9.8	11 18 16 22 15 19 15 14	Flexure & Concrete Crushing Flexure & Concrete Crushing Flexure & Concrete Crushing Flexure & Concrete Crushing	[74]
NS-150-C/NC NS-200-C/NC NS-250-C/NC FS-200-C/NC FS-250-C/NC NS-150-T/NC NS-200-T/NC NS-250-T/NC FS-250-T/NC NS-150-B/NC NS-200-B/NC NS-250-B/NC FS-250-B/NC	1.2 1.2 1.2 1.2 1.2 1.2 1.2 1.2 1.2 1.2 1.2 1.2 1.2	80 × 500 80 × 500 80 × 500 80 × 500 80 × 500 80 × 500 80 × 500 80 × 500 80 × 500 80 × 500 80 × 500 80 × 500 80 × 500	21.0/2.9 21.0/2.9 21.0/2.9 21.0/2.9 21.0/2.9 21.0/2.9 21.0/2.9 21.0/2.9 21.0/2.9 21.0/2.9 21.0/2.9 21.0/2.9 21.0/2.9	2 × 4 2 × 4 2 × 4 2 × 4 2 × 4 2 × 4 2 × 4 2 × 4 2 × 4 2 × 4 2 × 4 2 × 4 2 × 4	D8C + D14T. D8C + D14T. D8C + D14T. D8C + D14T. D8C + D14T. D8C + D14T. D8C + D14T. D8C + D14T. D8C + D14T. D8C + D14T. D8C + D14T. D8C + D14T. D8C + D14T.	Y-R6@150c/c Y-R6@150c/c Y-R6@150c/c Y-R6@150c/c Y-R6@150c/c Y-R6@150c/c Y-R6@150c/c Y-R6@150c/c Y-R6@150c/c Y-R6@150c/c Y-R6@150c/c Y-R6@150c/c Y-R6@150c/c	-. - - EBR-CFRP Strips - - - EBRCFRP Strips EBRCFRP Strips -	- - - 16 16 - - - 16 - - - 16	- - - 0.381 0.381 - - - 0.381 - - - 0.381	- - - Near Openings Near Openings - - - Near Openings - -	2 Square 2 Square 2 Square 2 Square 2 Square 2 Square 2 Square 2 Square 2 Square 2 Square 2 Square 2 Square 2 Square	150 × 150 200 × 200 250 × 250 200 × 200 250 × 250 150 × 150 200 × 200 250 × 250 250 × 250 150 × 150 200 × 200 250 × 250 250 × 250	Beam Center Beam Top near Support Beam Bottom near Support	3 4 6 5 5 2 3 4 6 3 3 4 6	103 82 53 135 91 130 110 64 110 146 105 69 93	3.2 4.1 5.7 5.2 5.4 2.4 3.2 3.8 6.3 3.0 3.1 4.1 5.7	- - - - - - - - - - - - -	15 18 16 18 13 16 12 15 18 19 22 21 20	Flexure & Shear Failure Flexure & Shear Failure Flexure & Shear Failure Flexure & Shear Failure Flexure & Shear Failure Flexure & Shear Failure Flexure & Shear Failure Flexure & Shear Failure Flexure & Shear Failure Flexure & Shear Failure Flexure & Shear Failure Flexure & Shear Failure Flexure & Shear Failure	[82]
CB/NC CFRP-S-B NSM-CFRP-B CFRP-Lam.-B Hybrid-B/NC	0.4 0.4 0.4 0.4 0.4	250 × 300 250 × 300 250 × 300 250 × 300 250 × 300	21.0/3.2 21.0/3.2 21.0/3.2 21.0/3.2 21.0/3.2	2 × 4 2 × 4 2 × 4 2 × 4 2 × 4	D12C + D12T D12C + D12T D12C + D12T D12C + D12T D12C + D12T	Y-R8@100c/c Y-R8@100c/c Y-R8@100c/c Y-R8@100c/c Y-R8@100c/c	- EBR CFRP Sh. NSMCFRP-B EBR-CFRP-L EBRCFRP-BL	- 1 1 1 2	- 0.167 9.52 0.167 9.52/0.17	- Beam Bottom	- 1 Groove 1 Groove 1 Groove 1 Groove	2 × 2 2 × 2 2 × 2 2 × 2 2 × 2	- Beam Center Bottom Beam Center Bottom	18 27 20 20 16	85 94 100 93 107	56.15 47.35 59.4 41.9 58.86	- - - - -	22 19 24 20 22	Flexure Failure Flexure Failure Flexure Failure Flexure Failure Flexure Failure	[76]
Control-1/NC Control-2/NC SFR-DB-0.5-1 SFR-DB-0.5-2 SFR-DB-1.0-1 SFR-DB-1.0-2	2.5 2.5 2.5 2.5 2.5 2.5	200 × 600 200 × 600 200 × 600 200 × 600 200 × 600 200 × 600	39.0/3.5 39.0/3.5 41.3/3.7 41.3/3.7 46.1/3.8 46.1/3.8	2 × 5 2 × 5 2 × 5 2 × 5 2 × 5 2 × 5	D20C + D20T D20C + D20T D20C + D20T D20C + D20T D20C + D20T D20C + D20T	3–8 mm links in the shear zone just to support Compressive & Tensile Steel	- - - - - -	- - - - - -	- - - - - -	- - - - - -	No Opening Provided			476 535 640 660 720 710	1221 1273 1392 1375 1468 1389	8.15 7.55 10.4 10.4 12.75 13.5	8.15 7.55 10.4 10.4 12.75 13.5	14 18 19 15 22 20	Flexure Failure Flexure Failure Flexure Failure Flexure Failure Flexure Failure Flexure Failure	[44]
R-A/NC R-UA/NC N-C-A/NC N-G-A/NC N-P-A/NC E-C-A/NC E-G-A/NC E-P-A/NC N-C-UA/NC N-G-UA/NC N-P-UA/NC E-C-UA/NC E-G-UA/NC E-P-UA/NC	2.1 2.1 2.1 2.1 2.1 2.1 2.1 2.1 2.1 2.1 2.1 2.1 2.1 2.1	150 × 330 150 × 330 150 × 330 150 × 330 150 × 330 150 × 330 150 × 330 150 × 330 150 × 330 150 × 330 150 × 330 150 × 330 150 × 330 150 × 330	30.0/3.6 30.0/3.6 30.0/3.6 30.0/3.6 30.0/3.6 30.0/3.6 30.0/3.6 30.0/3.6 30.0/3.6 30.0/3.6 30.0/3.6 30.0/3.6 30.0/3.6 30.0/3.6	2 × 3 2 × 3 2 × 3 2 × 3 2 × 3 2 × 3 2 × 3 2 × 3 2 × 3 2 × 3 2 × 3 2 × 3 2 × 3 2 × 3	D8C + D16T D8C + D16T D8C + D16T D8C + D16T D8C + D16T D8C + D16T D8C + D16T D8C + D16T D8C + D16T D8C + D16T D8C + D16T D8C + D16T D8C + D16T D8C + D16T	Y-R6@215c/c Y-R6@215c/c Y-R6@215c/c Y-R6@215c/c Y-R6@215c/c Y-R6@215c/c Y-R6@215c/c Y-R6@215c/c Y-R6@215c/c Y-R6@215c/c Y-R6@215c/c Y-R6@215c/c Y-R6@215c/c Y-R6@215c/c	- - NSM-CFRP NSM-GFRP NSM-PBO EBR-CFRP EBR-GFRP EBR-PBO NSM-CFRP NSM-GFRP NSM-PBO EBR-CFRP EBR-GFRP EBR-PBO	- - 12 12 12 12 12 12 12 12 12 12 12 12	- - 0.047 0.066 0.0115 0.047 0.066 0.047 0.047 0.066 0.0115 0.047 0.066 0.047	- - Reinforcement provided on beam sides with a width of 120 mm FRP strips with 95 mm c/c distance.	- - 16.0 16.0 16.0 - - - 16.0 16.0 16.0 - - -	- - 20 × 120 20 × 120 20 × 120 - - - 20 × 120 20 × 120 20 × 120 - - -	- - Beam Sides Beam Sides Beam Sides - - - Beam Sides Beam Sides Beam Sides - - -	51 58 94 83 92 93 82 87 110 92 101 100 85 93	130 138 203 171 187 182 158 163 207 174 191 188 161 175	5.12 6.02 10.66 8.31 9.50 7.54 6.36 7.52 11.55 8.33 9.68 8.28 6.73 7.45	- - - - - - - - - - - - - -	23 21 26 22 27 29 28 20 23 25 19 30 22 21	Flexure & Concrete Crushing Flexure & Concrete Crushing Flexure & Concrete Crushing Flexure & Concrete Crushing Flexure & Concrete Crushing Flexure & Concrete Crushing Flexure & Concrete Crushing	[75]
R/NC R-S/NC R-FRP/NC T/NC T-FRP/NC	1.6 1.6 1.6 1.6 1.6	150 × 300 150 × 300 150 × 300 150 × 300 150 × 300	28.0/2.7 28.0/2.7 28.0/2.7 28.0/2.7 28.0/2.7	2 × 2 2 × 2 2 × 2 2 × 2 2 × 2	D14C + D14T D14C + D14T D14C + D14T D14C + D14T D14C + D14T	Y-R8@50c/c Y-R8@50c/c Y-R8@50c/c Y-R8@50c/c Y-R8@50c/c	- - NSM-CFRP Rope	- - 22 - 22	- - 28 mm^2^ - 28 mm^2^	- - Spider Web - Spider Web	No Opening Provided			70 82 91 97 111	140 164 182 194 221	10.3 5.1 4.8 5.3 5.1	10.3 5.1 4.8 5.3 5.1	13 9 3 13 15	Shear Failure Shear Failure Flexure Failure Shear Failure after Yielding Flex. Failure after Yielding	[58]
DBC/NC GI-N_1_-D_75_/NC GI-N_1_-D_150_/NC GI-N_1_-D_250_/NC GII-N_1_-D_75_/NC GII-N_2_-D_75_/NC GII-N_1_D_var_/NC GIIIN_2_D_75_R_o_ GIIIN_2_D_75_R_I_ GIIIN_2_D_75_R_II_ GIIIN_1_D_75_R_III_ GIVN_2_D_180_R_o_ GIVN_1_D_580_R_o_ GIVN_4_D_180_R_o_ GIVN_2_D_180_R_o_ GIVN_2_D_580_R_o_ GVN_4_D_180_R_o_ GVN_4_D_580_RI_3_ GVN_4_D_180_RI_4_ GVN_4_D_180_RI_5_ GVIN_4_D_580_R_o_ GVIN_4_D_580_RE_I_ GVIN_4_D_580_RE_I_ GVIN_4_D_580_RE_I_	1.1 1.1 1.1 1.1 1.1 1.1 1.1 1.1 1.1 1.1 1.1 1.1 1.1 1.1 1.1 1.1 1.1 1.1 1.1 1.1 1.1 1.1 1.1 1.1	80 × 400 80 × 400 80 × 400 80 × 400 80 × 400 80 × 400 80 × 400 80 × 400 80 × 400 80 × 400 80 × 400 80 × 400 80 × 400 80 × 400 80 × 400 80 × 400 80 × 400 80 × 400 80 × 400 80 × 400 80 × 400 80 × 400 80 × 400 80 × 400	25.0/3.1 25.0/3.1 25.0/3.1 25.0/3.1 25.0/3.1 25.0/3.1 25.0/3.1 25.0/3.1 25.0/3.1 25.0/3.1 25.0/3.1 25.0/3.1 25.0/3.1 25.0/3.1 25.0/3.1 25.0/3.1 25.0/3.1 25.0/3.1 25.0/3.1 25.0/3.1 25.0/3.1 25.0/3.1 25.0/3.1 25.0/3.1	2 bars on top, 4 bars on bottom, 4 bars of Ø8 mm @ 100c/c	D10C + D16T D10C + D16T D10C + D16T D10C + D16T D10C + D16T D10C + D16T D10C + D16T D10C + D16T D10C + D16T D10C + D16T D10C + D16T D10C + D16T D10C + D16T D10C + D16T D10C + D16T D10C + D16T D10C + D16T D10C + D16T D10C + D16T D10C + D16T D10C + D16T D10C + D16T D10C + D16T D10C + D16T	Y-R6@100c/c Y-R6@100c/c Y-R6@100c/c Y-R6@100c/c Y-R6@100c/c Y-R6@100c/c Y-R6@100c/c Y-R6@100c/c Y-R6@100c/c Y-R6@100c/c Y-R6@100c/c Y-R6@100c/c Y-R6@100c/c Y-R6@100c/c Y-R6@100c/c Y-R6@100c/c Y-R6@100c/c Y-R6@100c/c Y-R6@100c/c Y-R6@100c/c Y-R6@100c/c Y-R6@100c/c Y-R6@100c/c Y-R6@100c/c	- - - - - - - - - - - Steel Plate & Connectors Steel Plate & Connectors Steel Plate & Connectors Steel Plate & Connectors Steel Plate & Connectors Steel Plate & Connectors	- - - - - - - - - - 2 1 4 2 2 4 4 4 4 4 4 4 4 4	- - - - - - - - - - 80 × 180 80 × 580 80 × 180 80 × 180 80 × 180 80 × 180 80 × 180 80 × 180 80 × 180 80 × 180 80 × 180 80 × 180 80 × 180 80 × 180	- - - - - - - - - - Near Ops. Near Ops. Near Ops. Near Ops. Near Ops. Near Ops. Near Ops. Near Ops. Near Ops. Near Ops. Near Ops. Near Ops. Near Ops. Near Ops.	- 1 Circular 1 Circular 1 Circular 1 Circular 2 Circular 1 Circular 2 Circular 2 Circular 2 Circular 2 Circular 2 Rectangle 1 Rectangle 4 Rectangle 2 Rectangle 2 Rectangle 4 Rectangle 4 Rectangle 4 Rectangle 4 Rectangle 4 Rectangle 4 Rectangle 4 Rectangle 4 Rectangle	- Ø75 Ø150 Ø250 Ø75 Ø75 Var. Ø75 Ø75 Ø75 Ø75 180 × 80 580 × 80 180 × 80 180 × 240 580 × 80 180 × 80 180 × 80 180 × 80 180 × 80 180 × 80 180 × 80 180 × 80 180 × 80	- - - - - - - - - - - Additional Steel Plate, along with connectors, was provided around the openings of the beam depending on the shape and size of the opening	110 97 85 34 97 104 57 104 104 104 105 42 41 63 39 - 63 75 77 81 64 81 85 89	313 276 241 98 276 297 162 298 298 298 299 119 118 181 110 116 181 215 220 231 183 232 244 255	1.5 1.8 2.1 1.8 1.8 2.3 1.6 2.3 1.6 1.6 2.0 0.7 0.4 3.4 2.9 0.02 3.4 4.4 4.7 5.7 3.5 4.9 5.1 5.5	- - - - - - - - - - - - - - - - - - - - - - - -	8 6 7 5 6 8 9 5 10 6 8 9 8 6 7 5 6 4 6 9 8 5 8 9	Shear Compression Failure Shear Compression Failure Shear Compression Failure Shear Compression Failure Shear Compression Failure Shear Compression Failure Shear Compression Failure Shear Compression Failure Shear Compression Failure Shear Compression Failure Shear Compression Failure Shear Compression Failure Shear Compression Failure Shear Compression Failure Shear Compression Failure Shear Compression Failure Shear Compression Failure Shear Compression Failure Shear Compression Failure Shear Compression Failure Shear Compression Failure Shear Compression Failure Shear Compression Failure Shear Compression Failure	[77]
CTRL-1P/NC CTRL-2P/NC G1-SN-1P/NC G1-SN-2P/NC G2SL-1P/LC G2SL-2P/LC G2SN-1P/SFC G2-SN-2P G3-NSN-1P G3-NSN-2P G3-NS1N-2P	2.0 2.0 2.0 2.0 2.0 2.0 2.0 2.0 2.0 2.0 2.0	180 × 450 180 × 450 180 × 450 180 × 450 180 × 450 180 × 450 180 × 450 180 × 450 180 × 450 180 × 450 180 × 450	65.0/2.4 65.0/2.4 65.0/2.4 65.0/2.4 31.0/2.3 31.0/2.3 37.0/7.7 37.0/7.7 37.0/7.7 37.0/7.7 37.0/7.7	2 × 3 2 × 3 2 × 3 2 × 3 2 × 3 2 × 3 2 × 3 2 × 3 2 × 3 2 × 3 2 × 3	D10C + D16T D10C + D16T D10C + D16T D10C + D16T D10C + D16T D10C + D16T D10C + D16T D10C + D16T D10C + D16T D10C + D16T D10C + D16T	Y-R8@130c/c Y-R8@130c/c Y-R8@130c/c Y-R8@130c/c Y-R8@130c/c Y-R8@130c/c Y-R8@130c/c Y-R8@130c/c Y-R8@130c/c Y-R8@130c/c Y-R8@130c/c	No Additional Reinforcement or Strengthening Material Provided				No Opening Provided			70 80 80 80 80 60 80 80 80 70 80	410 542 503 688 502 717 552 752 592 787 671	13.76 12.24 15.44 22.02 18.39 21.2 25.6 29.2 24.46 35.39 30.19	55 69 66.3 70.7 58 80 62.6 78 72 85.7 77.4	16 14 19 26 20 16 20 18 14 15 17	Flexure Shear Failure Flexure Shear Failure Semi Flexure Failure Flexure Shear Failure Flexure Failure Flexure Failure Flexure Failure Flexure Failure Flexure Failure Flexure Failure Flexure Failure	[80]
NS-200-C/NC NS-250-C/NC FS-200-C/NC FS-250-C/NC NS-150-T/NC NS-200-T/NC NS-250-T/NC FS-250-T/NC NS-150-B/NC NS-200-B/NC NS-250-B/NC FS-250-B/NC	1.2 1.2 1.2 1.2 1.2 1.2 1.2 1.2 1.2 1.2 1.2 1.2	80 × 500 80 × 500 80 × 500 80 × 500 80 × 500 80 × 500 80 × 500 80 × 500 80 × 500 80 × 500 80 × 500 80 × 500	36.0/3.6 36.0/3.6 36.0/3.6 36.0/3.6 36.0/3.6 36.0/3.6 36.0/3.6 36.0/3.6 36.0/3.6 36.0/3.6 36.0/3.6 36.0/3.6	2 bars in top, 4 bars in bottom, 6 bars of Ø6 mm @ 150c/c	D8C + D14T D8C + D14T D8C + D14T D8C + D14T D8C + D14T D8C + D14T D8C + D14T D8C + D14T D8C + D14T D8C + D14T D8C + D14T D8C + D14T	Y-R6@150c/c Y-R6@150c/c Y-R6@150c/c Y-R6@150c/c Y-R6@150c/c Y-R6@150c/c Y-R6@150c/c Y-R6@150c/c Y-R6@150c/c Y-R6@150c/c Y-R6@150c/c Y-R6@150c/c	- - EBR-CFRP Sheet EBR-CFRP Sheet - - - EBR-CFRP Sheet -	- - 8 8 - - - 8 - - - 8	- - 0.167 0.167 - - - 0.167 - - - 0.167	- - Reinforcement provided along the openings of the beam in horizontal as well as vertical directions	2 Rectangle 2 Rectangle 2 Rectangle 2 Rectangle 2 Rectangle 2 Rectangle 2 Rectangle 2 Rectangle 2 Rectangle 2 Rectangle 2 Rectangle 2 Rectangle	200 × 200 250 × 250 200 × 200 250 × 250 150 × 150 200 × 200 250 × 250 250 × 250 150 × 150 200 × 200 250 × 250 250 × 250	Middle of Shear Span Top of Shear Span near support Bottom of Shear Span near support	57 39 95 64 91 77 45 77 102 74 48 65	163 110 271 182 260 220 128 220 292 211 138 187	4.1 5.7 5.2 5.4 2.4 3.2 3.8 6.3 3.0 3.1 4.1 5.7	- - - - - - - - - - - -	12 10 13 12 14 11 9 10 12 13 13 11	Flexure Shear Failure Flexure Shear Failure Flexure Shear Failure Flexure Shear Failure Flexure Shear Failure Flexure Shear Failure Flexure Shear Failure Flexure Shear Failure Flexure Shear Failure Flexure Shear Failure Flexure Shear Failure Flexure Shear Failure	[72]
RB/NC B19/NC B20/NC B21/NC B32/NC B33/NC	2.5 2.5 2.5 2.5 2.5 2.5	120 × 250 120 × 250 120 × 250 120 × 250 120 × 250 120 × 250	31.2/3.4 31.2/3.4 31.2/3.4 31.2/3.4 31.2/3.4 31.2/3.4	2 × 2 2 × 2 2 × 2 2 × 2 2 × 2 2 × 2	D16C + D16T D16C + D16T D16C + D16T D16C + D16T D16C + D16T D16C + D16T	YR4.2@180c/c YR4.2@180c/c YR4.2@180c/c YR4.2@180c/c YR4.2@180c/c YR4.2@180c/c	EBR-CFRP Laminates (L Shaped)	- 11 11 11 11 11	- 0.167 0.167 0.167 0.167 0.167	- CFRP was applied in a U-shape with overlap at the bottom	- 11 Rectangle 11 Rectangle 11 Rectangle 11 Rectangle 11 Rectangle	- 20 × 20 20 × 20 20 × 20 20 × 20 20 × 20	- Applied all along the beam with 180 mm c/c spacing	27 47 40 40 40 40	78 116 115 114 115 115	1.11 1.93 3.99 4.71 4.80 2.63	- - - - - -	19 21 16 18 20 22	Shear Failure Flexure Failure Flexure Failure Flexure Failure Shear Failure Shear Failure	[65]
BO/NC BC1/NC BA1/NC BC2/NC BA2/NC BA3/NC	1.9 1.9 1.9 1.9 1.9 1.9	120 × 300 120 × 300 120 × 300 120 × 300 120 × 300 120 × 300	20.0/- 20.0/- 20.0/- 20.0/- 20.0/- 20.0/-	2 × 2 2 × 2 2 × 2 2 × 2 2 × 2 2 × 2	D10C + D16T D10C + D16T D10C + D16T D10C + D16T D10C + D16T D10C + D16T	Y-R6@200c/c Y-R6@200c/c Y-R6@200c/c Y-R6@200c/c Y-R6@200c/c Y-R6@200c/c	High Strength Jackets-SFRC High Strength Jackets-SFRC High Strength Jackets-SFRC	2 2 2 2 2 2	30.0 30.0 30.0 30.0 30.0 30.0	- - - - - -	No Openings Provided			20 32 35 46 31 40	89 148 168 195 160 185	4.47 4.81 5.9 6.9 5.5 6.67	12.16 2.94 7.32 17.18 6.79 16.77	9 8 9 11 7 7	Diagonal Tension Failure Diagonal Tension Failure Diagonal Tension Failure Diagonal Tension Failure Diagonal Tension Failure Diagonal Tension Failure	[79]
BP-1a/NC BP-1b/NC BP-3a/NC BP-1e/NC BP-3e/NC BN-1a/NC BN-1b/NC BN-1b (2)/NC BN-2a/NC BN-3a/NC BN-3b/NC BC-1a/NC N1-1.0-WO N1-1.0-WV N1-1.0-WVH N1-1.5-WO N1-1.5-WV N1-1.5-WVH	1.8 1.8 1.8 1.8 1.8 1.8 1.8 1.8 1.8 1.8 1.8 1.8 1.8 1.8 1.8 1.8 1.8 1.8	150 × 600 150 × 600 150 × 600 150 × 600 150 × 600 150 × 600 150 × 600 150 × 600 150 × 600 150 × 600 150 × 600 150 × 600 150 × 600 150 × 600 150 × 600 150 × 600 150 × 600 150 × 600	39.0/- 38.0/- 39.0/- 38.0/- 40.0/- 37.0/- 40.0/- 37.0/- 37.0/- 37.0/- 40.0/- 37.0/- 37.0/- 37.0/- 38.0/- 37.0/- 37.0/- 37.0/-	2 × 2 2 × 2 2 × 2 2 × 2 2 × 2 2 × 4 2 × 4 2 × 4 2 × 4 2 × 4 2 × 4 2 × 2 2 × 2 2 × 2 2 × 2 2 × 2 2 × 2 2 × 2	D10C + D22T D10C + D22T D10C + D22T D10C + D22T D10C + D22T D10C + D22T D10C + D22T D10C + D22T D10C + D22T D10C + D22T D10C + D22T D10C + D22T D10C + D22T D10C + D22T D10C + D22T D10C + D22T D10C + D22T D10C + D22T	Y-R10@150c/c Y-R10@150c/c Y-R10@150c/c Y-R10@150c/c Y-R10@150c/c Y-R10@150c/c Y-R10@150c/c Y-R10@150c/c Y-R10@150c/c Y-R10@150c/c Y-R10@150c/c Y-R10@150c/c Y-R10@150c/c Y-R10@150c/c Y-R10@150c/c Y-R10@150c/c Y-R10@150c/c Y-R10@150c/c	R-Cage & Ten. R-Cage & Ten. R-Cage & Ten. R-Cage & Ten. R-Cage & Ten. R-Cage & Ten. R-Cage & Ten. R-Cage & Ten. R-Cage & Ten. R-Cage & Ten. R-Cage & Ten. R-Cage & Ten. R-Cage & Ten. R-Cage & Ten. R-Cage & Ten. R-Cage & Ten. R-Cage & Ten. R-Cage & Ten.	3 3 3 3 3 3 3 3 3 3 3 3 3 3 3 3 3 3	3Ø10,T22 3Ø10,T22 3Ø10,T22 3Ø10,T22 3Ø10,T22 3Ø10 3Ø10 3Ø10 3Ø10 3Ø10 3Ø10 3Ø10 3Ø10 3Ø10 3Ø10 3Ø10 3Ø10 3Ø10	- - - - - - - - - - - - - - - - - -	1 Circle 1 Circle 1 Circle 1 Circle 1 Circle - - - - - - - - - - - - -	Ø80 mm Ø80 mm Ø80 mm Ø80 mm Ø80 mm - - - - - - - - - - - - -	- - - - - - - - - - - - - - - - - -	175 225 175 250 250 150 100 175 120 150 100 175 225 225 225 125 150 100	580 400 876 360 950 375 410 285 875 835 875 640 800 910 975 320 525 700	3.1 4.6 2.9 2.8 3.4 5.2 12.1 10.6 5.9 7.8 10.8 9.2 11.6 7.7 9.2 8.9 10.3 9.4	- - - - - - - - - - - - - - - - - -	20 18 13 17 19 20 19 16 16 22 19 32 14 19 21 17 29 20	Diagonal Flexure Failure Diagonal Flexure Failure Diagonal Flexure Failure Diagonal Flexure Failure Diagonal Flexure Failure Diagonal Flexure Failure Diagonal Flexure Failure Diagonal Flexure Failure Diagonal Flexure Failure Diagonal Flexure Failure Diagonal Flexure Failure Diagonal Flexure Failure Diagonal Flexure Failure Diagonal Flexure Failure Diagonal Flexure Failure Diagonal Flexure Failure Diagonal Flexure Failure Diagonal Flexure Failure	[78]
CB1/NC CB2/NC RB1-1/NC RB1-3/NC RB1-2/NC RB2-1/NC RB2-3/NC RB2-2/NC RB3-1/NC RB3-2/NC	1.8 1.8 1.8 1.8 1.8 1.8 1.8 1.8 1.8 1.8	200 × 500 200 × 500 200 × 500 200 × 500 200 × 500 200 × 500 200 × 500 200 × 500 200 × 500 200 × 500	23.8/3.0 23.8/3.0 23.8/3.0 23.8/3.0 23.8/3.0 23.8/3.0 23.8/3.0 23.8/3.0 23.8/3.0 23.8/3.0	2 bars at top, 2 bars at bottom 6 bars of Ø12 mm @ 85c/c	D14C + D14T D14C + D14T D14C + D14T D14C + D14T D14C + D14T D14C + D14T D14C + D14T D14C + D14T D14C + D14T D14C + D14T	Y-R8@100c/c Y-R8@100c/c Y-R8@100c/c Y-R8@100c/c Y-R8@100c/c Y-R8@100c/c Y-R8@100c/c Y-R8@100c/c Y-R8@100c/c Y-R8@100c/c	- - EBR-CFRP Sheet EBR-CFRP Sheet EBR-CFRP Sheet EBR-CFRP Sheet	- - 1 1 1 1 1 1 1 1	0.17 0.17 0.17 0.17 0.17 0.17 0.17 0.17 0.17 0.17	- - U-shaped CFRP Sheet was provided from the neutral axis of the beam to the beam bottom	No Openings Provided			200 182 95 104 93 120 113 120 120 123	512.2 485.6 488.4 561.3 485 400 433 439 456 449	17.8 12.6 2.8 17.3 20.2 23.6 22.8 17.2 14.9 15.7	- - - - - - - - - -	17 14 16 15 19 18 15 22 21 20	Flexural Failure Flexural Failure FRP De-bonding Concrete Peeling at Support Flexure and Concrete Peeling Flexure Failure & Rupture Flexure Failure & Rupture Flexure Failure & Rupture Flexure Failure & Rupture	[66]
B1/NC B2/NC B3/NC B4/NC B5/NC	4.02 4.02 4.02 4.02 4.02	254 × 356 254 × 356 254 × 356 254 × 356 254 × 356	27.6/3.2 27.6/3.2 27.6/3.2 27.6/3.2 27.6/3.2	4 × 4 4 × 4 4 × 4 4 × 4 4 × 4	D13C + D13T D13C + D13T D13C + D13T D13C + D13T D13C + D13T	Y-R10@254c/c Y-R10@254c/c Y-R10@254c/c Y-R10@254c/c Y-R10@254c/c	- Carbon Fibers Splay Anchors Carbon Fibers Splay Anchors	- 6 6 6 6	- 12.7 12.7 12.7 12.7	- - - - -	No Openings Provided			35 34 34 35 35	89 105 120 146 155	69.9 34.3 55.4 26.7 25.4	- - - - -	22 28 25 27 29	Flexural Failure Flexural Failure Flexural Failure Flexural Failure Flexural Failure	[67]
